# Modulating the Interfacial Energy of Ni–Bi Molten Alloys for Enhanced Methane Decomposition to Hydrogen

**DOI:** 10.1021/acscatal.5c02867

**Published:** 2025-10-03

**Authors:** Zhao Sun, Bin Wang, Nicholas F. Dummer, Haifeng Qi, Louise R. Smith, Zhiqiang Sun, Graham J. Hutchings

**Affiliations:** † Hunan Engineering Research Center of Clean and Low-Carbon Energy Technology, School of Energy Science and Engineering, 12570 Central South University, Changsha 410083, China; ‡ Max Planck-Cardiff Center on the Fundamentals of Heterogeneous Catalysis FUNCAT, Cardiff Catalysis Institute, School of Chemistry, 2112 Cardiff University, Cardiff CF24 4HQ, U.K.

**Keywords:** methane decomposition, hydrogen production, molten alloy catalysts, NiMn–Bi, molecular dynamics simulations

## Abstract

Catalytic methane decomposition is a highly promising CO_2_-free hydrogen production technology with carbon material generation; however, developing catalysts that can efficiently decompose methane at moderate temperatures remains challenging. In this study, we develop a series of NiMn–Bi molten alloy catalysts with various Ni:Mn ratios for catalyzing methane decomposition. The Mn-modified Ni–Bi alloy exhibits a CH_4_ conversion of 15.3% at 850 °C, and the corresponding hydrogen production rate increases by 112% compared with Ni–Bi. The ternary alloy catalyst also demonstrates stability at this production rate for up to 80 h. Molecular dynamics simulations show that the introduction of Mn significantly reduces the strong interaction between the active metal Ni and the solvent metal Bi, thereby accelerating the methane dissociation rate. More importantly, among the theoretically calculated binding energy, interfacial energy, Ni–Bi interaction, and mean-square displacement, interfacial energy, a comprehensive demonstration of surface energy and atomic interactions, is proposed as a potential descriptor for predicating and estimating the catalytic performance of the molten alloy-based catalysts.

## Graphical abstract

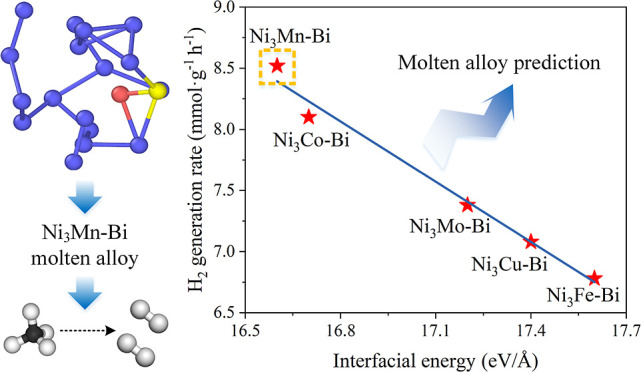

## 1 Introduction

With the transformation of global energy structures and the increasingly serious issue of environmental pollution, the development and use of clean energy with less or no carbon emission has become an important direction for scientific research and industrial application.^1,2^ Among various clean energy sources, hydrogen is regarded as a key energy carrier in future energy systems due to its advantages in high energy density, versatility, and reduction in greenhouse gas emissions, for example.^3–5^ Approximately 90% of the current annual global hydrogen production comes from the processing of fossil fuels, ordinarily going through reforming, water–gas shift, and pressure swing adsorption, which are complicated and inefficient with large amounts of CO_2_ released.^6–8^ Therefore, the development of CO_2_ emission-free hydrogen production technologies is of great importance to accomplish sustainable energy supplies and reduce greenhouse gas emissions.^9^

Catalytic methane decomposition (CMD) is a potential technology for hydrogen production due to the advantages of generating H_2_ in one step, no CO_*x*_ emission, and easy separation of H_2_ and the carbon byproduct.^10–12^ Despite the significant theoretical advantages of this methane decomposition technology, major challenges remain to reach commercialization.^13,14^ First, a high temperature is required, leading to high energy consumption and equipment cost.^15,16^ To overcome this challenge, researchers have sought efficient catalysts to reduce the methane decomposition temperature.^17–19^ Conventional transition metal-based solid catalysts, such as iron, cobalt, and nickel, can efficiently promote the C–H bond activation, thus accelerating the dehydrogenation rate and significantly lowering the methane decomposition temperature; however, they are prone to deactivation due to the high-temperature environments.^20–22^ Furthermore, the accumulation of deposited carbon on the active sites of the catalysts reduces activity.^23^ Therefore, the catalyst and deposited carbon need to be separated periodically. Of the various approaches that have been reported such as mechanical separation, magnetic separation, thermal treatment, and chemical dissolution, further improvements are still necessary.^24–27^

More recently, molten metal-based catalysts have attracted wide attention for their satisfactory stability at high temperatures and a high resistance to carbon buildup.^28–30^ The ability of molten metal catalysts to remain in a liquid state facilitates dynamic separation of the deposited carbon and the liquid molten-state catalyst, thus avoiding rapid deactivation.^31^ On this basis, alloying has been reported to be an effective strategy for boosting the CMD performance, originating from the alteration of their structure and electron arrangement.^32–34^ Upham et al.^35^ achieved a 95% methane conversion by a 27 mol % Ni with 73 mol % Bi alloy in a bubble tower with a height of 1.1 m at 1065 °C with no carbon dioxide or other gaseous byproduct production. The active metal atoms in the molten alloy were reported to be dispersed and negatively charged, and the amount of charge on the atoms could be correlated to their catalytic activity. More recently, Chen et al.^36^ reported a ternary NiMo–Bi alloy (2.3 wt % Ni, 1.3 wt % Mo, and 96.4 wt % Bi), which achieved 100% H_2_ selectivity over 120 h of stable decomposition at 800 °C under a methane flow rate of 4 mL min^–1^. In the Ni–Bi alloy system, it was reported that the positively charged Bi would encapsulate catalytically active and negatively charged Ni, whereby access for CH_4_ to the active sites would be obstructed.^37^ The strong interactions between Ni and Mo would modulate the electronic state of Ni and reduce the number of interactions between Ni and Bi, thereby revealing more active sites and enhancing catalytic activity.

Herein, we propose that ternary or even multialloy-based catalysts would be potential selections for further promotion of low-temperature CMD. Therefore, Fe-, Co-, W-, Cr-, Cu-, Mo-, and Mn-modified Ni–Bi alloys were theoretically and experimentally explored to reveal the intrinsic descriptors for potential catalyst prediction via high-throughput screening. First, we revealed the effect of ternary alloy components on the CMD activity. The Ni_3_Mn–Bi alloy demonstrates better low-temperature adaptability compared with most of the reported molten metal catalysts, exhibiting a methane conversion of 15.3% at 850 °C, and this maintained excellent activity for up to 80 h. Second, we used molecular dynamics (MD) simulations to rationalize the intrinsic promotion mechanism of ternary alloy catalysts during CMD. We show that Mn introduction modulated the catalyst’s binding energy and weakened the strong interaction between Ni and Bi, thus breaking through the surrounding Bi atoms and exposing more active sites. Third, we compared five potential descriptors for estimating the CMD performance, which includes binding energy, interfacial energy, radial distribution function (RDF), Ni–Bi interaction, and mean-square displacement (MSD). Among them, interfacial energy and Ni–Bi interactions better align with the performance results, contributing to the prediction of catalyst activity.

## 2 Materials and Methods

### 2.1 Catalyst Preparation

Ni–Bi and NiMn–Bi catalysts were synthesized via a melt method by controlling the molar ratio of a mixture of nickel metal (Ni, 99.9%), bismuth (Bi, 99.99%), and manganese (Mn, 99.9%). Ni–Bi was mixed in a mole ratio of 0.27:0.73, and the ternary Ni_*x*_Mn_*y*_-Bi alloy catalysts were prepared by adding different mole ratios of Mn in accordance with *x*/*y* = 6:1, 4:1, 3:1, 2:1, 3:2, 1:1, or 1:2. All the metal feedstock was purchased in powder form from Aladdin and sieved through a screen to ensure uniform particle size before use. The mixture was then heated to 850 °C for 2 h to ensure complete melting and homogenization.

### 2.2 Characterizations

The N_2_ adsorption–desorption isotherm at −196 °C was determined by using a Micromeritics ASAP 2460 analyzer. Prior to the measurement, the solid carbon sample was purged under a N_2_ atmosphere at 200 °C for 6 h to remove irrelevant components adsorbed on the solid carbon. The isotherm was then measured to obtain the isotherm. The pore size distribution and specific surface area of the solid carbon were calculated using the Barrett–Joyner–Halenda (BJH) and Brunauer–Emmet–Teller (BET) methods, respectively.

Solid carbon samples were subjected to an oxygen-programmed temperature-raising oxidation reaction (O_2_-TPO) using a Micromeritics Auto Chem II 2920 chemisorption analyzer. In a typical experiment, 0.300 ± 0.0005 g of the sample was placed in a U-shaped reactor, preheated in an Ar (30 mL/min) atmosphere at 200 °C for 30 min to remove surface impurities, and then cooled down to 30 °C and held for 20 min. After the baseline was stabilized, the gas flow was switched to a 30 mL/min mixture of 10 vol % O_2_ to 90 vol % Ar, and the temperature was increased to 800 °C under 10 °C/min. The oxygen consumption signal was detected and recorded by a thermal conductivity detector, and a mass spectrometer was also connected to monitor the real-time signals of CO and CO_2_.

X-ray diffraction (XRD) was performed using a PANalytical Empyrean diffractometer equipped with a Cu Kα radiation source (λ = 1.5418 Å) at 40 kV and 40 mA. Where scan rate = 8°/min, step size = 0.02°, and X-ray diffractograms were recorded in the range of 10°–90°.

In situ XRD experiments were operated with a diffractometer equipped with a high-temperature reaction cell (XRK 900, Anton Paar GmbH). In the high-temperature experiments, the powders were sealed with epoxy adhesive in an inert gas atmosphere after being loaded into a 13.8 mm sample tank in order to avoid accidental oxidation reactions during heating. The diffraction peaks were recorded from 50 to 780 °C.

The structure of the alloy catalysts and the degree of graphitization of the solid carbon products were characterized by Raman spectroscopy using a Renishaw inVia Raman spectrometer equipped with a 532 nm laser. Three measurements were taken for each sample.

X-ray photoelectron spectroscopy (XPS) measurements were performed by using a Thermo Scientific k-Alpha^TM+^ spectrometer equipped with a monochromatic Al Kα X-ray source (1486.6 eV) to analyze the chemical state of the catalyst. The binding energy was calibrated by using the C 1s (284.8 eV) peak as a reference.

A TESCAN MIRA3 LMH scanning electron microscope was used to analyze the surface morphology of the catalyst, as well as the solid carbon collected after the reaction. In addition, energy dispersive X-ray spectroscopy (EDS) analysis was combined to determine the distribution of elements on the catalyst surface as well as the metal component existing on the deposited carbon.

A Fel Tecnai G2 F300 field emission transmission electron microscope was used to observe the microstructure of the catalyst, and the solid carbon was recovered. The maximum magnification was 1.05 million times, the accelerating voltage was 300 kV, the energy resolution of the X-ray spectrometer was 130 eV, and the elemental analysis range of the X-ray spectrometer was from B5 to U92. The distributions of Ni, Mn, Bi, and C in the samples were analyzed by energy-dispersive X-ray (EDS).

### 2.3 Experimental Conditions

The CMD reaction was conducted in a round-bottomed test tube with an outer diameter of 18 mm, an inner diameter of 15 mm, and a length of 400 mm, and the reactor was placed in a temperature-controlled electric heating furnace (Figure S1). All catalyst synthesis steps, including metal mixing, melting, and cooling, were conducted under a strict inert atmosphere. In a typical test, 10.000 ± 0.0005 g of mixed solid powder was placed into a quartz tube reactor with a catalyst height of approximately 35 mm. Prior to the experiment, the reactor was purged with 15 mL/min N_2_ for 20 min to exclude oxygen and other impurities. Then, the reaction temperature was increased from 20 °C to the target temperature (800 °C, 850 °C, 900 °C, 950 °C, or 1000 °C) at a 10 °C/min ramp rate with the pressure maintained at an atmospheric pressure. Once the temperature was stabilized, the reaction atmosphere was switched to a mixture of CH_4_ and N_2_ (CH_4_/N_2_ = 40 vol %/60 vol %) with a total flow rate of 25 mL/min. The density difference between the molten-metal catalyst and the deposited carbon allows for easy separation, eliminating the need for catalyst regeneration. The postreaction cooling process was also conducted under N_2_ for at least 2 h to further protect the sample from ambient air oxidation. The reaction products were collected and analyzed by an online gas chromatograph (INFICON Micro GC Fusion) equipped with two TCDs. All experiments were performed twice to ensure reproducibility.

For the kinetic analysis, five temperature points, 800 °C, 850 °C, 900 °C, 950 °C, and 1000 °C, were selected. To minimize the mass transfer effect as much as possible, all kinetic measurements were conducted under low methane conversion conditions (≤15.3%) to demonstrate the intrinsic kinetic characteristics of the catalysts.

After CH_4_ decomposition, the catalyst-solid carbon mixture was slowly cooled to ambient temperature under a N_2_ atmosphere, and the resulting carbon was recovered from the bubble column. To collect the carbon products from the cooled mixture, the recovered mixture was washed by hydrochloric acid for three times and DI water for five times, respectively, and collected by filtration. The resulting carbon was rinsed with ethanol and dried at 100 °C overnight.

### 2.4 Data Analysis

The total methane decomposition product gas flow rate is calculated by

E1 $$Y\left(total,\;out\right)=\frac{Y\left(N_{2},\;out\right)}{C\left(N_{2},\;out\right)}$$

where *Y*(total, out) is the total outlet gas flow rate and *Y*(N_2_, out) denotes the outlet N_2_ flow rate. The production rate of component *i* is calculated according to

E2 Y(i,out)=Y(total,out)×C(i,out)

where *Y*(*i*, out) and *C*(*i*, out) represent the flow rate and concentration, respectively, of component *i* in the methane decomposition product at the reactor outlet; *i* could be CH_4_, H_2_, CO, or CO_2_. Methane conversion is calculated as

E3 $$X\left({CH}_{4}\right)=\frac{Y\left({CH}_{4},in\right)-Y\left({CH}_{4},out\right)}{Y\left({CH}_{4},in\right)}\times 100\%$$

where *X*(CH_4_) is the methane conversion (%), *Y*(CH_4_, out) represents the outlet methane flow rate, and *Y*(CH_4_, in) represents the inlet methane feeding rate. H_2_ generation rate is calculated as

E4 $$Y\left(H_{2},out\right)=Y\left(total,out\right)\times C\left(H_{2},out\right)$$

The relationship between the reaction rate constant and temperature is expressed by the Arrhenius formula:

E5 $$k={Ae}^{-E_{a}/RT}$$

where *k* denotes the reaction rate constant, *R* represents the ideal gas constant, *A* is the pre-exponential factor, and *E*_a_ represents the activation energy of the catalyst. Taking the logarithms of both sides of the equation simultaneously yields

E6 $$\ln k=\ln A-\frac{E_{a}}{RT}$$

### 2.5 Molecular Dynamics Simulation Details

All molecular dynamics (MD) simulations were performed using the LAMMPS simulation software. MD simulations are based on a fundamental understanding of the dynamics and equilibrium properties of molecular systems based on statistical mechanics and classical Newtonian equations of motion.^38,39^ To simulate the molten Ni–Bi and NiMn–Bi systems as well as the catalyst methane decomposition process, we performed NVT MD simulations to keep the temperature constant.

We used a 10 × 10 × 10 simulation box with a cubic system containing Ni–Bi with 12 Ni and 113 Bi atoms and Ni_3_Mn–Bi with 9 Ni, 3 Mn, and 113 Bi atoms. First, the rapid melting process of the catalysts was simulated at temperatures ranging from 300 to 2000 K to obtain the melting temperature of the catalysts. Then, the MD simulations of the catalyst were carried out at a temperature of 1500 K for 2000 fs in order to observe the bond-breaking and bond-forming processes of the catalyst. Finally, we performed MD simulations of methane decomposition of Ni–Bi and Ni_3_Mn–Bi catalysts embedded at 1500 K to observe the gradual C–H bond cleavage in the methane molecule–catalyst system. The temperature simulated was from 1100 to 1300 K.

## 3 Results and Discussion

A series of Ni–Mn–Bi catalysts were prepared; inductively coupled plasma–mass spectrometry (ICP–MS) results confirmed that the elemental compositions of Ni, Mn, and Bi align with the theoretical values (Table 1).

**Table 1:** ICP–MS Analysis of Ni-to-Mn Ratios in Different Catalysts

catalyst	theoretical Ni/Mn ratios	measured Ni/Mn ratios
Ni_6_Mn–Bi	6.00	5.97
Ni_4_Mn–Bi	4.00	3.92
Ni_3_Mn–Bi	3.00	3.01
Ni_2_Mn–Bi	2.00	1.96
Ni_3_Mn_2_–Bi	1.50	1.58
NiMn–Bi	1.00	0.93
NiMn_2_–Bi	0.50	0.56

We investigated the effect of temperatures and ternary alloy components (W, Mo, Mn, Co, Fe, Cr, or Cu) on the CH_4_ conversion and H_2_ generation rate (Figures S2–S6). The results showed that the CH_4_ conversion after Mn doping was enhanced by 46.0% compared with that of Ni–Bi. Moreover, the methane conversion over the Ni_3_Mn–Bi catalyst increased by 26.3%, 11.6%, 2.7%, 16.1%, 24.4%, and 13.8% compared with W, Mo, Co, Fe, Cr, and Cu, respectively, at 850 °C. Their corresponding hydrogen evolution rates show a similar trend with CH_4_ conversion. Therefore, the NiMn–Bi-based catalysts were selected for further investigation with a fixed Ni/Bi mole ratio of 0.27:0.73 (Figures S7–S11).

The effect of Ni-to-Mn ratios on the CMD performance was explored (Figure 1a,b). The CH_4_ conversions and hydrogen generation rates over Ni–Bi and Mn–Bi were 8.2% and 4.02 mmol g^–1^ h^–1^ as well as 5.9% and 1.68 mmol g^–1^ h^–1^ at 850 °C, while those of Ni_3_Mn–Bi were much higher, corresponding to 15.3% and 9.30 mmol g^–1^ h^–1^. Moreover, both CH_4_ conversion and H_2_ generation rate first increase and then decrease with the Mn-to-Ni mole ratios from 1:2 to 6:1. The Ni–Mn molar ratio of 3:1 shows the best CMD performance, indicating the promoting effect of an appropriate Mn concentration. The decrease in the catalytic activity under high Mn introduction (Ni/Mn < 1:3) may be attributed to the dilution of active sites, indicating that although Mn modification enhances activity, excessive Mn content can be detrimental.

**Figure 1:**
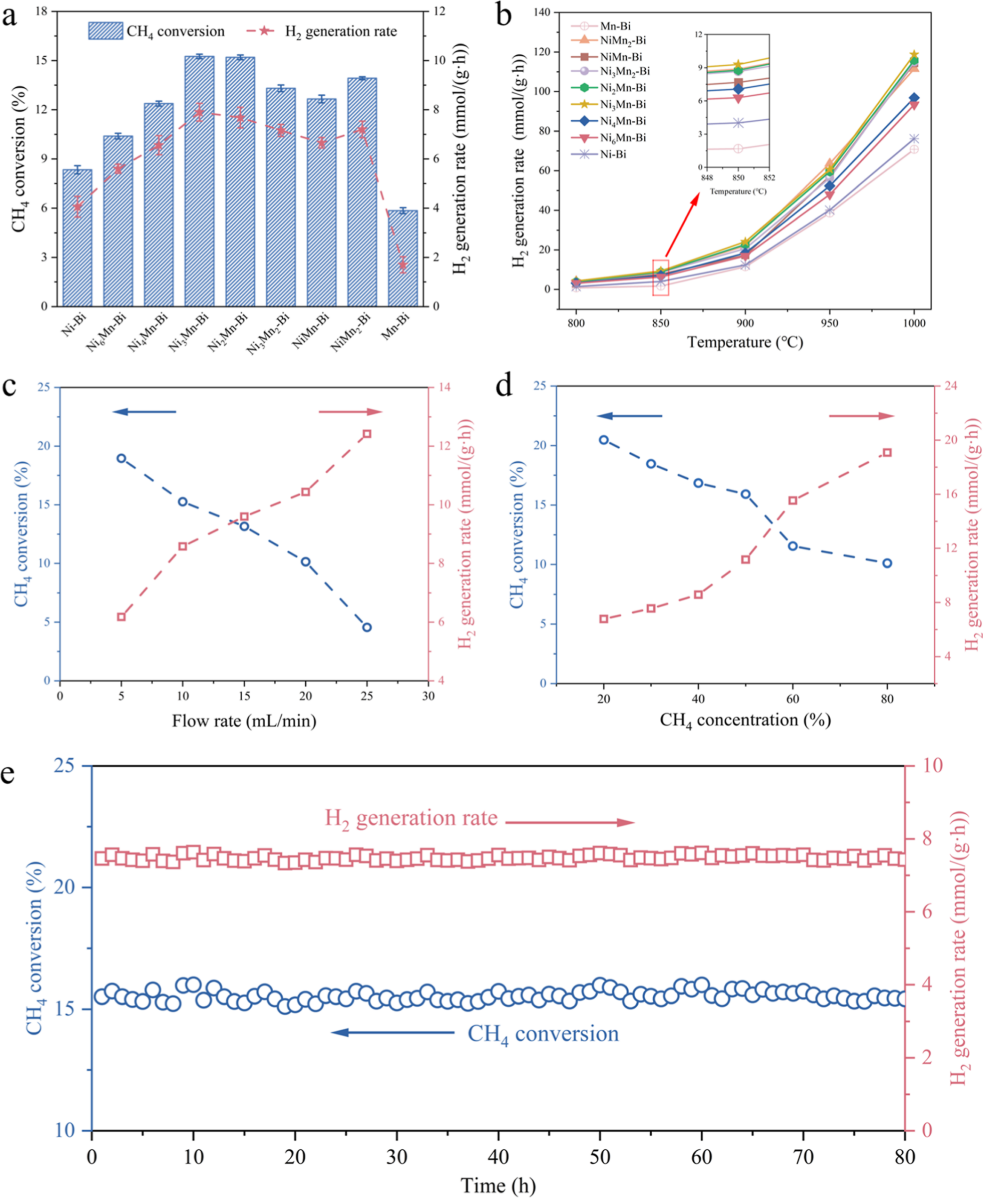
Catalytic performance over liquid alloy catalysts under different reaction conditions. (a) CH_4_ conversion (reaction temperature = 850 °C, N_2_ = 15 mL min^–1^, CH_4_ = 10 mL min^–1^). (b) H_2_ generation rate (N_2_ = 15 mL min^–1^, CH_4_ = 10 mL min^–1^). (c) CH_4_ conversion (left) and H_2_ generation rate (right) at different CH_4_ flow rates at 850 °C (N_2_ = 15 mL min^–1^). (d) CH_4_ conversion (left) and H_2_ generation rate (right) at 25 mL min^–1^ with different concentrations of CH_4_ (balanced by N_2_) at 850 °C. (e) The long-time stability measurement of CH_4_ decomposition over Ni_3_Mn–Bi liquid alloy catalysts (reaction temperature = 850 °C, N_2_ = 15 mL min^–1^, CH_4_ = 10 mL min^–1^).

The effects of methane flow rates and partial pressures on methane conversion and hydrogen evolution rate were further investigated (Figure 1c,d). Higher CH_4_ flow rate and partial pressure lead to a decrease in CH_4_ conversion while promoting the hydrogen production rate. Specifically, the CH_4_ conversion increased to 18.96% with a H_2_ generation rate of 6.78 mmol g^–1^ h^–1^ when the CH_4_ flow rate was 5 mL min^–1^ with 15 mL min^–1^ N_2_. Moreover, when the methane partial pressure was 20 vol %, CH_4_ conversion over Ni_3_Mn–Bi reached 20.5% at 850 °C. Long-term stability tests demonstrate the durability of Ni_3_Mn–Bi for 80 h under CMD reaction conditions with no deactivation being observed (Figure 1e).

Based on the relationship between reaction rate constants and temperatures, we constructed Arrhenius plots for the CH_4_ decomposition reaction over NiMn–Bi catalysts with varying Ni-to-Mn mole ratios, using H_2_ production rates at different temperatures (Figure 2a). The kinetic parameters, including the apparent activation energy and the pre-exponential factor, are provided in Figure 2b and Table S1. The results showed that the addition of Mn significantly decreased the apparent activation energy of the catalyst. Specifically, the Ni_3_Mn–Bi catalyst exhibited the lowest apparent activation energy (112.9 kJ mol^–1^), highlighting its effectiveness in lowering the energy barrier and enhancing the decomposition rate. Notably, the apparent activation energies were determined under conditions where mass transport limitations were not fully eliminated and have not completely eliminated the transport effect due to the absence of stirring in the reactor, the inherent viscosity of the molten alloy, etc. This is further evidenced by the increasing activation energies at higher CH_4_ feeding rates (10 → 15 → 20 mL/min), as shown in Figures S12–S14.

**Figure 2:**
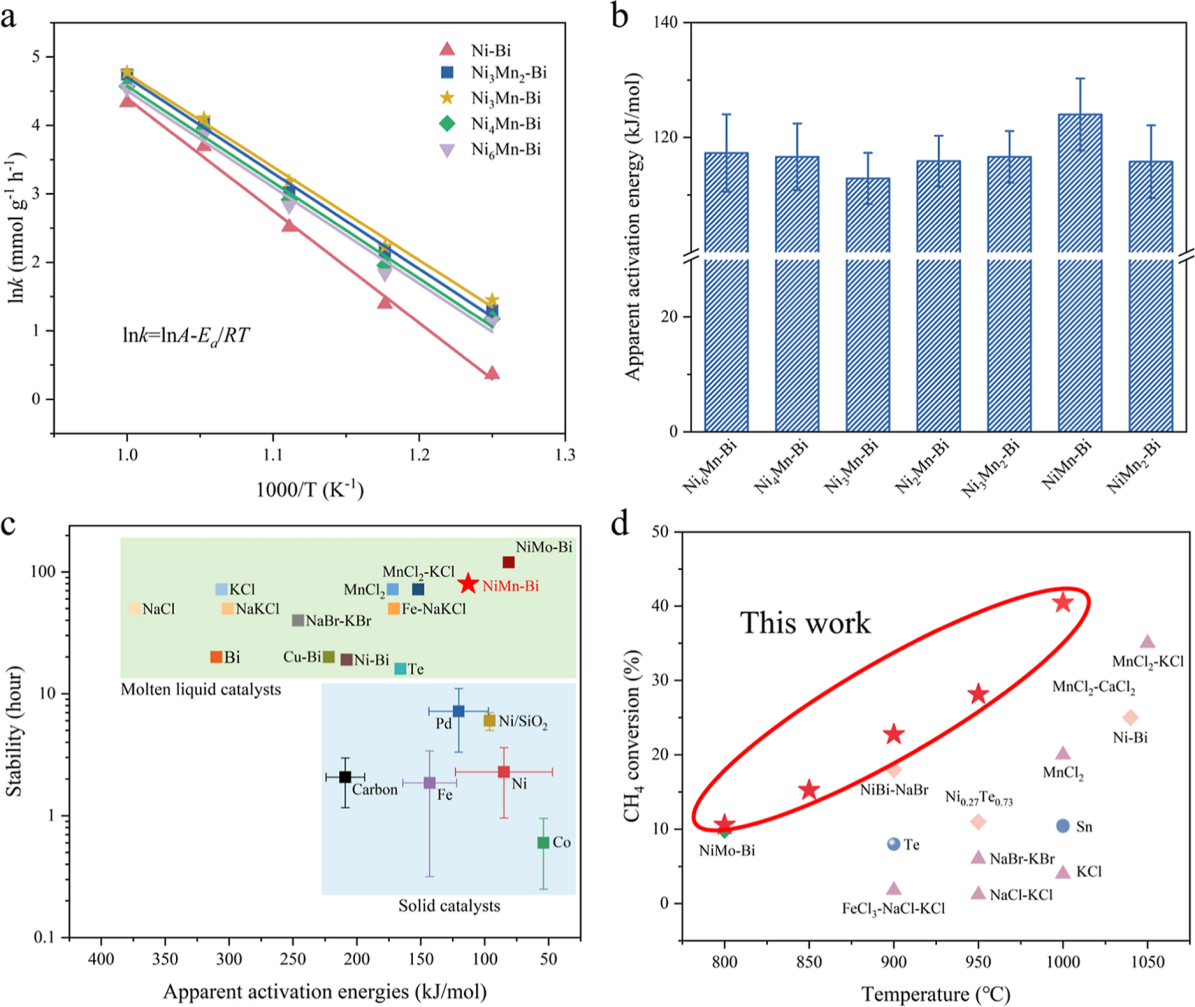
Reaction kinetics of NiMn–Bi catalysts with different Ni–Mn molar ratios. (a) Apparent activation energy of NiMn–Bi catalysts at different Ni–Mn molar ratios. (b) Apparent activation energies for CH_4_ decomposition in NiMn–Bi catalysts. (c) The stability and activity of different solid and molten liquid catalysts. (d) Methane conversion of molten liquid catalysts under different reaction conditions.

Figure 2c displays the activation energy and stability of molten liquid catalysts compared with conventional loaded transition metal catalysts. Although conventional catalysts (Ni, Co, Fe, Pt, Pd, etc.) show much lower apparent activation energies (65–96 kJ mol^–1^), they are susceptible to deactivation caused by carbon deposition and aromatic contamination and require to be improved in terms of durability and resistance to carbon buildup.^28,40,41^ In contrast, molten liquid catalysts effectively prevent deactivation by separating carbon products from the liquid-phase catalyst, ensuring long-term stability. However, the *E*_a_ of most liquid catalysts remains high, ranging from 160 to 310 kJ mol^–1^, necessitating CH_4_ activation at elevated temperatures.^42^ Compared to previously reported studies, our developed NiMn–Bi-based catalysts demonstrate competitiveness in terms of both the decomposition durability and apparent activation energy. Figure 2d compares the methane conversion at different temperatures of the molten salt and molten metal catalysts in this study to other studies. From the comparison results, the methane conversion of the NiMn–Bi catalysts prepared in this study is significantly better over the reaction temperatures of 800–1000 °C, which highlights its good catalytic performance in the methane cracking reaction.

The phase composition and crystal structures of the fresh and used catalysts were investigated by XRD (Figures 3a,b, S15, and S16). The phases of Bi (PDF#85-1329) and Bi_3_Ni (PDF#54-0537) were detected for all of the samples with different Ni-to-Mn ratios. This demonstrates that partial alloying of Bi and Ni occurred and that extra Bi exists in the metallic form. The diffraction intensity of Bi was gradually enhanced with the decrease of the Ni-to-Mn ratio, indicating the formation of larger Bi grains. In addition, the intensity of the Bi_3_Ni diffraction peaks gradually decreases as the Ni/Mn mole ratio decreases, whose intensity decreases sharply when the Ni-to-Mn ratio is lower than 1.5, owing to the generation of less Bi_3_Ni. In contrast to the Ni–Bi samples, no crystalline phase associated with the Mn component was detected when the Ni/Mn ratio was ≥1, attributed to the enhancement in the solubility of Mn in Bi as a result of the Ni–Mn interaction. However, when the Ni-to-Mn ratio decreased to 0.5, the crystalline phase of Mn_5_Ni_2_Bi_4_ (PDF#65-6044) emerged, suggesting the gradual generation of a ternary alloy with a high Mn content. This also illustrates an alteration of the interactions among metallic Ni, Mn, and Bi. Furthermore, XRD analysis comparing the phases of fresh and used catalysts, along with an investigation of the reaction temperature effect, confirmed the stability of the Ni–Bi and Ni_3_Mn–Bi catalysts.

**Figure 3:**
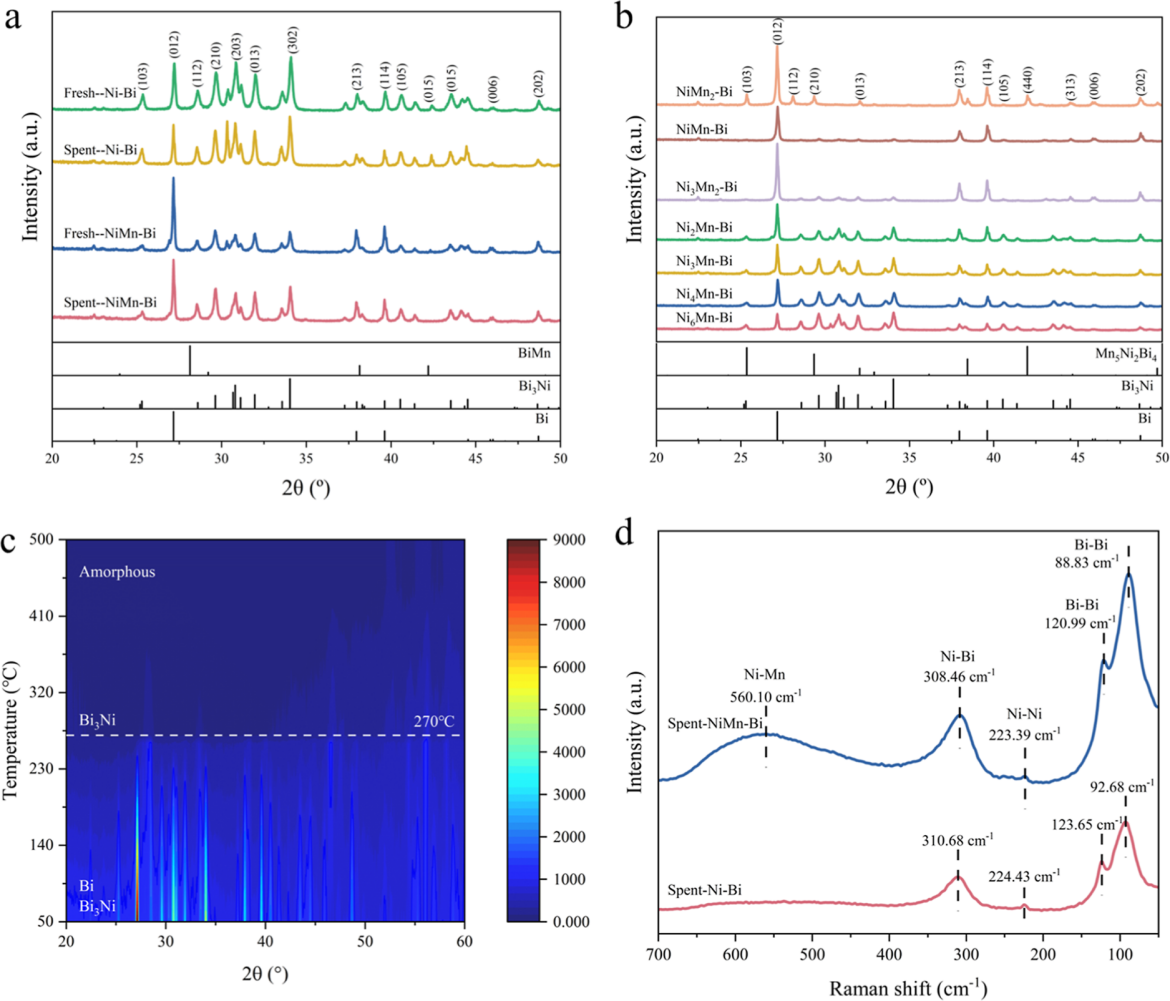
XRD patterns of (a) fresh and spent catalysts (Ni–Bi, Ni_3_Mn–Bi). (b) NiMn–Bi catalysts with different Ni–Mn molar ratios. (c) In situ high-temperature XRD patterns. (d) Raman spectra of spent Ni–Bi and Ni_3_Mn–Bi catalysts.

The phase transition behavior of Ni_3_Mn–Bi catalysts during melting was further investigated using an in situ high-temperature XRD instrument (Figure 3c). The crystalline phase of Bi disappeared at 270 °C, aligning closely with its melting point. The Bi_3_Ni phase almost disappeared when the temperature was further increased to 450 °C, indicating that both Ni and Mn predominantly remained in the liquid state at this temperature. Therefore, a uniform distribution of Ni and Mn within the molten Bi phase is realized.

The as-prepared and postreaction catalysts were characterized by Raman spectroscopy (Figures 3d and S17). Analysis of the spectra reveals that Mn doping resulted in a peak shift of Bi–Bi bonds, illustrating the interactions between Bi and Mn.^43–45^ Moreover, a broader peak that represents Ni–Mn bonds also demonstrates interactions of Ni with Mn. This phenomenon suggests that Mn modification regulates the electronic distribution of the Ni–Bi catalyst, owing to the induction of lattice distortions and the modulation of the catalyst’s vibrational frequencies.

The chemical states of the reacted liquid alloy catalysts were analyzed by using XPS (Figures 4 and S18). The Ni 2p spectra results showed that the peak for metallic Ni 2p 3/2 is located at 855.72 eV, while the peaks for Ni–Bi and Ni_3_Mn–Bi shifted to lower binding energies, corresponding to 855.34 and 855.31 eV, respectively. This shift suggests that metallic Ni gains electrons upon the introduction of both Bi and BiMn. A further decrease of 0.03 eV (0.12 eV after reaction) in Ni 2p 3/2 is observed after Mn modification, which can be attributed to changes in the surface electronic structure of Ni–Bi due to electron transfer or coordination effects.

**Figure 4:**
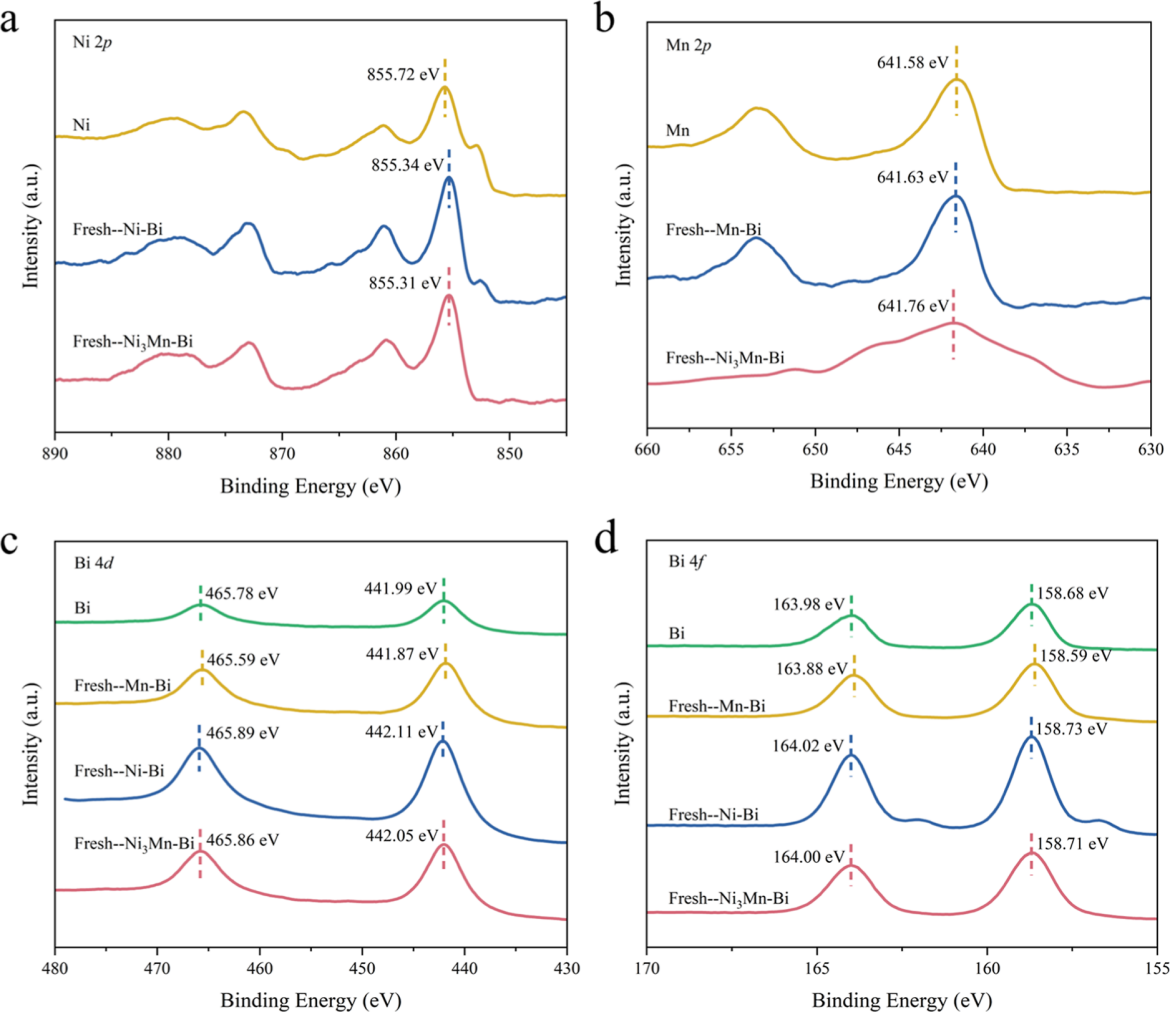
XPS analysis of the as-prepared catalysts Ni–Bi, Ni_3_Mn–Bi, and Mn–Bi. (a) Ni 2p. (b) Mn 2p. (c) Bi 4d. (d) Bi 4f.

As shown in Figure 4b, the peaks of Mn 2p 3/2 of Mn, Mn–Bi, and Ni_3_Mn–Bi appeared at 641.58, 641.63, and 641.76 eV, respectively. All these three samples exhibit higher binding energies compared to metallic Mn. This phenomenon may be attributed to surface oxidation of Mn^0^, leading to the formation of Mn^2+^ and/or Mn^3+^ species. Notably, further binding energy shifts are observed in Mn–Bi and Ni_3_Mn–Bi alloys relative to metallic Mn, indicating the formation of alloys with the occurrence of an electron transformation from Mn to Ni or Bi. The Bi 4d 5/2 and Bi 4f 7/2 spectra of Ni–Bi show peaks at 442.11 and 158.73 eV, respectively, and the corresponding peaks for Ni_3_Mn–Bi are at 442.05 and 158.71 eV, both exhibiting increasing trends compared with Bi 4d and 4f spectra (Figure 4c,d). The above results indicated the occurrence of electronic transfer from Bi and Mn to the surrounding Ni, supporting the construction of a Ni–Mn–Bi ternary alloy.

Scanning electron microscopy (SEM) images and EDS mapping results of Ni–Bi, Ni_3_Mn–Bi, Ni_3_Mo–Bi, Ni_3_Fe–Bi, Ni_3_Cr–Bi, and Ni_3_Co–Bi catalysts are provided in Figures S19–S24. All the catalysts exhibit irregular bulk morphologies with homogeneously distributed elements. SEM–EDS mapping of the N element in 2 h of reacted Ni_3_Mn–Bi indicates the formation of a small amount of nitride during methane decomposition, in agreement with the XPS results (Figures S25–S27).

The micromorphology and elemental distribution of the Ni_3_Mn–Bi samples before and after methane decomposition are shown in Figure 5. The fresh catalyst showed a circular-like bulk morphology, with Ni, Mn, and Bi elements being uniformly distributed, suggesting a well-doped state of Ni and Mn in the Bi matrix with the formation of an alloy structure. The high-resolution transmission electron microscopy (HR-TEM) image revealed distinct fringes with a crystallographic spacing of 0.308 nm corresponding to Bi_3_Ni­(112) (Figure 5c). Moreover, the diffraction rings and spots observed in the selected area electron diffraction (SAED) pattern further validate the highly ordered crystal structure of the fresh catalyst (Figure 5d). The morphology and elemental distribution of the reacted catalyst remain generally unchanged, demonstrating the excellent morphology stability of the NiMn–Bi catalyst (Figure 5e–p).

**Figure 5:**
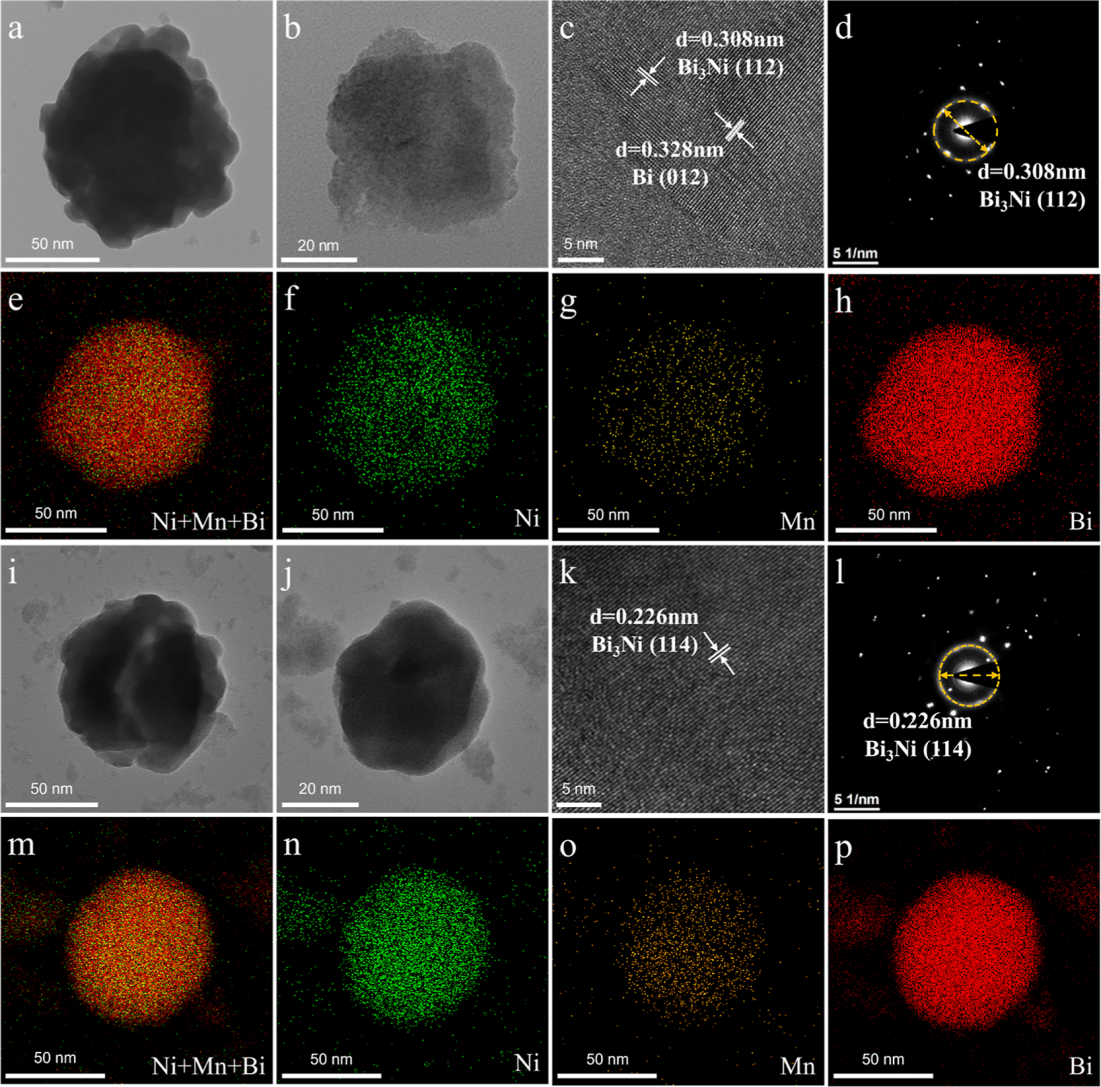
TEM images and elemental distribution analysis of Ni_3_Mn–Bi catalysts before (a–h) and after (i–p) the methane decomposition reaction. (a,b) TEM images. (c) HR-TEM images. (d) SAED pattern. (e–h) Ni, Mn, and Bi elemental distribution. (i,j) TEM images. (k) HR-TEM images. (l) SAED maps. (m–p) Ni, Mn, and Bi elemental distribution maps.

The deposited carbon was collected and characterized by N_2_ adsorption–desorption, Raman spectroscopy, SEM, TEM, and O_2_-TPO to further analyze the physiochemical properties of the carbon materials (Figures S28–S48). N_2_ adsorption results indicate that the metallic M (M = Mn, Mo, Fe, Cr, or Co) introduction shows few impacts on their BET surface area, pore diameter, and pore volume.

Molecular dynamics (MD) simulations were carried out to further clarify the physical properties, microstructural features, interfacial and surface behaviors, and chemical reaction processes of the molten alloy catalysts.^46–48^ We used the embedded atom method (EAM) potential energy model to describe the melting point characteristics of the Ni–Bi and Ni_3_Mn–Bi catalysts and simulated their transition from solid to liquid state using the NPT system to control the temperature rise (Figures S49–S51). The simulated melting point of the Ni_3_Mn–Bi alloy was 745 K, which is in agreement with the measured 723 K obtained from in situ XRD. This melting point is 135 K lower than that of Ni–Bi catalysts, revealing the reduction of melting temperature with Mn present.

The following involved concepts from MD are introduced briefly: (i) the binding energy is the energy released by atoms during the process of binding into a stable crystal; (ii) the interfacial energy is the sum of the energies per unit area when two different phases are in contact, including the surface energy and the interaction energy between atoms; (iii) the radial distribution function (RDF) is utilized to describe the density distribution of particles in space around a reference particle; (iv) the Ni–Bi interaction refers to the way in which Ni and Bi atoms are bonded together, which may involve metallic bonding, van der Waals forces, or charge transfer, etc.; and (v) the MSD is the mean of the square of the particle’s displacement over time *t* and is used to quantify the particle’s ability to migrate.

We simulated and evaluated the dissolution process of Ni_2_ and Mn_2_ dimers as well as Ni and Mn clusters by the indexes of atom distance and neighbor number. The Ni–Ni distance in the Ni–Bi catalysts periodically varied between 2 and 3 Å, indicating that the Ni–Ni distance is relatively stable in pure liquid Bi (Figure 6a).^46^ Nevertheless, the Ni–Ni distance in the Ni_3_Mn–Bi catalysts showed a significant step change after approximately 440 fs. It is hypothesized that the introduction of Mn changed the coordination environment around the Ni atoms, concurrently changing the Ni–Bi interactions. Similarly, in Figure 6b, the Mn–Mn distance in a Mn_2_ dimer was relatively stable with periodic fluctuations, while the Mn–Mn distance in Ni_3_Mn–Bi tended to sharply increase after 300 fs, indicating the accelerated dissolution with the introduction of Mn.

**Figure 6:**
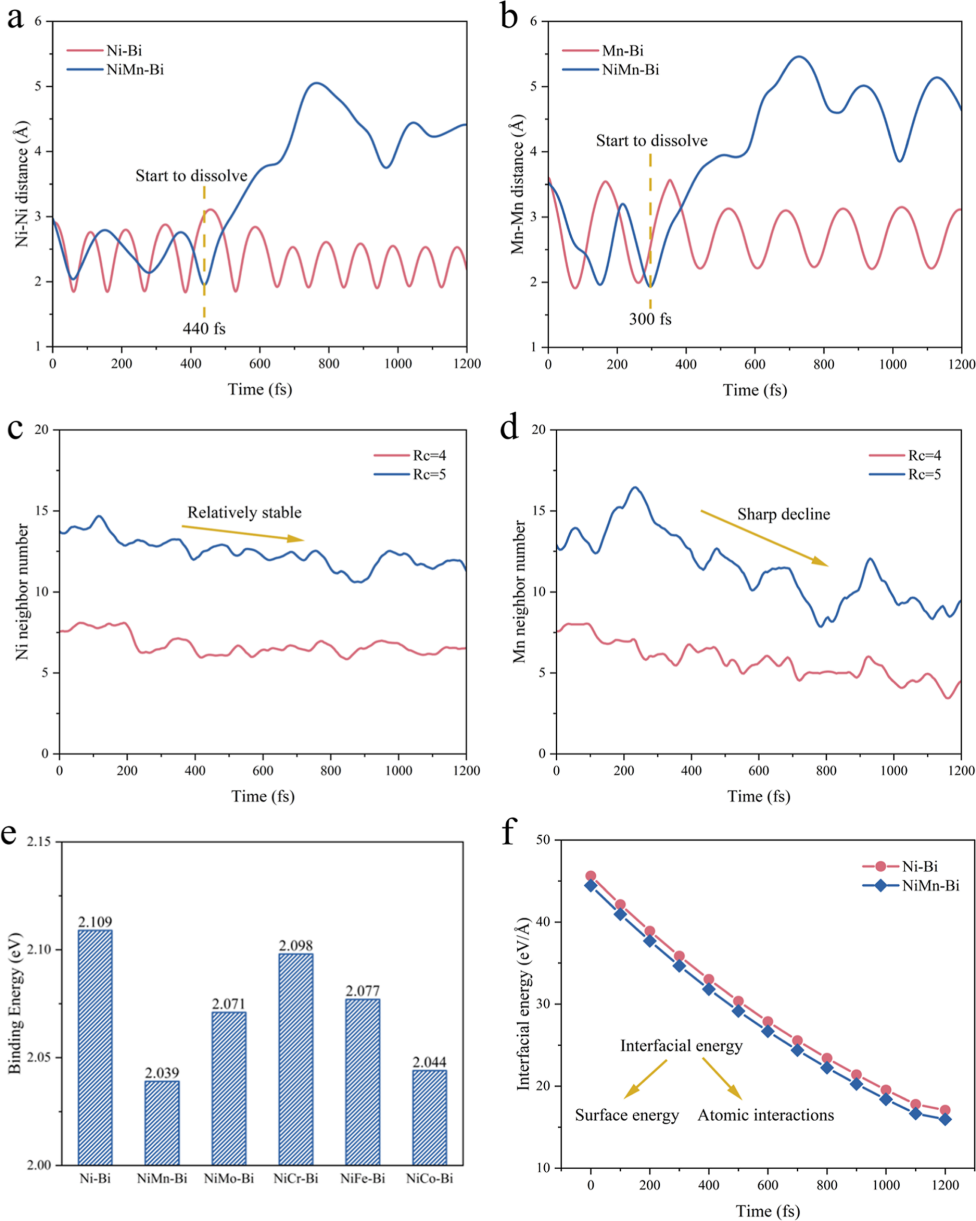
(a) The Ni–Ni distance of the Ni_2_ dimer in the Mn–Bi liquid alloy and pure liquid Bi metal. (b) The Mn–Mn distance of the Mn_2_ dimer in the Ni–Bi liquid alloy and pure liquid Bi metal. (c) The Ni neighbor number change in different cutoff radii (Rc) during the dissolution of the Ni cluster in the Mn–Bi liquid alloy. (d) The Mn neighbor number change in different Rc during the dissolution of the Mn cluster in the Ni–Bi liquid alloy. (e) Binding energy of different doping elements. (f) Interfacial energy of Ni–Bi and Ni_3_Mn–Bi catalysts.

In addition, the number of neighboring Ni atoms in Ni_3_Mn–Bi at both truncation radii (Rc = 4 and 5) exhibited similar variance over time, both showing an overall smoothing tendency (Figure 6c,d). This shows the relatively stable Ni neighbor number as a function of time. Different from the Ni neighbor number, the number of neighboring Mn atoms at larger Rc decreased faster, illustrating the effective dissolution of Mn with the surrounding atoms at Rc = 5. The results presented above strongly support the interactions of Mn with Ni and Bi.

To evaluate the effect of different doping elements on the structural stability and interfacial properties of the catalysts, binding and interfacial energies of the various Ni–Bi and Ni_3_Mn–Bi systems were carried out (Figure 6e,f). The Ni–Bi catalyst shows the highest binding energy (2.109 eV), indicating a strong bonding capability among its atoms (Ni–Ni, Ni–Bi, and Bi–Bi) and high structural stability. After the introduction of Mn atoms, the binding energy of Ni_3_Mn–Bi decreases to 2.039 eV, indicating the weakened structural stability after Mn doping.

The catalytic reaction usually occurs at the active sites on the surface or interface of the catalyst, and the interfacial energy between different phases in the alloy affects the number and distribution of active sites.^49^ The results show that the Ni–Bi interfacial energy of the Ni_3_Mn–Bi catalyst, which reflects surface energy and Ni–Mn–Bi atomic interactions, is slightly lower than that of Ni–Bi, indicating that the introduction of Mn helps to reduce the Ni–Bi interfacial energy of the catalyst. This makes the Ni_3_Mn–Bi catalyst more stable and ordered at the interface, which promotes a more uniform distribution of active sites and helps to improve the catalytic performance.

To further understand the bond-breaking and -forming processes of the catalyst, we embedded a Ni atom with a Mn atom in the Bi solution and performed molecular dynamics simulations at 1500 K. Initially, the Ni and the Bi atoms were neatly aligned, and the Ni–Bi bond was in a stable state. After 800 fs, the Ni atom gradually dissociates from the surroundings of the Bi atom (Figure S52). Notably, the introduction of Mn atoms made it easier for Ni atoms to dissociate from the encirclement of Bi atoms, which takes only 500 fs. Therefore, the formed Ni–Mn bond weakened the interactions between Ni and Bi atoms, which is seen as the catalytically active site of CMD, and Bi acts as a promoter while providing the necessary molten environment (Figure S53). The bond breaking and forming of Ni_3_M–Bi (M = Co, Fe, Mo, Cr, Cu, W) catalysts within 1100 fs were also simulated, testifying to the promoting effect of M addition (Figures S54–S59).

The radial distribution function (RDF) of the Ni–Bi and Ni_3_Mn–Bi catalysts was further simulated to gain insight into their interatomic interactions and structural properties (Figures S60 and S61). The Ni–Bi catalyst showed a much higher distributed density of the Ni–Ni bond within the distance range 2–3 Å, supporting strong interactions between Ni and Ni with a compact structure. In contrast, the distributions of Ni–Bi and Bi–Bi are more dispersed with weak interactions. The introduction of Mn enhanced the Ni–Ni and Ni–Mn interactions, implying better solubility of Mn atoms in Ni with fewer Mn clusters forming.

The Ni–Bi interactions in Ni–Bi and Ni_3_Mn–Bi catalysts with times were simulated (Figures S62 and S63). Results demonstrate the structure evolution with time, visually reflecting the diffusion and dynamic behaviors of these two catalyst systems. Both Ni–Bi and Ni_3_Mn–Bi gradually transform from an ordered structure to a chaotic state. Specifically, the results of the calculations confirm the lower Ni–Bi interaction of Ni_3_Mn–Bi. Introduction of Mn changes the spatial arrangement between Ni and Bi, and the formation of Ni–Mn bonds weakens the interaction between Ni and Bi. This makes it easier for Ni atoms to detach from the surrounding Bi atoms, thus enriching the active sites of the catalysts and facilitating enhanced CMD performance. We consider that the metallic interaction would be a potential descriptor that affects CMD activity.

Subsequently, we analyzed the dissociation process of CH_4_ at 1500 K with applied Ni–Bi and Ni_3_Mn–Bi catalysts and statistically calculated the number of H atoms around the CH_4_ molecule (Figure 7a,b). The cleavage of the CH_4_ molecule under the action of Ni–Bi catalysts was more difficult, the H atoms were hard to separate from CH_4_, and the primary dissociation time of the Ni–Bi system was approximately 280 fs, while for Ni_3_Mn–Bi, the CH_4_ dehydrogenation occurs at 120 fs. Moreover, as the reaction proceeds, the number of hydrogen atoms in Ni_3_Mn–Bi decreases faster than that in Ni–Bi, supporting the activity enhancement through the addition of Mn.

**Figure 7:**
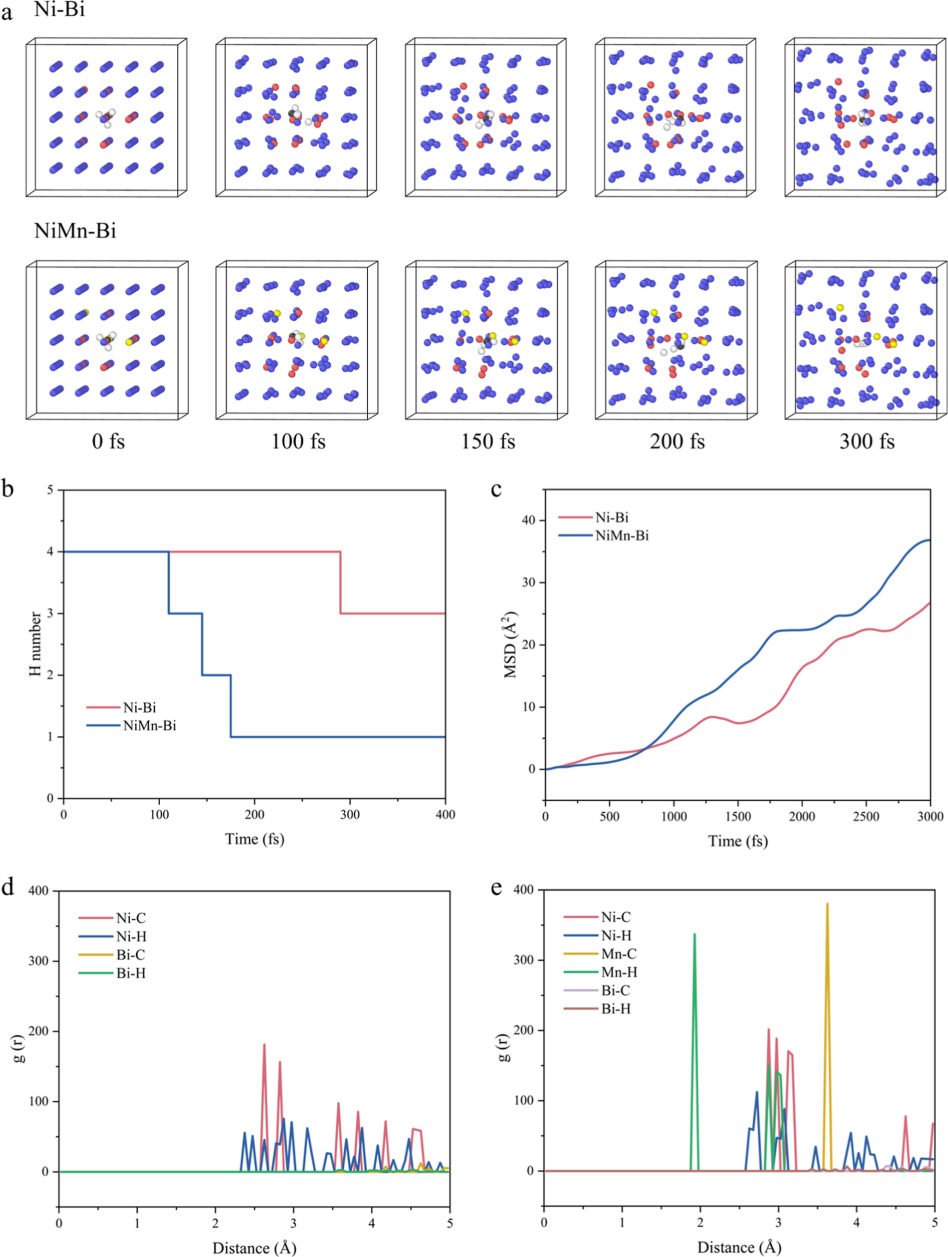
Molecular dynamics simulations. (a) Snaps of CH_4_ in the liquid alloy of Ni–Bi and Ni_3_Mn–Bi. (b) H atomic number around a C atom as a function of time. (c) Mean square displacement (MSD) of CH_4_ molecules in Ni–Bi and Ni_3_Mn–Bi liquid alloys. RDF in the methane decomposition reaction: (d) Ni–Bi catalysts and (e) Ni_3_Mn–Bi catalysts.

Furthermore, we calculated the mean square displacement (MSD) to analyze the motion trajectories of CH_4_ molecules in the reaction system and their diffusion properties (Figure 7c). The CH_4_ molecules in Ni_3_Mn–Bi show higher MSD values, which suggests their excellent mobility and diffusivity in methane dissociation, thereby significantly enhancing the diffusivity of a CH_4_ molecule and accelerating the hydrogenation rate.

RDF simulations of CH_4_ molecules on the Ni–Bi and Ni_3_Mn–Bi catalysts were conducted to analyze the activation and formation of different chemical bonds within a specific distance of Ni, Mn, and Bi atoms, thereby assessing the catalytically active sites of the catalysts (Figures 7d,e and S64–S66). Ni–C and Ni–H bonds were observed as the main structure, demonstrating that Ni is the main active phase of the Ni–Bi alloy. For Ni_3_Mn–Bi, the observed Ni–C and Mn–C bonds indicate the dual active sites of the Ni_3_Mn–Bi catalyst in methane decomposition.

The relationships between the H_2_ generation rate obtained from the experimental measurements and the potential descriptors obtained from the MD simulation are summarized in Figure 8 (Figures S67 and S68). By examining the evolution of the hydrogen production rate as functions of binding energy, interfacial energy, Ni–Bi interaction, and MSD, the R^2^ values of the curves fitted are 0.947, 0.981, 0.940, and 0.931, respectively. Therefore, the interfacial energy plays a crucial role in the description of the catalytic activity of a Ni–Bi-based ternary alloy catalyst, showing strong predictive ability and providing reliable theoretical guidance for subsequent catalyst exploitation. To further validate the accuracy of interfacial energy as a predictor of hydrogen production performance, the interfacial energy of a new molten-alloy catalyst, Ni_3_Cu–Bi, was calculated while evaluating its experimental hydrogen generation performance. The results are consistent with our previously proposed pattern, confirming that interfacial energy is a reliable descriptor for performance prediction (Figure S69).

**Figure 8:**
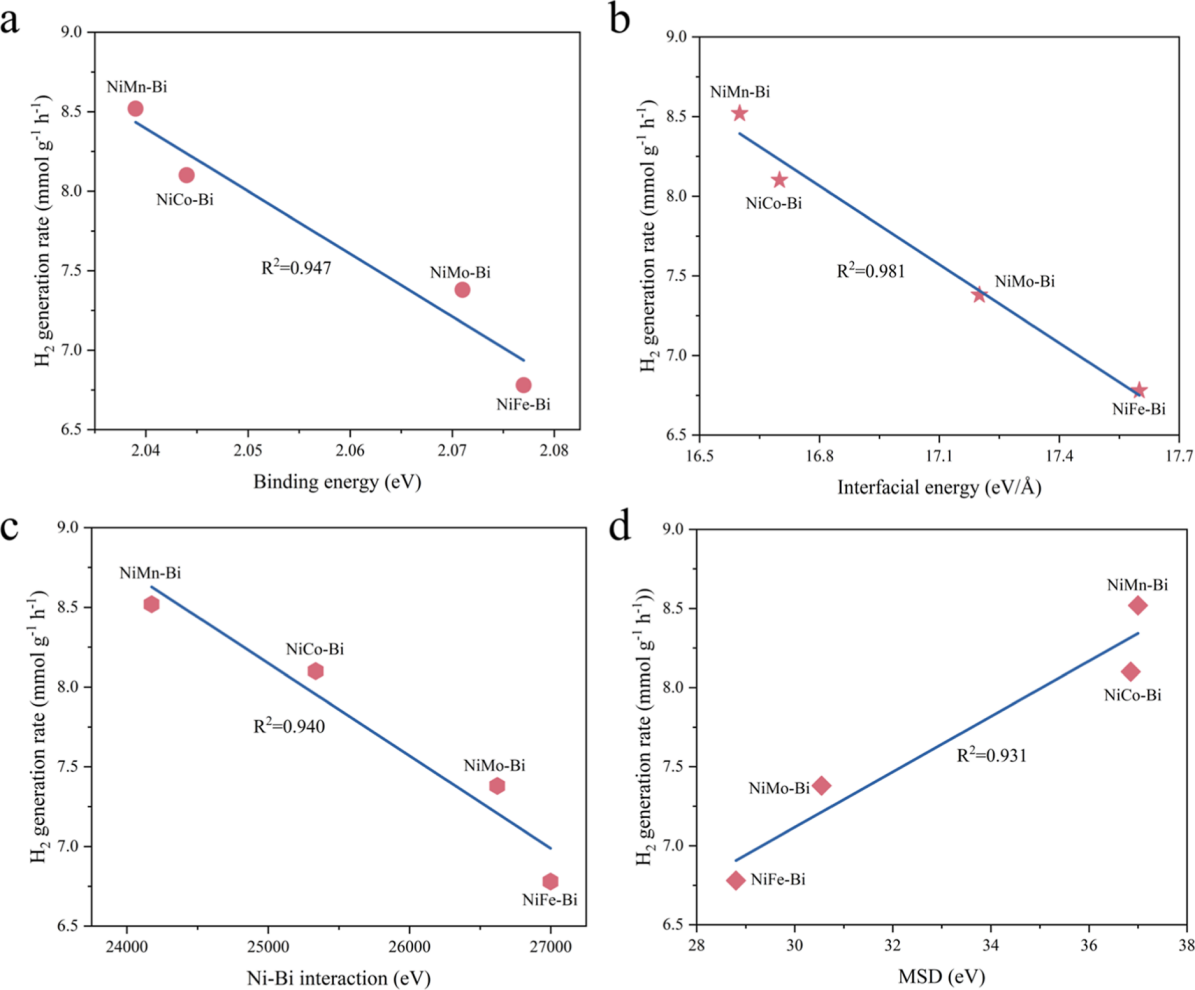
Relationship between different descriptors and decomposition performance of methane: (a) binding energy, (b) interfacial energy, (c) Ni–Bi interaction, and d) MSD.

## 4 Conclusions

In summary, we designed a series of NiMn–Bi-based liquid alloy catalysts, with the aim of developing catalysts that perform at high activity with robust durability in CMD. Experimental results revealed that the Ni_3_Mn–Bi catalyst performed relatively well, with a methane conversion of 15.3% and hydrogen productivity of 9.30 mmol g^–1^ h^–1^ at 850 °C, and remained active after 80 h of CMD at this level. These results demonstrate that the introduction of Mn modulates the electronic structure and accelerates the dehydrogenation rate, thereby considerably decreasing the decomposition temperature required. MD simulation of binding energy, interfacial energy, Ni–Bi interactions, and MSD sufficiently illuminates the superior characteristics of Ni_3_Mn–Bi, which originate from the modulation of metallic interactions, thereby upgrading the electronic structure and active site distribution of the catalyst. We consider that the interfacial energy, as a comprehensive descriptor of surface energy and interatomic interactions, shows the strongest correlation with the catalytic performance among the parameters evaluated. Looking ahead, further advancements can be made by (i) establishing effective descriptors for the prediction and screening of potential molten-alloy catalysts; (ii) rationally tuning catalysts by modulating their interfacial energy; and (iii) developing a novel reactor that extends the methane residence time, thereby improving methane conversion efficiency.

## Supplementary Material



## Notes

Experimental results; molten alloy catalyst characterizations; carbon microtube analysis; and MD simulations (PDF)
