# Supplementary material for: Modulating the Interfacial Energy of Ni–Bi Molten Alloys for Enhanced Methane Decomposition to Hydrogen
Source: ACS Catal. 2025 Oct 3;15(20):17333–46. doi: 10.1021/acscatal.5c02867 (PMC12538552; doi:10.1021/acscatal.5c02867)
Supplement: Supplementary file 1 [file cs5c02867_si_001.pdf]

Supporting Information

Modulating the Interfacial Energy of Ni-Bi Molten Alloy for Enhanced Methane Decomposition to Hydrogen

Zhao Sun[1,2,*], Bin Wang[1], Nicholas F. Dummer[2], Haifeng Qi[2], Louise R. Smith[2], Zhiqiang Sun[1,*], Graham J. Hutchings[2,*]

[1]Hunan Engineering Research Centre of Clean and Low-Carbon Energy Technology, School of Energy Science and Engineering, Central South University, Changsha 410083, China.

[2]Max Planck–Cardiff Centre on the Fundamentals of Heterogeneous Catalysis FUNCAT, Cardiff Catalysis Institute, School of Chemistry, Cardiff University, Cardiff CF24 4HQ, United Kingdom.

*Corresponding authors. E-mails:

zhaosun@csu.edu.cn (Z. Sun)

zqsun@csu.edu.cn (Z.Q. Sun)

Hutch@cardiff.ac.uk (G.J. Hutchings)

Chart list:

Figure S1. Schematic diagram of bubble column reactor system.

Figure S2. CH_4 conversion (left) and H_2 generation rate (right) of liquid alloy catalysts with different doping elements NiM-Bi (M = W, Mo, Mn, Co, Fe, Cr, Cu). Reaction temperature = 800 °C.

Figure S3. CH_4 conversion (left) and H_2 generation rate (right) of liquid alloy catalysts with different doping elements NiM-Bi (M = W, Mo, Mn, Co, Fe, Cr, Cu). Reaction temperature = 850 °C.

Figure S4. CH_4 conversion (left) and H_2 generation rate (right) of liquid alloy catalysts with different doping elements NiM-Bi (M = W, Mo, Mn, Co, Fe, Cr, Cu). Reaction temperature = 900 °C.

Figure S5. CH_4 conversion (left) and H_2 generation rate (right) of liquid alloy catalysts with different doping elements NiM-Bi (M = W, Mo, Mn, Co, Fe, Cr, Cu). Reaction temperature = 950 °C.

Figure S6. CH_4 conversion (left) and H_2 generation rate (right) of liquid alloy catalysts with different doping elements NiM-Bi (M = W, Mo, Mn, Co, Fe, Cr, Cu). Reaction temperature = 1000 °C.

Figure S7. CH_4 conversion (left) and H_2 generation rate (right) of liquid alloy catalysts with different Ni-Bi molar ratio. The molar ratio of Ni-Mn is 3:1, Reaction temperature = 800 °C.

Figure S8. CH_4 conversion (left) and H_2 generation rate (right) of liquid alloy catalysts with different Ni-Bi molar ratio. The molar ratio of Ni-Mn is 3:1, Reaction temperature = 850 °C.

Figure S9. CH_4 conversion (left) and H_2 generation rate (right) of liquid alloy catalysts with different Ni-Bi molar ratio. The molar ratio of Ni-Mn is 3:1, Reaction temperature = 900 °C.

Figure S10. CH_4 conversion (left) and H_2 generation rate (right) of liquid alloy catalysts with different Ni-Bi molar ratio. The molar ratio of Ni-Mn is 3:1, Reaction temperature = 950 °C.

Figure S11. CH_4 conversion (left) and H_2 generation rate (right) of liquid alloy catalysts with different Ni-Bi molar ratio. The molar ratio of Ni-Mn is 3:1, Reaction temperature = 1000 °C.

Figure S12. Apparent activation energy of the Ni_3Mn-Bi alloy catalyst under a CH_4 flow rate at 15 mL/min and an N_2 flow rate at 22.5 mL/min.

Figure S13. Apparent activation energy of the Ni_3Mn-Bi alloy catalyst under a CH_4 flow rate at 20 mL/min and an N_2 flow rate at 30 mL/min.

Figure S14. Comparisons of the apparent activation energy under different CH_4 and N_2 feeding rate.

Figure S15. XRD patterns of different doping elements NiM-Bi (M = Mn, Mo, Cr, Fe, Co).

Figure S16. XRD patterns of Ni_3Mn-Bi catalysts with different reaction temperatures.

Figure S17. Raman spectra of fresh Ni-Bi, NiMn-Bi catalysts.

Figure S18. XPS analysis of the spent catalysts Ni-Bi, Ni_3Mn-Bi and Mn-Bi catalysts. a) Ni 2*p*. b) Mn 2*p*. c) Bi 4*d*. d) Bi 4*f*.

Figure S19. SEM characterization of Ni-Bi catalysts. a-d) Morphology and particle structure of catalysts at different magnifications. e) Energy dispersive X-ray spectroscopy (EDS). f) EDS mapping of Ni-Bi. g) EDS mapping of the element Ni. h) EDS mapping of the element Bi.

Figure S20. SEM characterization of Ni_3Mn-Bi catalysts. a-d) Morphology and particle structure of catalysts at different magnifications. e) EDS mapping of Ni_3Mn-Bi. f) EDS mapping of the element Ni. g) EDS mapping of the element Mn. h) EDS mapping of the element Bi.

Figure S21. SEM characterization of Ni_3Mo-Bi catalysts. a-c) Morphology and particle structure of Ni_3Mo-Bi catalysts at different magnifications. d) EDS mapping of Ni_3Mo-Bi catalysts. d1-d3) EDS mapping of Ni, Mo, Bi element.

Figure S22. SEM characterization of Ni_3Fe-Bi catalysts. a-c) Morphology and particle structure of Ni_3Fe-Bi catalysts at different magnifications. d) EDS mapping of Ni_3Fe-Bi catalysts. d1-d3) EDS mapping of Ni, Fe, Bi element.

Figure S23. SEM characterization of Ni_3Cr-Bi catalysts. a-c) Morphology and particle structure of Ni_3Cr-Bi catalysts at different magnifications. d) EDS mapping of Ni_3Cr-Bi catalysts. d1-d3) EDS mapping of Ni, Cr, Bi element.

Figure S24. SEM characterization of Ni_3Co-Bi catalysts. a-c) Morphology and particle structure of Ni_3Co-Bi catalysts at different magnifications. d) EDS mapping of Ni_3Co-Bi catalysts. d1-d3) EDS mapping of Ni, Co, Bi element.

Figure S25. SEM characterization of Ni_3Mn-Bi catalysts. a-b) Morphology and particle structure of Ni_3Mn-Bi catalysts. c) EDS mapping of Ni_3Mn-Bi catalysts. d) Elemental analysis. e-h) EDS mapping of Ni, Mn, Bi, and N element.

Figure S26. SEM characterization of Ni_3Mn-Bi catalysts. a-b) Morphology and particle structure of Ni_3Mn-Bi catalysts. c) EDS mapping of Ni_3Mn-Bi catalysts. d) Elemental analysis. e-h) EDS mapping of Ni, Mn, Bi, and N element.

Figure S27. XPS result of N1*s* for the 2 h reacted Ni_3Mn-Bi alloy catalyst.

Figure S28. N_2 adsorption-desorption isothermal curves of catalysts after methane decomposition reaction. a) NiM-Bi (M =Mn, Mo, Fe, Cr, Co) catalysts with different doping elements. b) NiMn-Bi catalysts with different Ni-Mn molar ratios.

Figure S29. Raman spectra of a) Solid carbon of Ni-Bi and NiM-Bi (M = Mn, Mo, Cr, Fe, Co) catalysts with different doping elements. b) I_D/I_G values of different doping elements.

Figure S30. Raman spectra of a) Different reaction times of Ni_3Mn-Bi catalyst. b) I_D/I_G values of different reaction times.

Figure S31. Raman spectra of catalysts with a) Different Ni-Bi molar ratios. b) I_D/I_G values of catalysts with different Ni-Bi molar ratios.

Figure S32. Raman spectra and TEM characterization of solid carbon under different reaction conditions. a) Different Ni-Mn molar ratios (850 °C). b) I_D/I_G values of different Ni-Mn molar ratios. c) Different reaction temperatures (Ni_3Mn-Bi). d) I_D/I_G values of different reaction temperatures. e) TEM images. f) HR-TEM images. g) SAED pattern. h-k) C, Ni, Mn, Bi elemental distribution. l) TEM images. m) HR-TEM images. n) SAED maps. o-r) C, Ni, Mn, Bi elemental distribution maps.

Figure S33. SEM results a-d) SEM images of solid carbon derived from CMD at 850 °C for 2 h and catalyzed by Ni_3Mn-Bi. e) EDS mapping results of (e) C; (f) Bi; (g) Ni; and (h) Mn elements.

Figure S34. SEM results a-c) SEM images of carbon microtubes derived from CMD at 850 °C for 2 h and catalyzed by Ni_3Mn-Bi. d) EDS mapping results of (e) C; (f) Bi; (h) Ni; and (i) Mn elements. g) Energy dispersive X-ray spectroscopy (EDS).

Figure S35. SEM images of solid carbon derived from CMD at 850 °C for 2 h and catalyzed by Ni_3Mn-Bi.

Figure S36. SEM results a-c) SEM images of solid carbon derived from CMD at 850 °C for 2 h and catalyzed by Ni_3Mn-Bi. d) EDS mapping results of (e) C; (f) Bi; (h) Ni; and (i) Mn elements. g) Energy dispersive X-ray spectroscopy (EDS).

Figure S37. SEM images of solid carbon derived from CMD at 850 °C for 2 h and catalyzed by Ni_3Mn-Bi. a) EDS mapping results of (b) C; (c) Bi; (e) Ni; and (f) Mn elements. d) Energy dispersive X-ray spectroscopy (EDS).

Figure S38. SEM results a-d) SEM images of solid carbon derived from CMD at 850 °C for 2 h and catalyzed by Ni-Bi. e) EDS mapping results of (f) C; (g) Ni; and (h) Bi elements.

Figure S39. SEM results a-d) SEM images of carbon microtubes derived from CMD at 850 °C for 2 h and catalyzed by Ni-Bi. e) EDS mapping results of (f) C; (g) Ni; and (h) Bi elements.

Figure S40. SEM results a-c) SEM images of solid carbon derived from CMD at 850 °C for 2 h and catalyzed by $Ni_{0.21}Mn_{0.07}$-$Bi_{0.79}$. d) EDS mapping results of (e) C; (f) Bi; (h) Ni; and (i) Mn elements. g) Energy dispersive X-ray spectroscopy (EDS).

Figure S41. SEM results a-c) SEM images of carbon microtubes derived from CMD at 850 °C for 2 h and catalyzed by $Ni_{0.21}Mn_{0.07}$-$Bi_{0.79}$. d) EDS mapping results of (e) C; (f) Bi; (h) Ni; and (i) Mn elements. g) Energy dispersive X-ray spectroscopy (EDS).

Figure S42. SEM results a-d) SEM images of solid carbon derived from CMD at 850 °C for 2 h and catalyzed by $Ni_{0.33}Mn_{0.11}$-$Bi_{0.67}$. EDS mapping results of e) C; (f) Ni; (g) Mn and (h) Bi elements.

Figure S43. SEM results a-d) SEM images of carbon microtubes derived from CMD at 850 °C for 2 h and catalyzed by $Ni_{0.33}Mn_{0.11}$-$Bi_{0.67}$. EDS mapping results of e) C; (f) Ni; (g) Mn and (h) Bi elements.

Figure S44. O_2-TPO curves of Ni_3Mn-Bi catalysts.

Figure S45. O_2-TPO curves of Ni_3Mo-Bi catalysts.

Figure S46. O_2-TPO curves of Ni_3Cr-Bi catalysts.

Figure S47. O_2-TPO curves of Ni_3Fe-Bi catalysts.

Figure S48. O_2-TPO curves of Ni_3Co-Bi catalysts.

Figure S49. Temperature-potential energy curves modeling the melting point of catalysts. a) Ni-Bi catalyst. b) Ni_3Mn-Bi catalyst.

Figure S50. Molecular dynamics simulation study: melting process of Ni-Bi catalysts in the temperature range of 300-1000 K.

Figure S51. Molecular dynamics simulation study: melting process of Ni_3Mn-Bi catalysts in the temperature range of 300-1000 K.

Figure S52. Simulation of bond breaking and bonding of Ni-Bi catalysts at different times at 1500 K.

Figure S53. Simulation of bond breaking and bonding of Ni_3Mn-Bi catalysts at different times at 1500 K.

Figure S54. Simulation of bond breaking and bonding of Ni_3Co-Bi catalysts at different times at 1500 K.

Figure S55. Simulation of bond breaking and bonding of Ni_3Fe-Bi catalysts at different times at 1500 K.

Figure S56. Simulation of bond breaking and bonding of Ni_3Mo-Bi catalysts at different times at 1500 K.

Figure S57. Simulation of bond breaking and bonding of Ni_3Cr-Bi catalysts at different times at 1500 K.

Figure S58. Simulation of bond breaking and bonding of Ni_3Cu-Bi catalysts at different times at 1500 K.

Figure S59. Simulation of bond breaking and bonding of Ni_3W-Bi catalysts at different

times at 1500 K.

Figure S60. Molecular dynamics simulation of radial distribution function versus distance for catalysts. a) Ni-Bi catalysts. b) Ni_3Mn-Bi catalysts.

Figure S61. Molecular dynamics simulation of radial distribution function versus distance for different doping elements catalysts. a) Ni_3Co-Bi catalysts. b) Ni_3Fe-Bi catalysts. c) Ni_3Cr-Bi catalysts. d) Ni_3Mo-Bi catalysts. e) Ni_3Cu-Bi catalysts. f) Ni_3W-Bi catalysts.

Figure S62. Molecular dynamics simulation of the evolution of Ni-Bi interactions in Ni-Bi and Ni_3Mn-Bi catalysts at 1500 K. Different colors represent different atomic species, red for Ni atoms, blue and yellow for Bi and doped Mn atoms, respectively.

Figure S63. Initial modeling for molecular dynamics simulations of the interaction between Ni-Bi. a) Ni-Bi catalysts. b) Ni_3Mn-Bi catalysts. c) Evolution of the Ni-Bi interaction in Ni-Bi and NiM-Bi（M=Mo，Fe, Co, Mn） catalysts as a function of simulation duration.

Figure S64. Simulation of RDF in methane decomposition reaction: a) CH_4 molecule in Ni-Bi catalyst. b) CH_4 molecule in NiMn-Bi catalyst.

Figure S65. Simulation of RDF in methane decomposition reaction: CH_4 molecule in a) Ni_3Co-Bi catalysts. b) Ni_3Fe-Bi catalysts. c) Ni_3Cr-Bi catalysts. d) Ni_3Mo-Bi catalysts. e) Ni_3Cu-Bi catalysts. f) Ni_3W-Bi catalysts.

Figure S66. Simulation of RDF in methane decomposition reaction: a) Ni_3Co-Bi catalysts. b) Ni_3Fe-Bi catalysts. c) Ni_3Cr-Bi catalysts. d) Ni_3Mo-Bi catalysts. e) Ni_3Cu-Bi catalysts. f) Ni_3W-Bi catalysts.

Figure S67. Interfacial energy of Ni-Bi and NiM-Bi (M=Fe, Mo, Co, Mn) catalysts.

Figure S68. Mean square displacement (MSD) of CH_4 molecules in Ni-Bi and NiM-Bi (M=Fe, Mo, Co, Mn) liquid alloys.

Figure S69. Verification of interfacial energy as a reliable descriptor for performance prediction.

Table S1. Reaction kinetics for CH_4 decomposition in NiMn-Bi.

Table S2. Methane conversion of catalyst under different reaction conditions.

Table S3. Structural properties of NiM-Bi (M = Mn, Mo, Fe, Cr, Co) catalysts with different doping elements.

Table S4. Structural properties of NiMn-Bi catalysts with different Ni-Mn molar ratios.

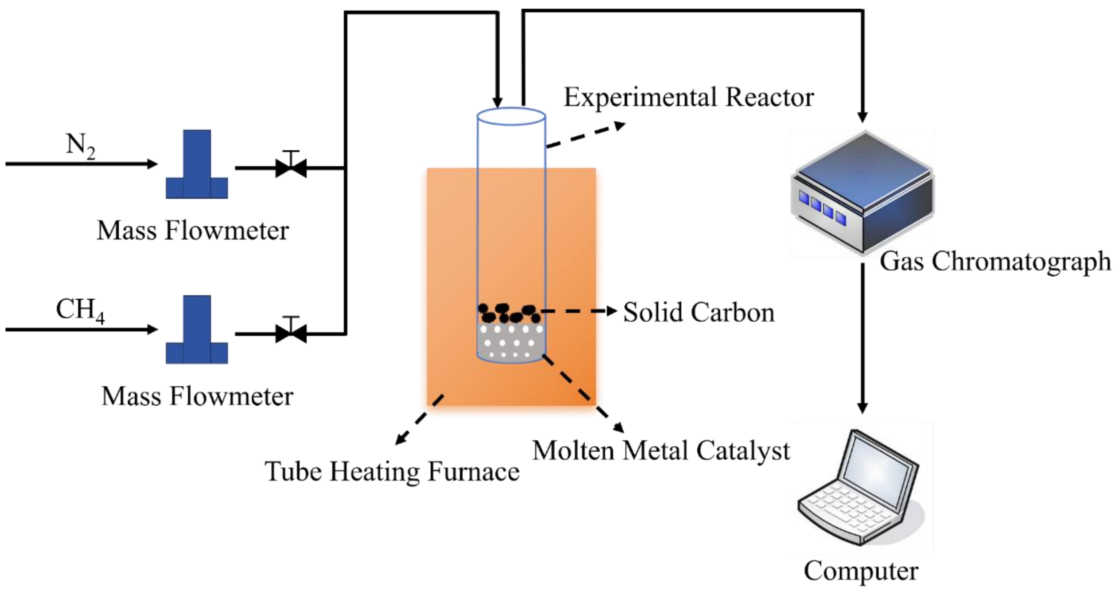

Figure S1. Schematic diagram of bubble column reactor system.

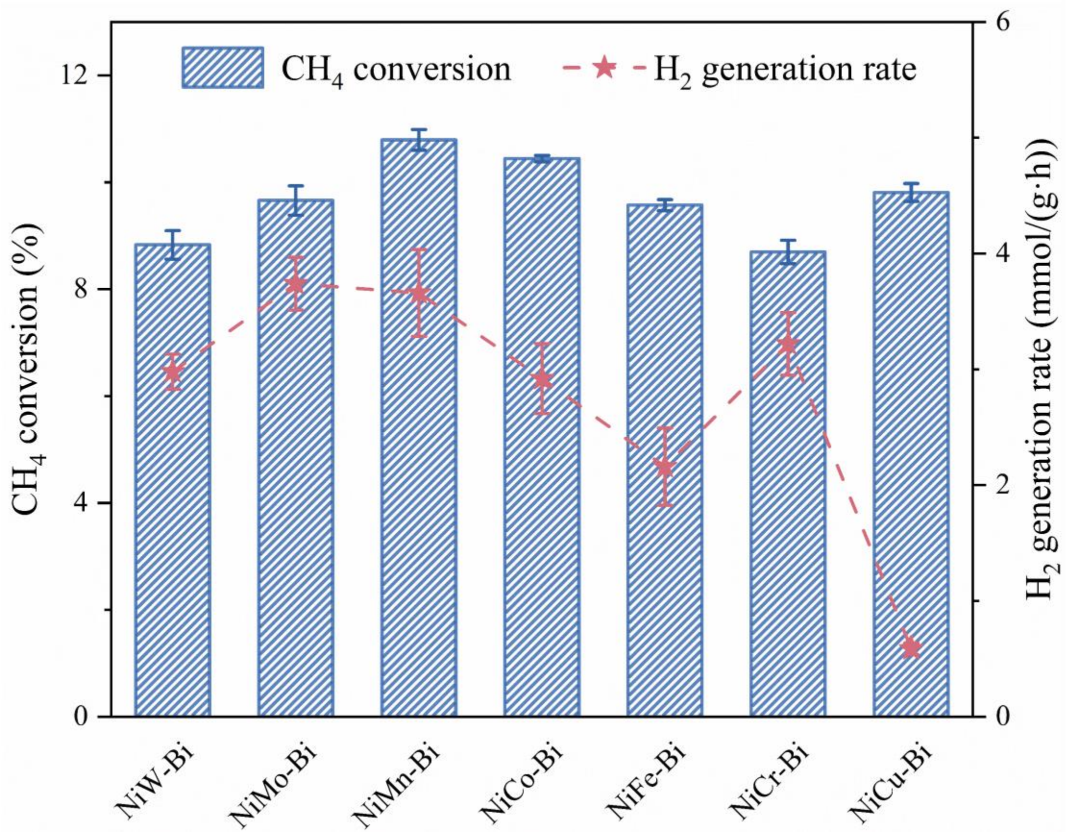

Figure S2. CH_4 conversion (left) and H_2 generation rate (right) of liquid alloy catalysts with different doping elements NiM-Bi (M = W, Mo, Mn, Co, Fe, Cr, Cu). Reaction temperature = 800 ℃.

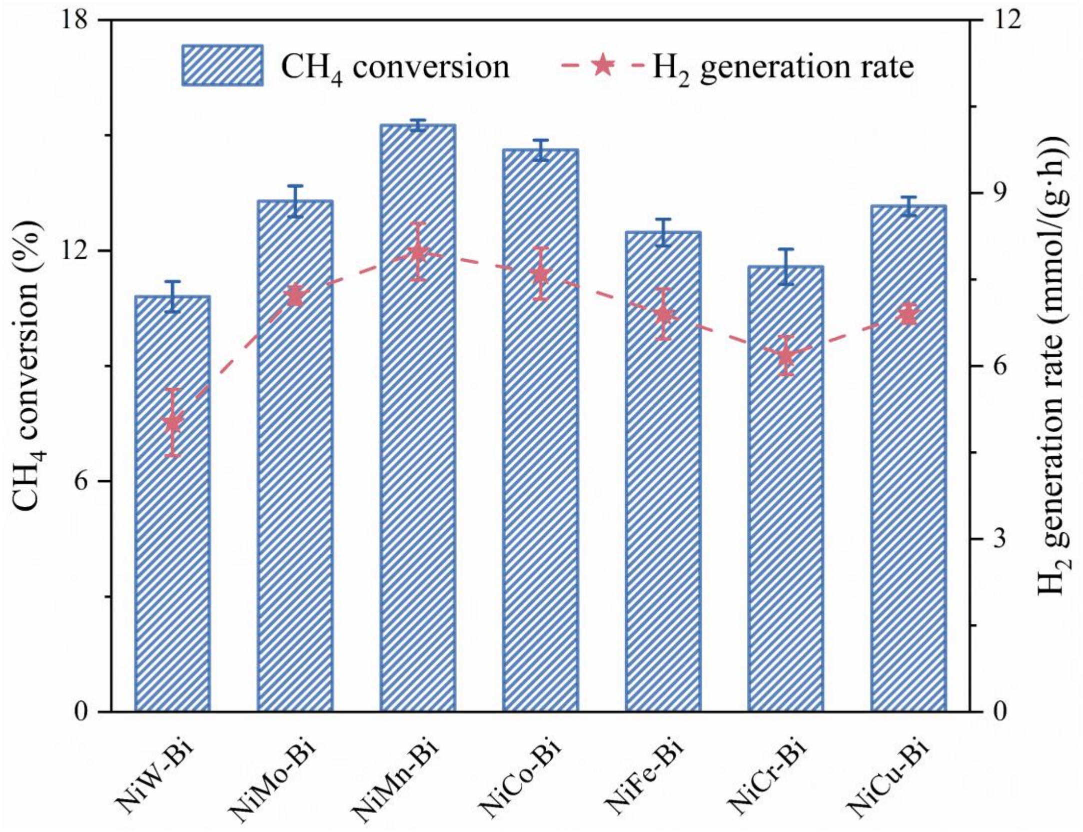

Figure S3. CH_4 conversion (left) and H_2 generation rate (right) of liquid alloy catalysts with different doping elements NiM-Bi (M = W, Mo, Mn, Co, Fe, Cr, Cu). Reaction temperature = 850 °C.

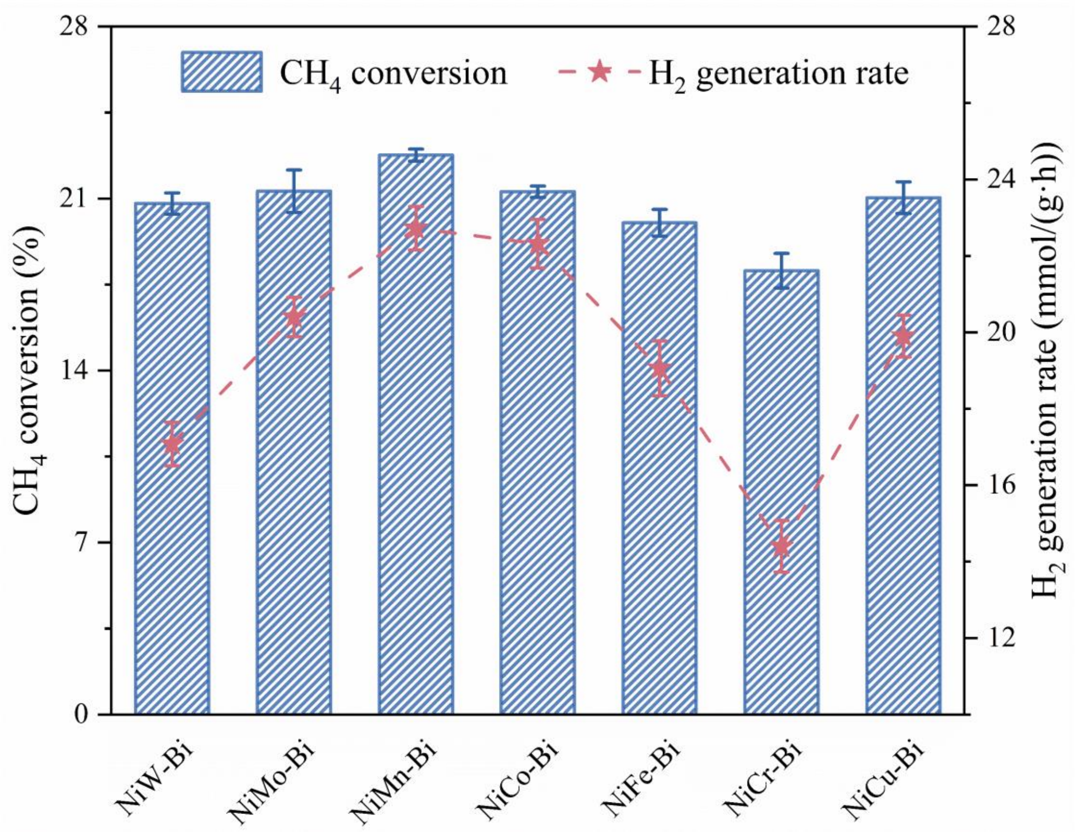

Figure S4. CH_4 conversion (left) and H_2 generation rate (right) of liquid alloy catalysts with different doping elements NiM-Bi (M = W, Mo, Mn, Co, Fe, Cr, Cu). Reaction temperature = 900 °C.

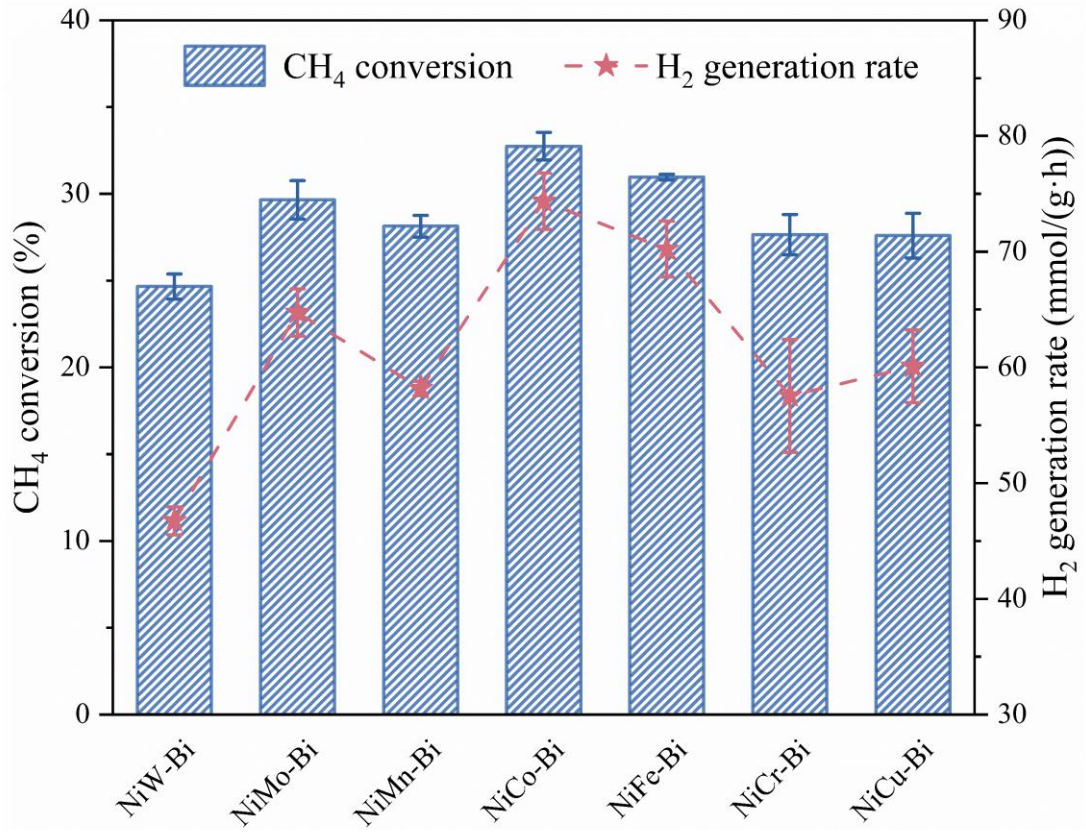

Figure S5. CH_4 conversion (left) and H_2 generation rate (right) of liquid alloy catalysts with different doping elements NiM-Bi (M = W, Mo, Mn, Co, Fe, Cr, Cu). Reaction temperature = 950 °C.

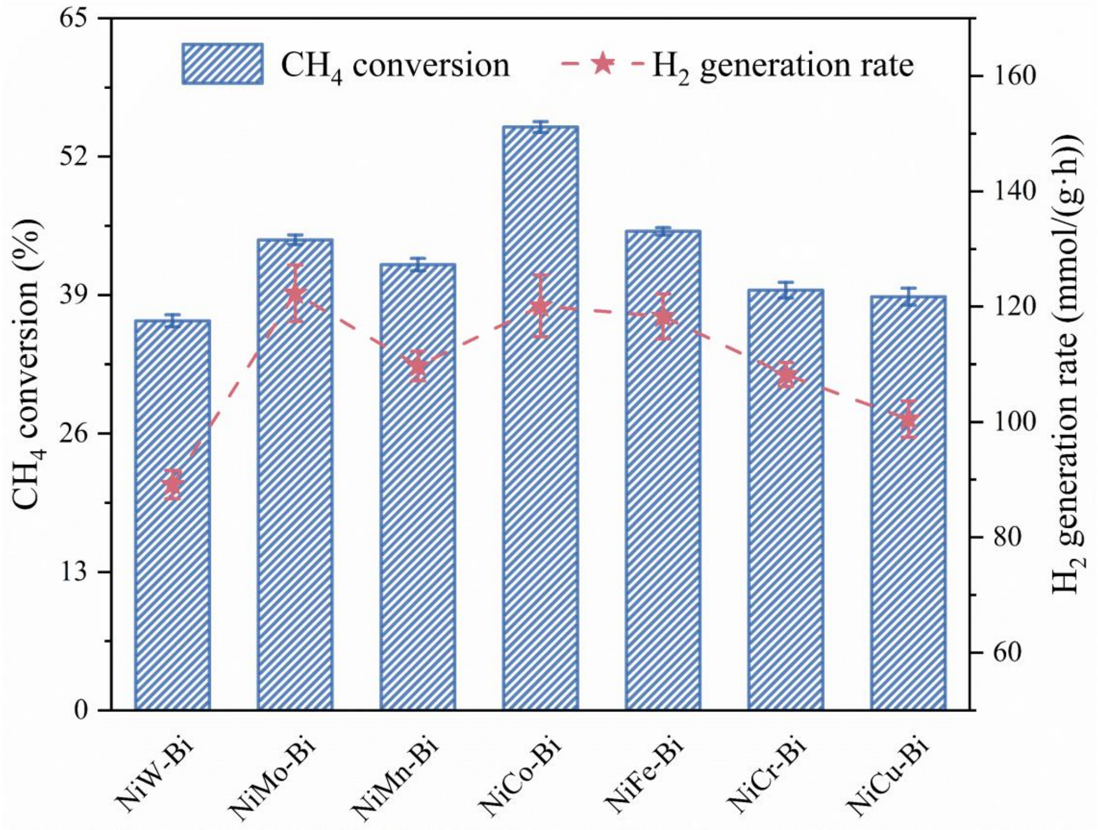

Figure S6. CH_4 conversion (left) and H_2 generation rate (right) of liquid alloy catalysts with different doping elements NiM-Bi (M = W, Mo, Mn, Co, Fe, Cr, Cu). Reaction temperature = 1000 ℃.

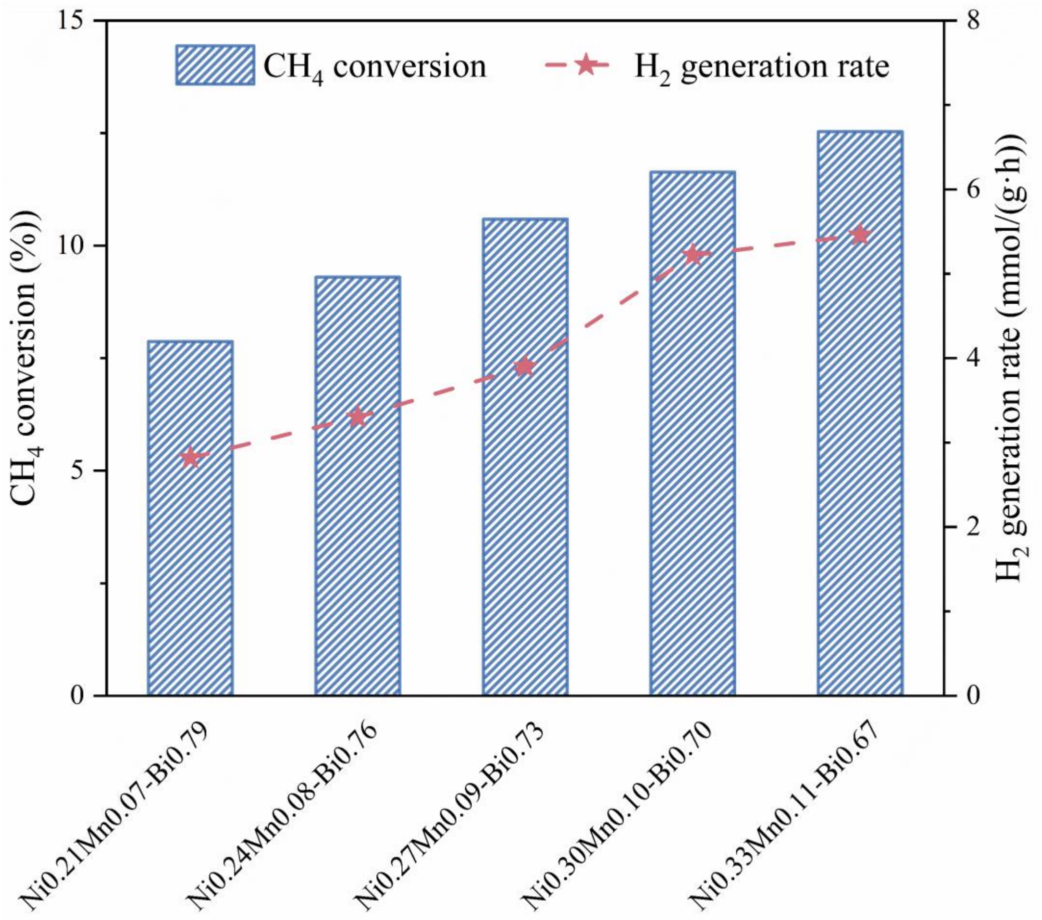

Figure S7. CH_4 conversion (left) and H_2 generation rate (right) of liquid alloy catalysts with different Ni-Bi molar ratio. The molar ratio of Ni-Mn is 3:1, Reaction temperature = 800 ℃.

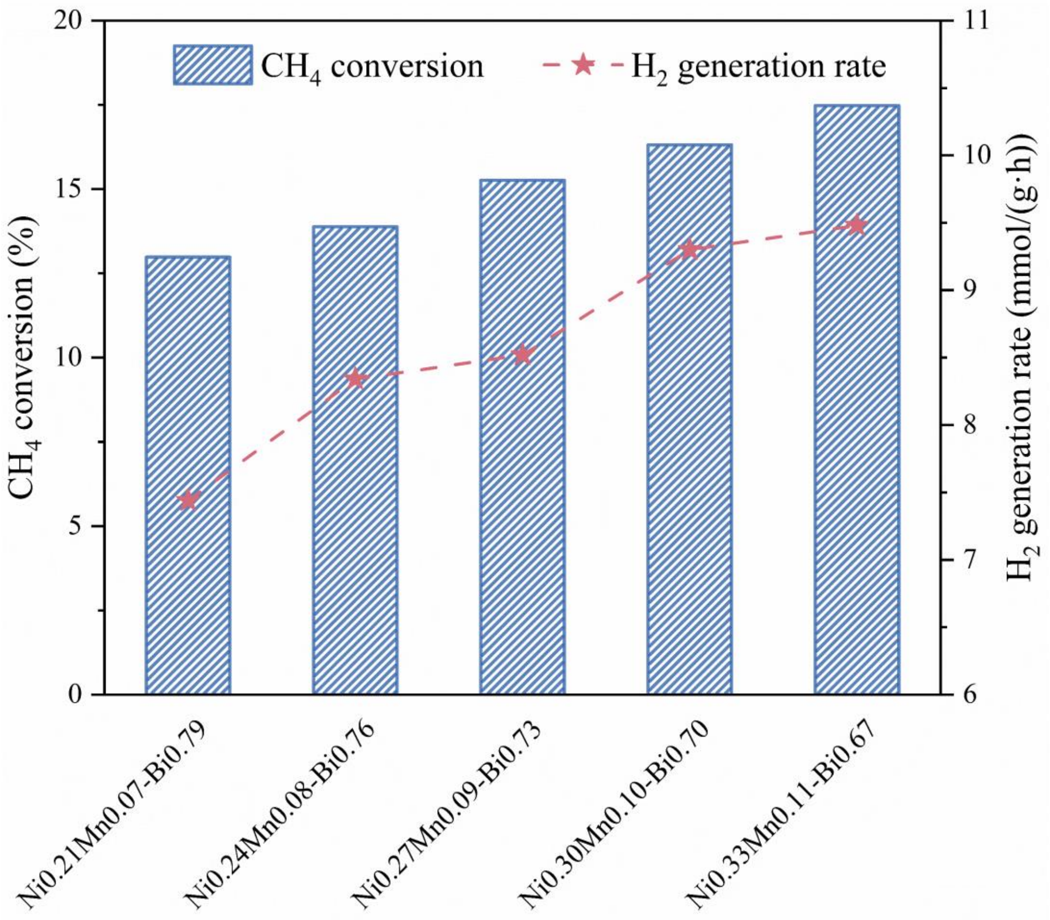

Figure S8. CH_4 conversion (left) and H_2 generation rate (right) of liquid alloy catalysts with different Ni-Bi molar ratio. The molar ratio of Ni-Mn is 3:1, Reaction temperature = 850 ℃.

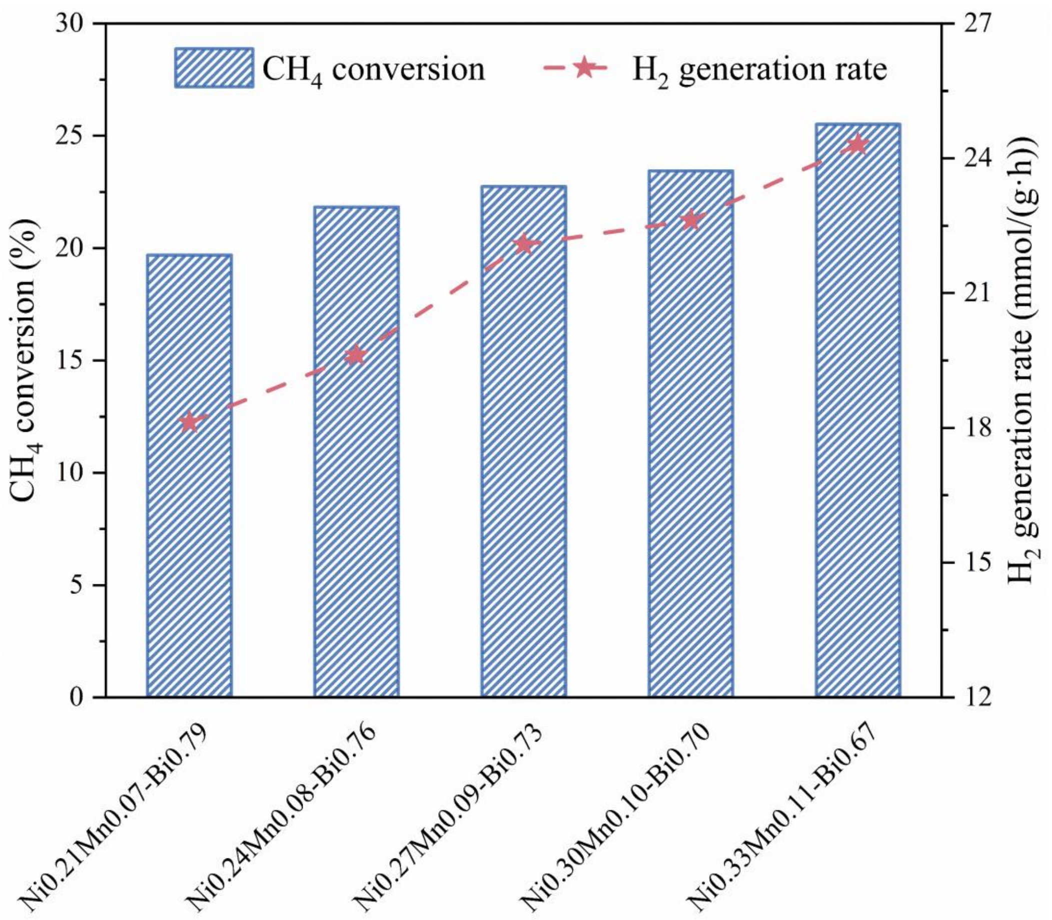

Figure S9. CH_4 conversion (left) and H_2 generation rate (right) of liquid alloy catalysts with different Ni-Bi molar ratio. The molar ratio of Ni-Mn is 3:1, Reaction temperature = 900 °C.

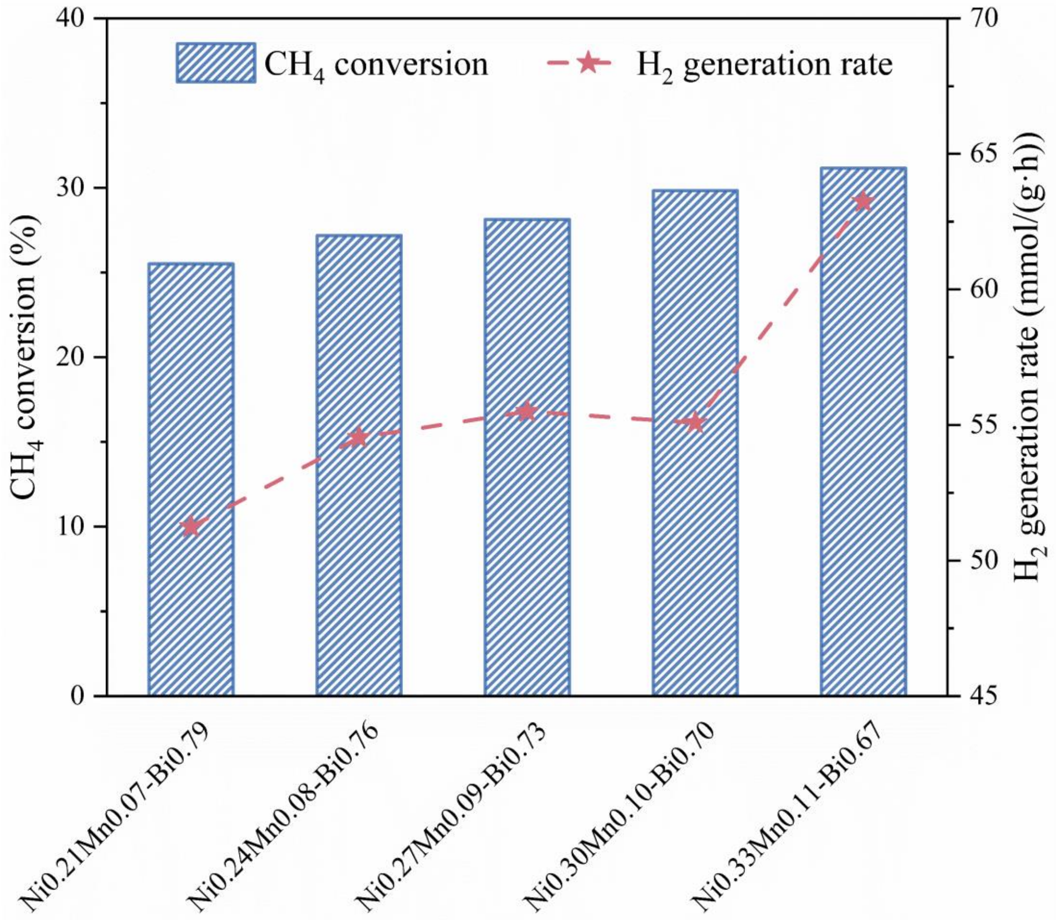

Figure S10. CH_4 conversion (left) and H_2 generation rate (right) of liquid alloy catalysts with different Ni-Bi molar ratio. The molar ratio of Ni-Mn is 3:1, Reaction temperature = 950 ℃.

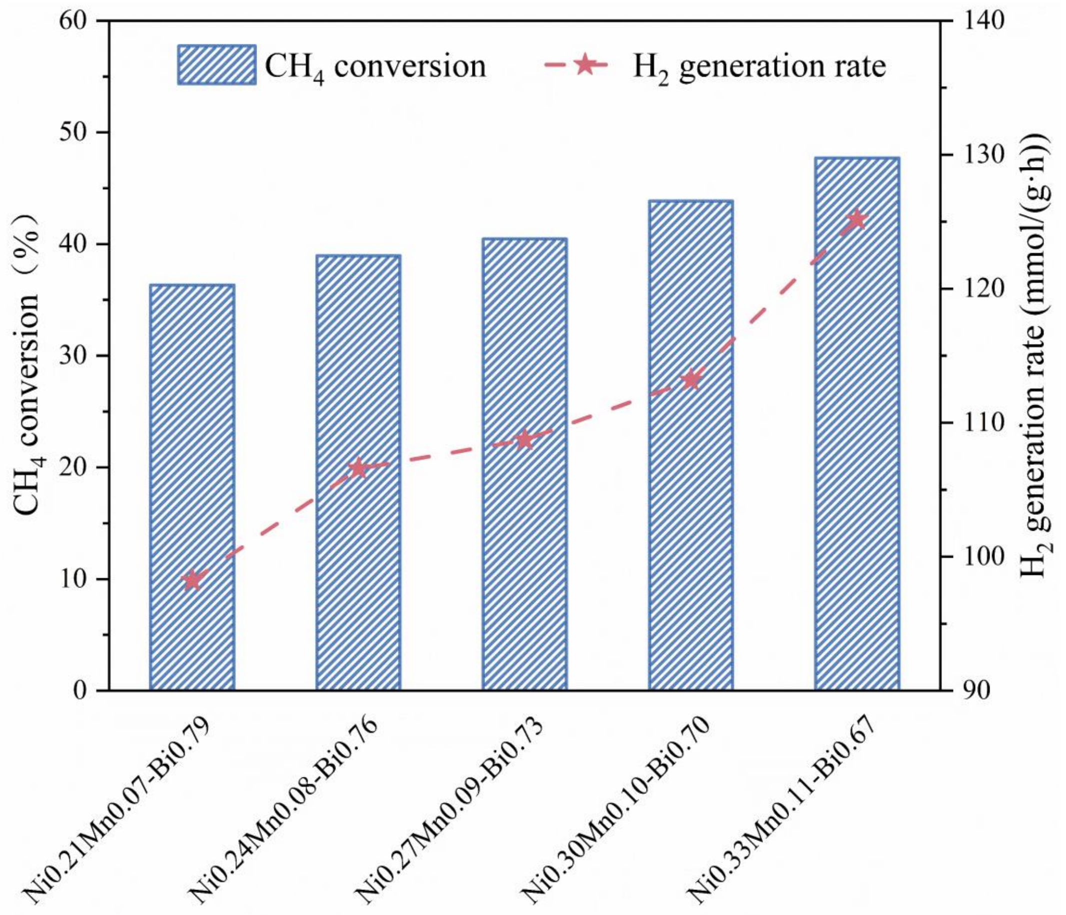

Figure S11. CH_4 conversion (left) and H_2 generation rate (right) of liquid alloy catalysts with different Ni-Bi molar ratio. The molar ratio of Ni-Mn is 3:1, Reaction temperature = 1000 ℃.

The performance of hydrogen production from methane decomposition over NiMn-Bi catalysts with different Ni-Bi molar ratios is shown in Figure S7-S11. Both methane conversion and hydrogen generation rate show an increasing trend with temperature as the Ni content increases.

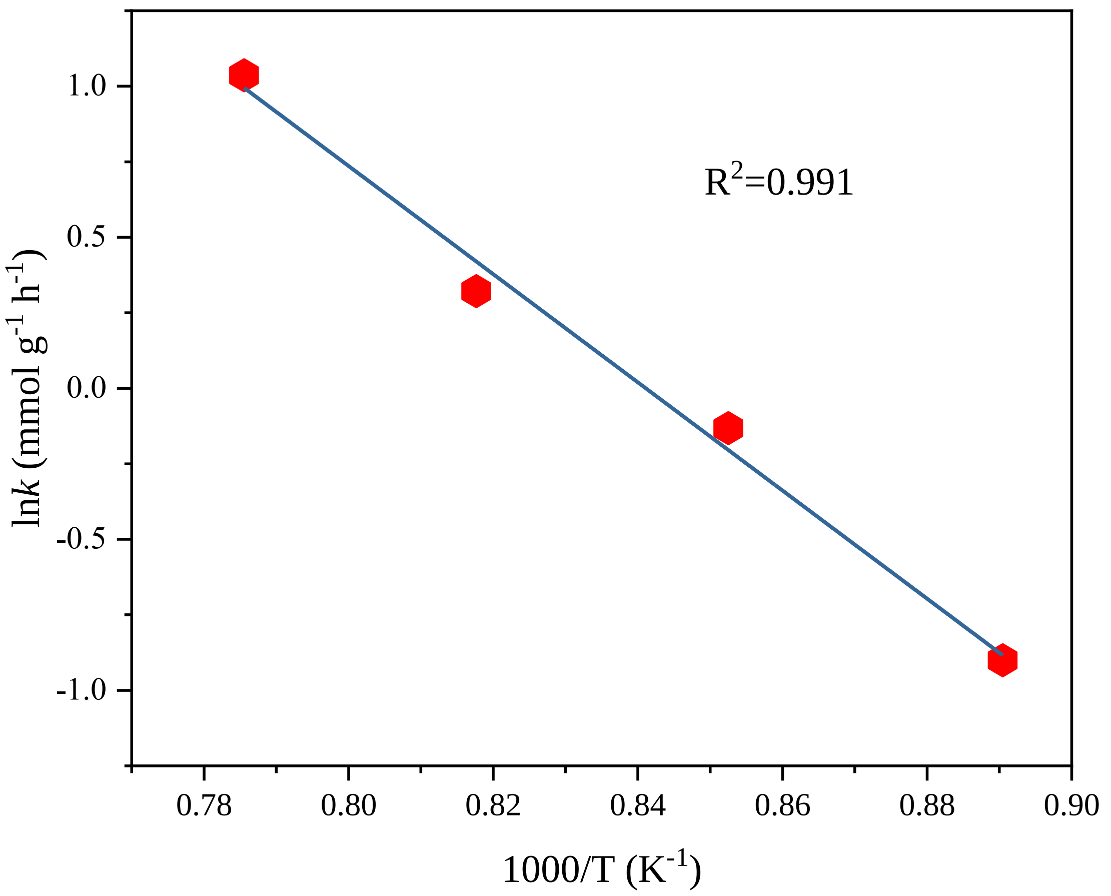

Figure S12. Apparent activation energy of the Ni_3Mn-Bi alloy catalyst under a CH_4 flow rate at 15 mL/min and an N_2 flow rate at 22.5 mL/min.

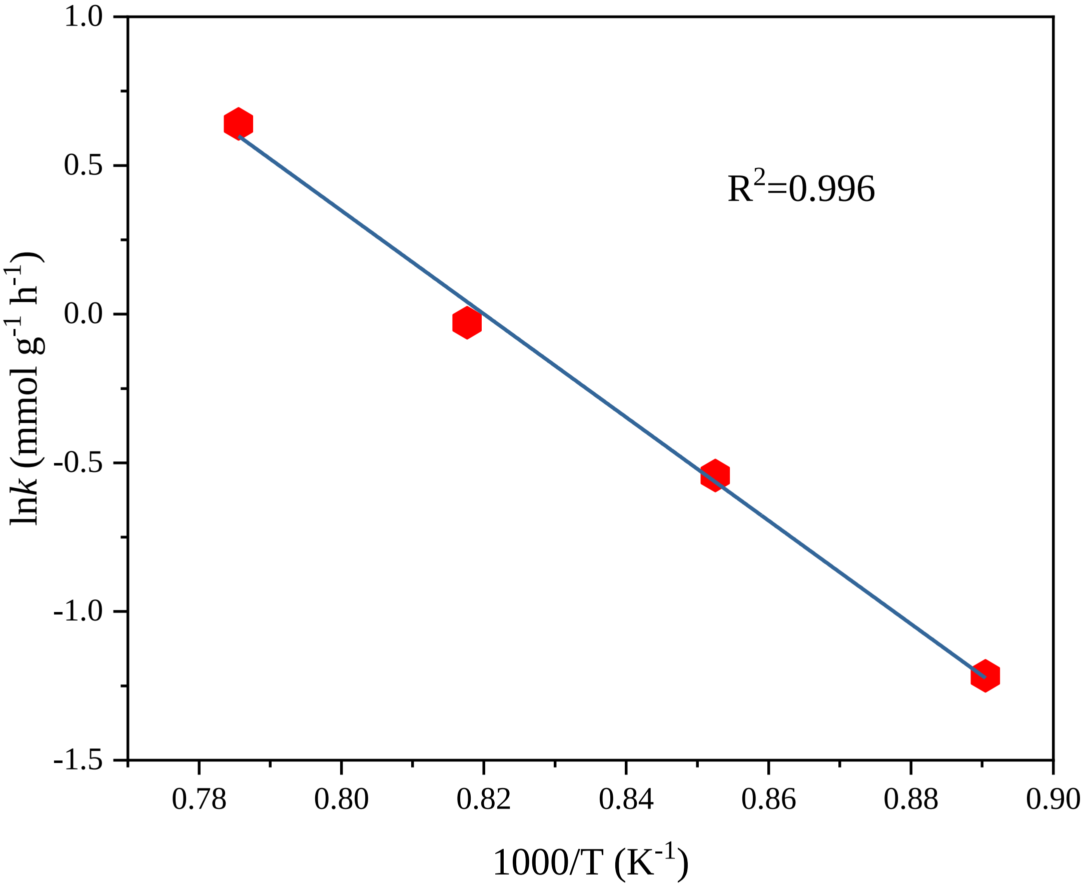

Figure S13. Apparent activation energy of the Ni_3Mn-Bi alloy catalyst under a CH_4 flow rate at 20 mL/min and an N_2 flow rate at 30 mL/min.

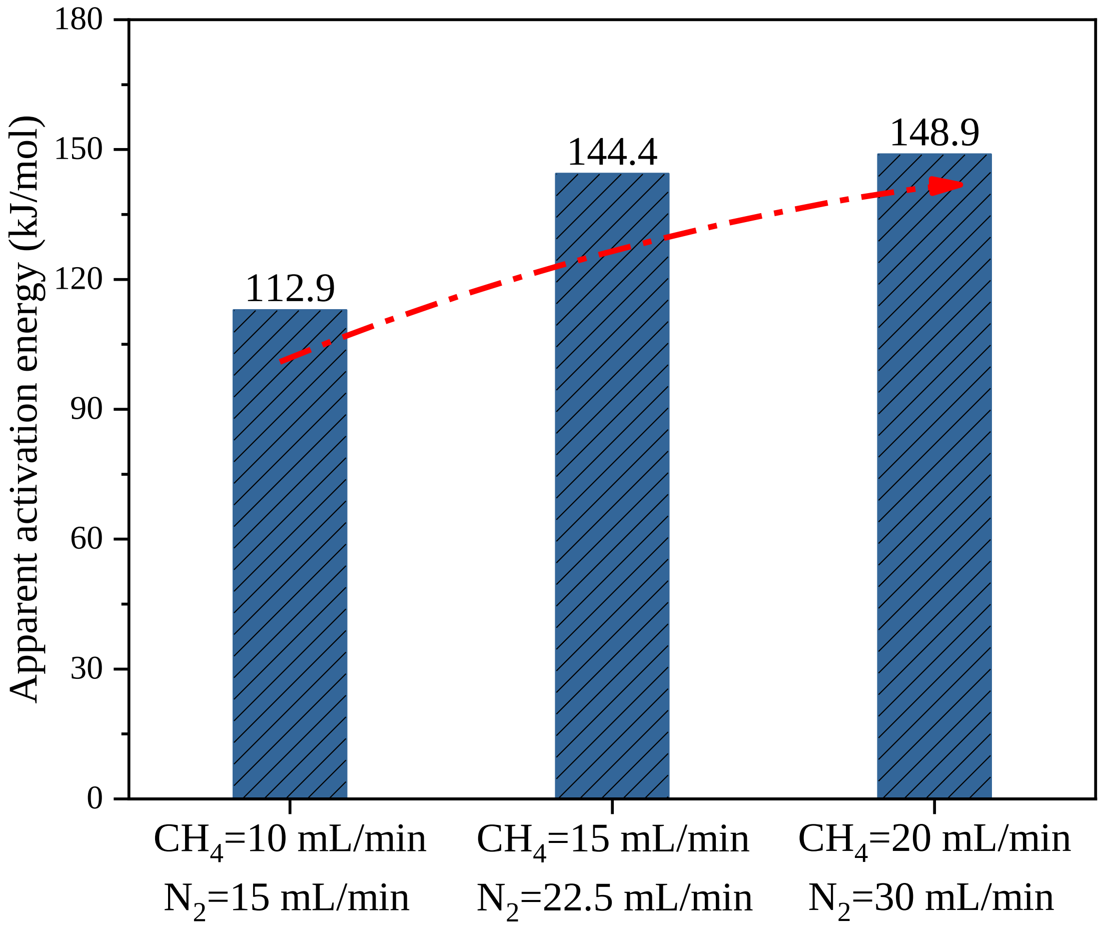

Figure S14. Comparisons of the apparent activation energy under different CH_4 and N_2 feeding rate.

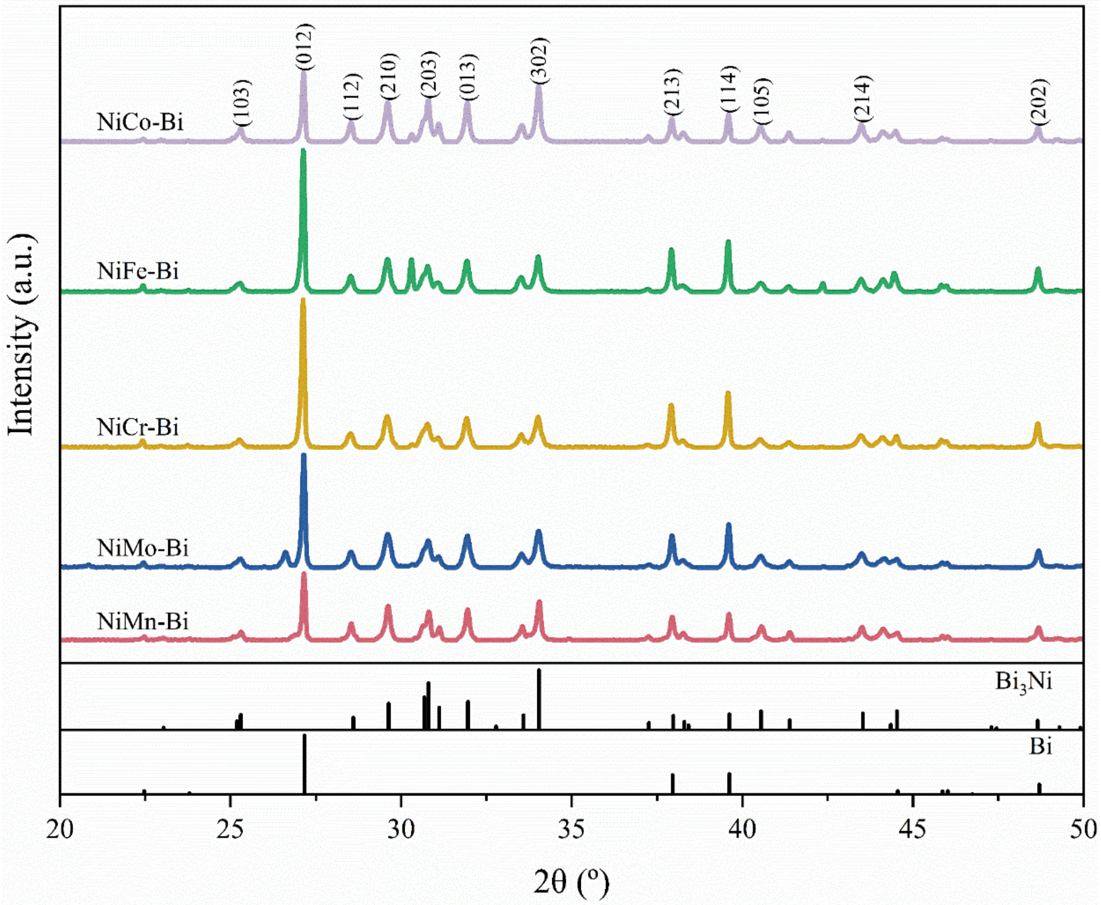

Figure S15. XRD patterns of different doping elements NiM-Bi (M = Mn, Mo, Cr, Fe, Co).

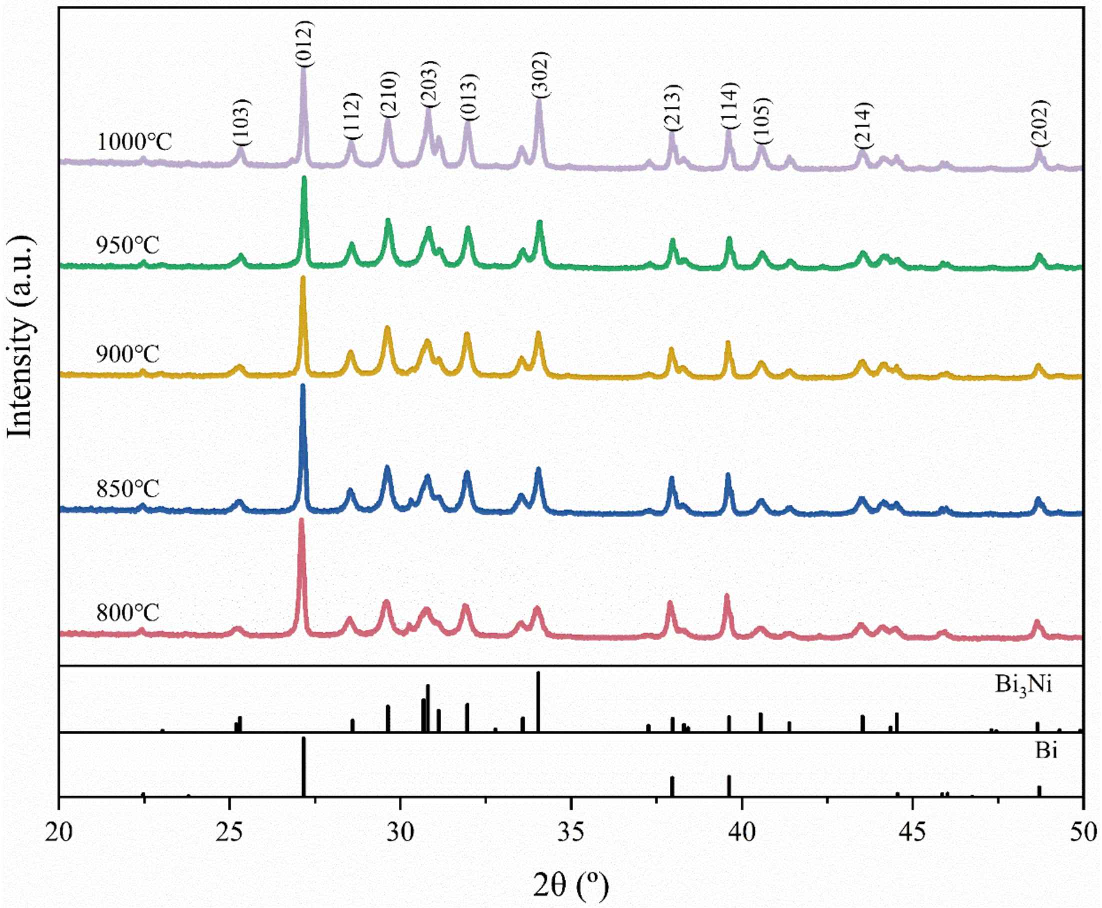

Figure S16. XRD patterns of Ni_3Mn-Bi catalysts with different reaction temperatures.

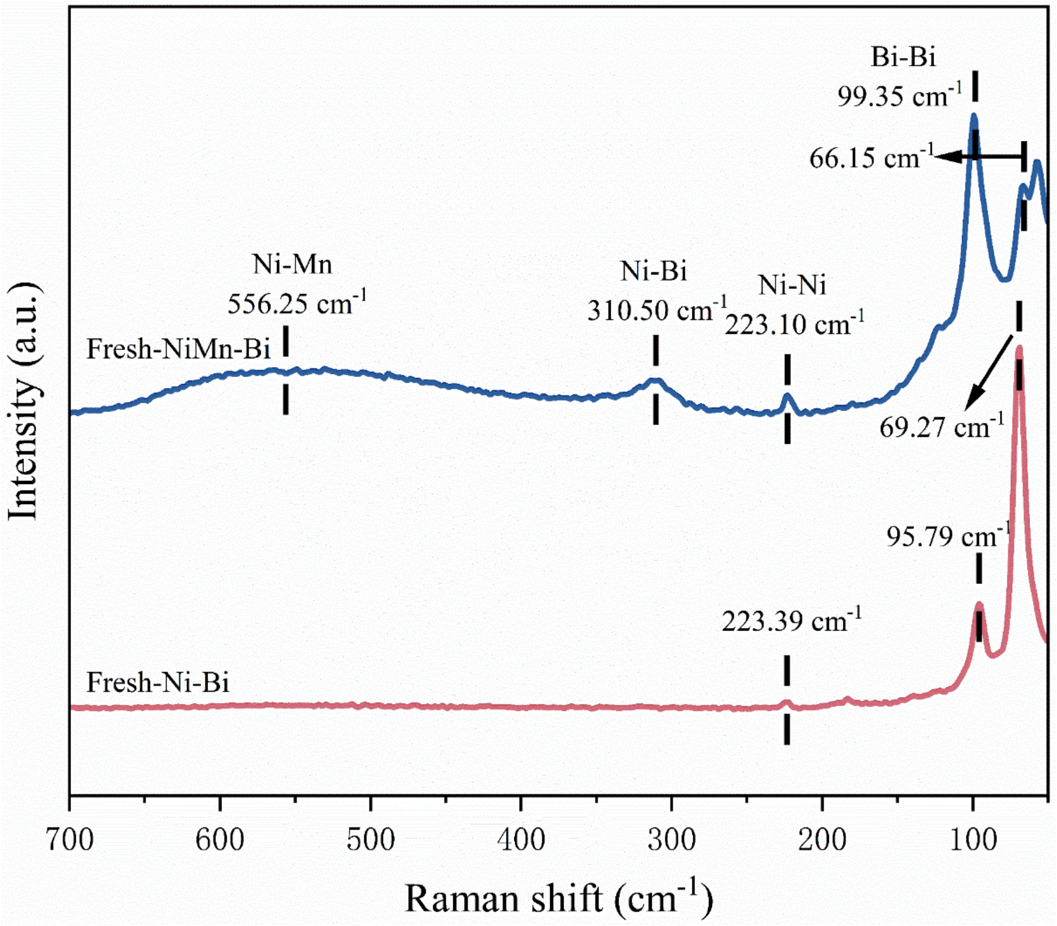

Figure S17. Raman spectra of fresh Ni-Bi, NiMn-Bi catalysts.

The characteristic peaks of Bi-Bi bonds usually appear in the lower wave number range (50-120 cm^{-1}). The doping of Ni and Mn induced lattice distortions and vibrational frequency changes, which resulted in a shift in the position of the Raman peaks of the Bi-Bi bonds in the Ni-Bi versus NiMn-Bi samples. The addition of Mn not only changed the Ni-Ni interactions, but also further modulated the surface structure and electronic properties of the catalysts through the formation of Ni-Mn bonds (560.10 cm^{-1}). In addition, the Ni-Bi structural peak observed at 310.68 cm^{-1} showed a low-wave number shift in the Ni-Bi structure in NiMn-Bi due to the alteration of the electronic environment and local structure of Ni by Mn doping. This suggests that the activity and selectivity of the catalyst can be effectively regulated and its catalytic performance can be further optimized by precisely regulating the types and ratios of the doping elements (Figure S17).

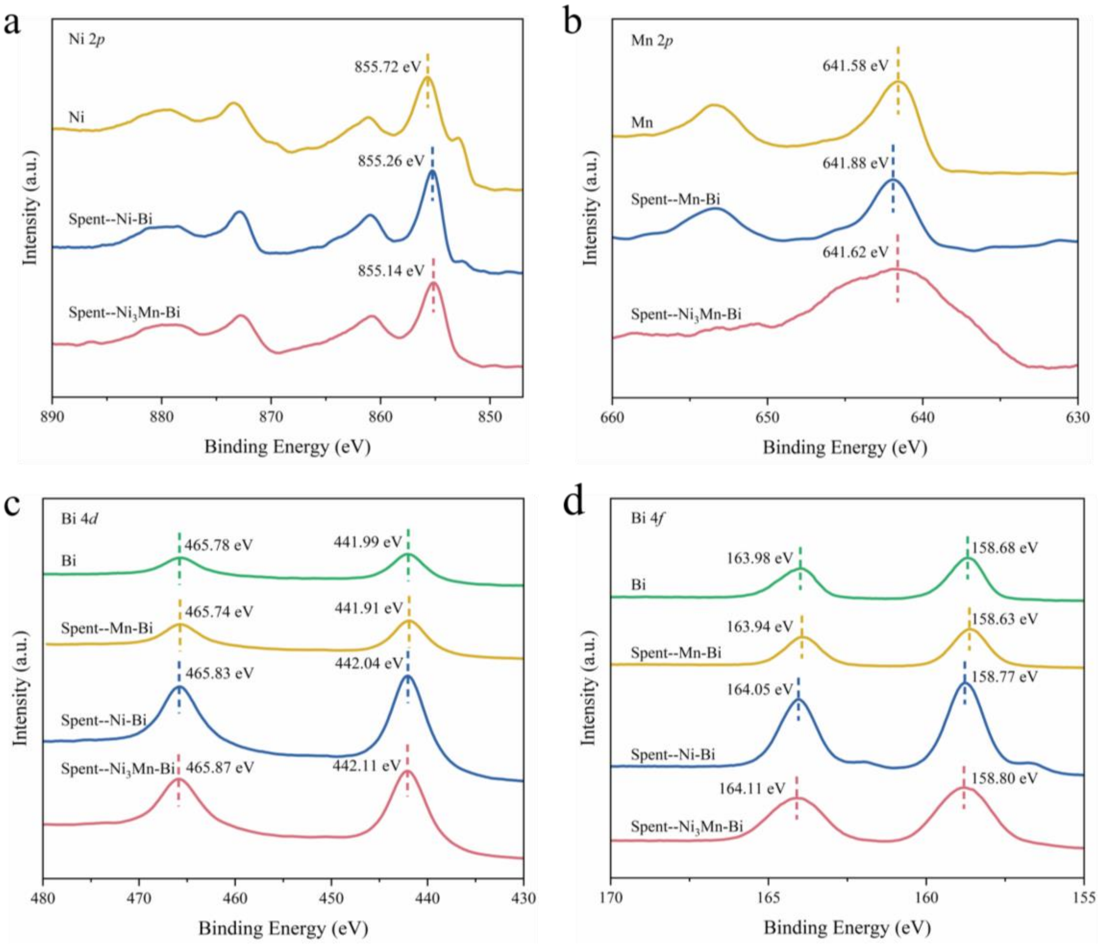

Figure S18. XPS analysis of the spent catalysts Ni-Bi, Ni_3Mn-Bi and Mn-Bi catalysts. a) Ni 2*p*. b) Mn 2*p*. c) Bi 4*d*. d) Bi 4*f*.

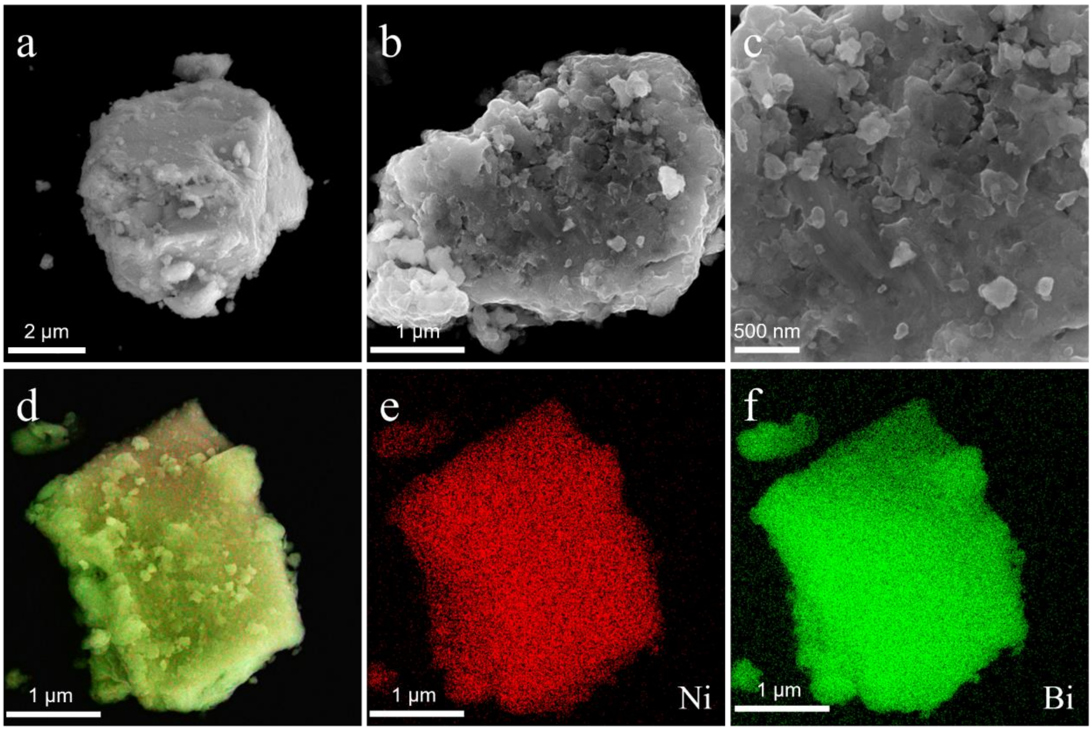

Figure S19. SEM characterization of Ni-Bi catalysts. a-d) Morphology and particle structure of catalysts at different magnifications. e) Energy dispersive X-ray spectroscopy (EDS). f) EDS mapping of Ni-Bi. g) EDS mapping of the element Ni. h) EDS mapping of the element Bi.

Figure S19 shows the SEM characterization of the microstructure, morphology and porosity of the catalyst surface. The SEM results of the Ni-Bi catalyst showed a relatively flat surface of the particles and a relatively regular morphology, indicating a relatively homogeneous structure of the catalyst (Figure S19a-c). The elemental spectra of Figure S19d demonstrate significant signal peaks of Ni and Bi elements, which are further shown by the elemental mapping diagrams S19d-f, indicating that the distribution of Ni and Bi elements in the catalyst is relatively homogeneous. In contrast, the Ni_3Mn-Bi catalyst in Figure S20 differs in morphology and structure.

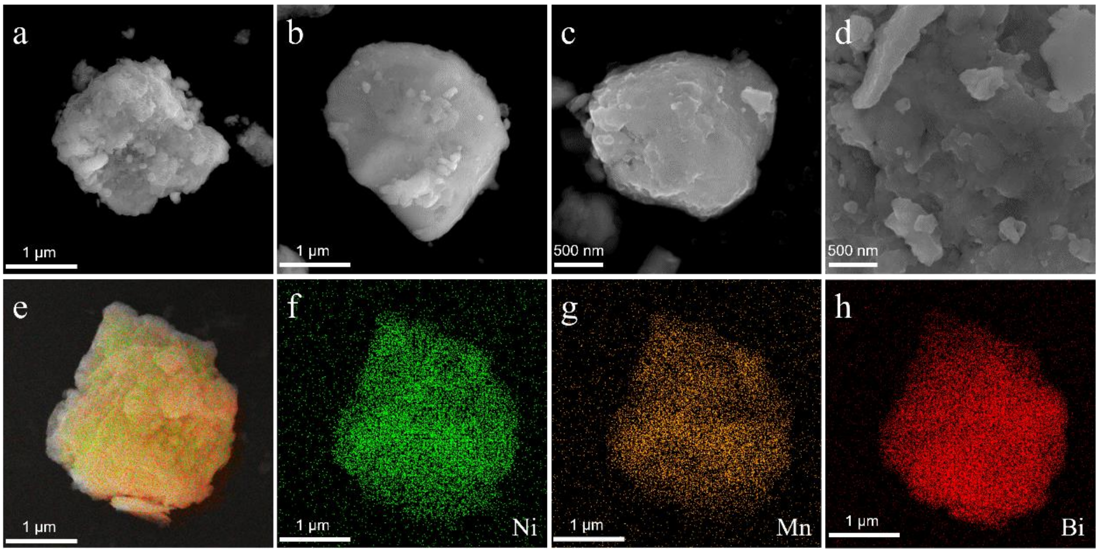

Figure S20. SEM characterization of Ni_3Mn-Bi catalysts. a-d) Morphology and particle structure of catalysts at different magnifications. e) EDS mapping of Ni_3Mn-Bi. f) EDS mapping of the element Ni. g) EDS mapping of the element Mn. h) EDS mapping of the element Bi.

The porous structure of the catalyst particles and the obvious nanoscale features in Figure S20a-d indicate that they have a large specific surface area, which is favorable for the contact and reaction between the catalyst and the reactants. The uniform distributions of Ni, Mn and Bi are shown in Figure S20e-h, especially the introduction of Mn, which complicates the multicomponent properties of the catalysts. The introduction of Mn not only enhances the dispersion of active sites, but also may improve the methane decomposition performance of the catalysts by modulating the electronic structure of Ni, which allows the catalysts to maintain a high activity during the long-term reaction process.

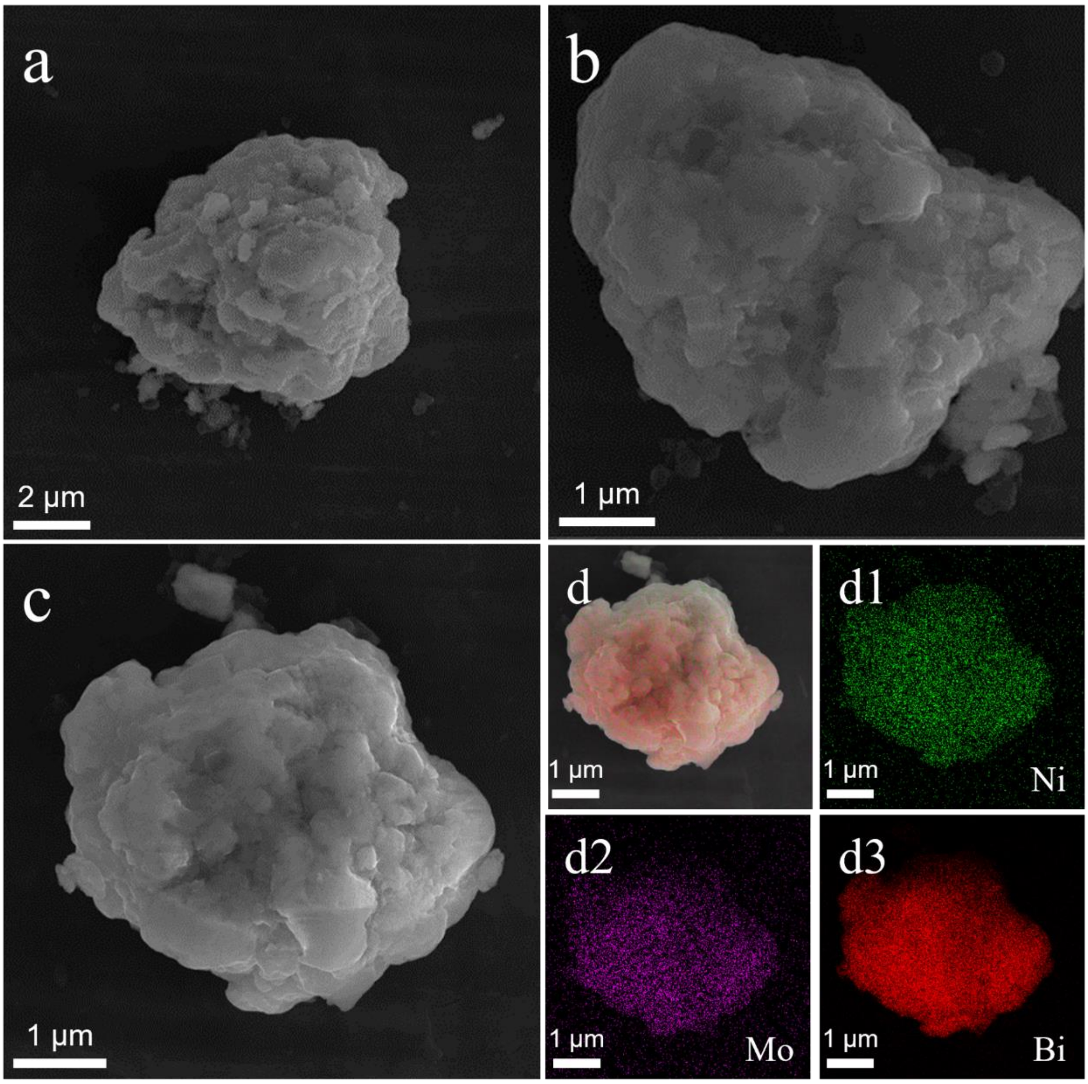

Figure S21. SEM characterization of Ni_3Mo-Bi catalysts. a-c) Morphology and particle structure of Ni_3Mo-Bi catalysts at different magnifications. d) EDS mapping of Ni_3Mo-Bi catalysts. d1-d3) EDS mapping of Ni, Mo, Bi element.

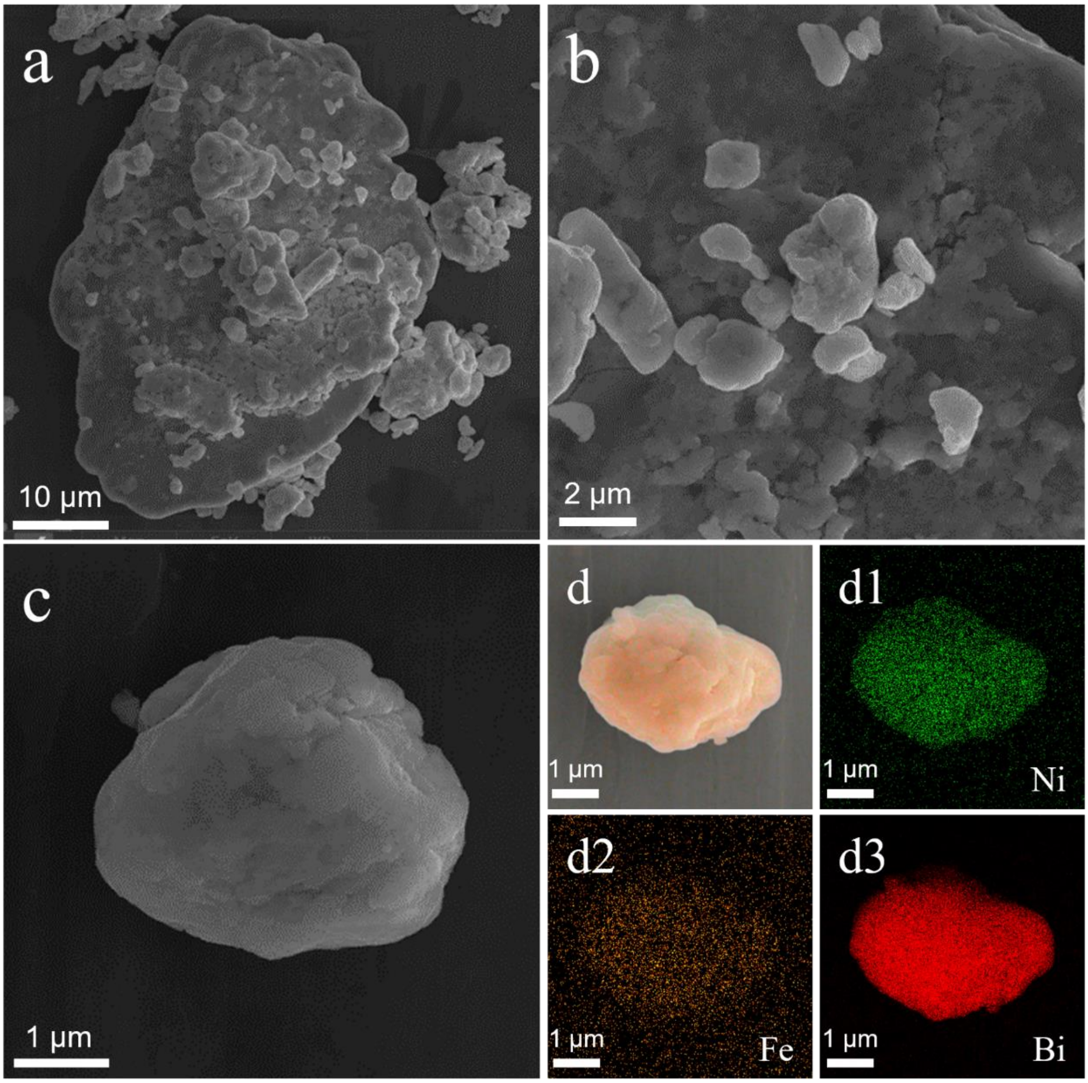

Figure S22. SEM characterization of Ni_3Fe-Bi catalysts. a-c) Morphology and particle structure of Ni_3Fe-Bi catalysts at different magnifications. d) EDS mapping of Ni_3Fe-Bi catalysts. d1-d3) EDS mapping of Ni, Fe, Bi element.

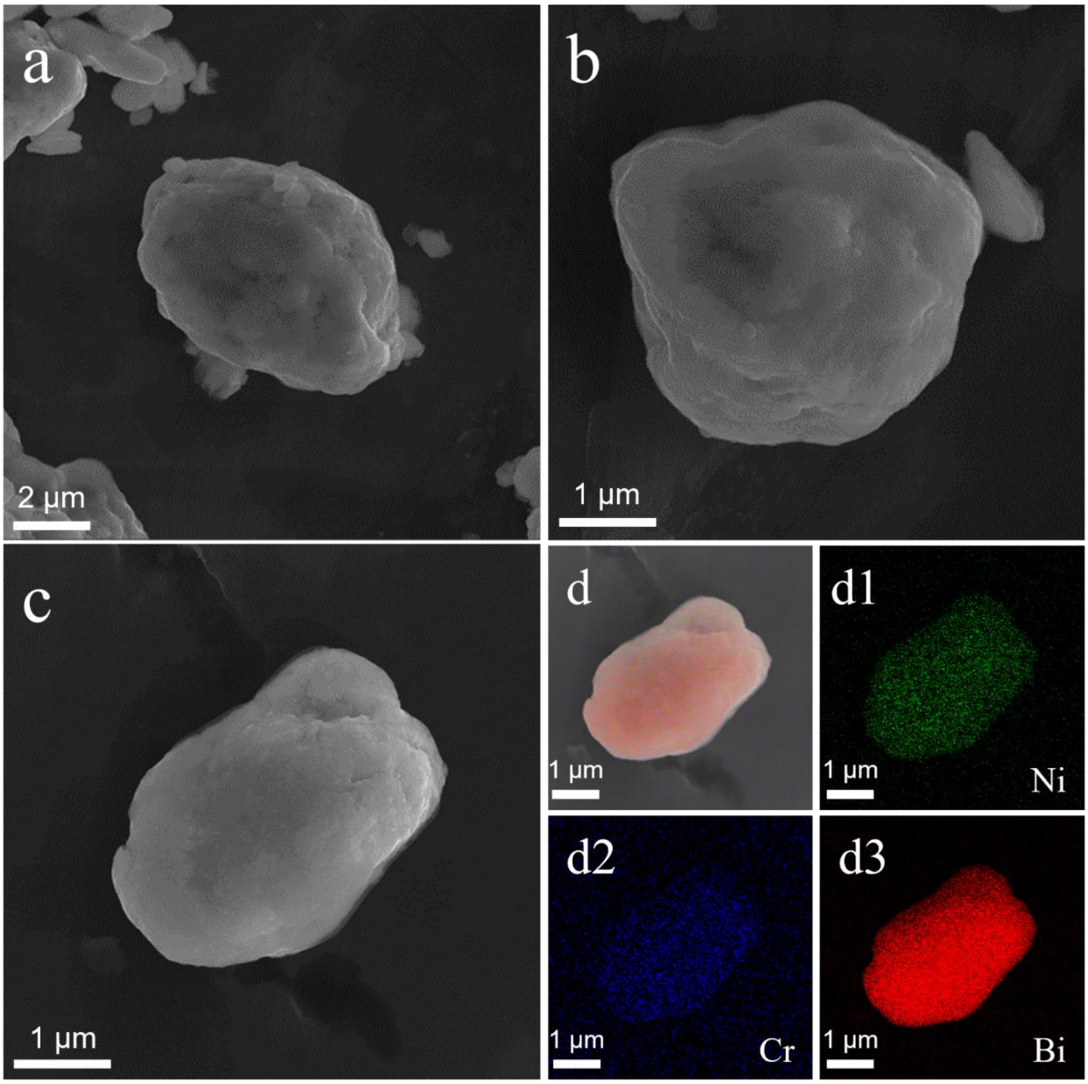

Figure S23. SEM characterization of Ni_3Cr-Bi catalysts. a-c) Morphology and particle structure of Ni_3Cr-Bi catalysts at different magnifications. d) EDS mapping of Ni_3Cr-Bi catalysts. d1-d3) EDS mapping of Ni, Cr, Bi element.

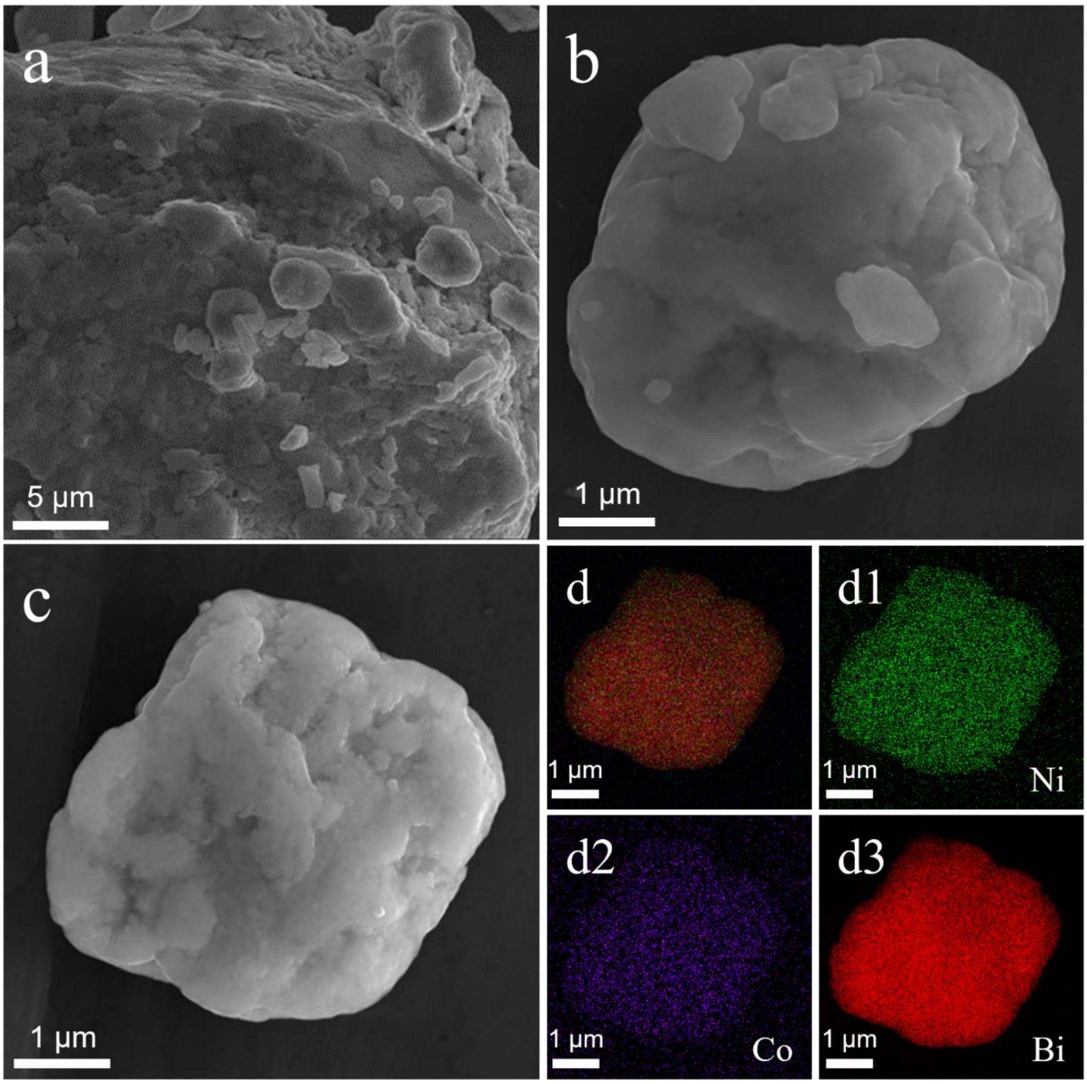

Figure S24. SEM characterization of Ni_3Co-Bi catalysts. a-c) Morphology and particle structure of Ni_3Co-Bi catalysts at different magnifications. d) EDS mapping of Ni_3Co-Bi catalysts. d1-d3) EDS mapping of Ni, Co, Bi element.

In addition, the morphology and elemental distribution of the catalysts Ni_3M-Bi (M=Mo, Fe, Cr, Co) with different doping metals were also characterized by SEM, and the detailed results are shown in Figure S21-S24. The different doping elements had a significant effect on the microstructure and elemental distribution of the Ni-Bi-based catalysts, which suggests that the physicochemical properties of the catalysts can be optimized effectively by adjusting the doping elements to further enhance the performance of the catalysts in methane decomposition to hydrogen. Its catalytic performance in the hydrogen production reaction from methane decomposition.

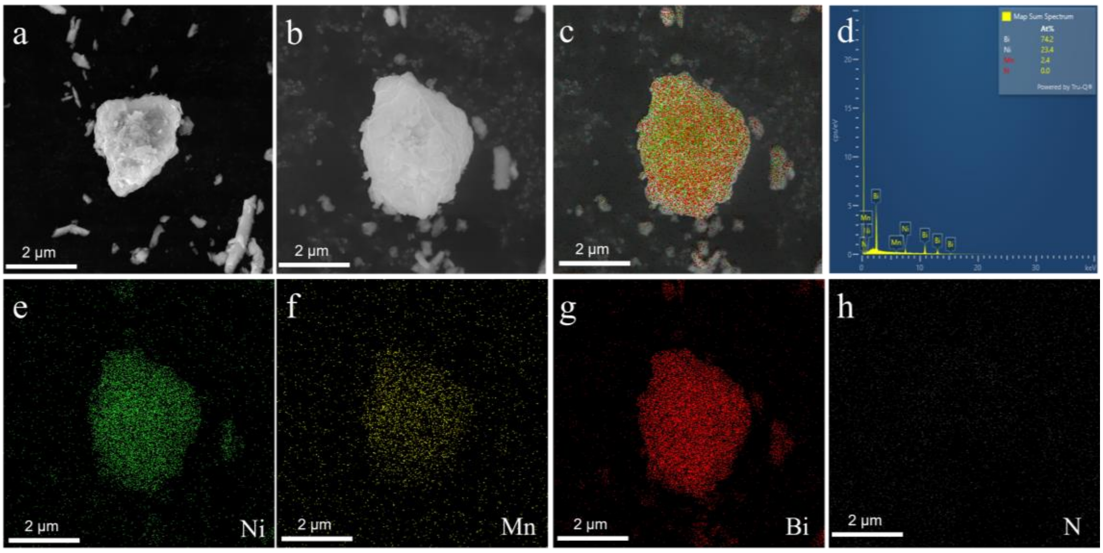

Figure S25. SEM characterization of Ni_3Mn-Bi catalysts. a-b) Morphology and particle structure of Ni_3Mn-Bi catalysts. c) EDS mapping of Ni_3Mn-Bi catalysts. d) Elemental analysis. e-h) EDS mapping of Ni, Mn, Bi, and N element.

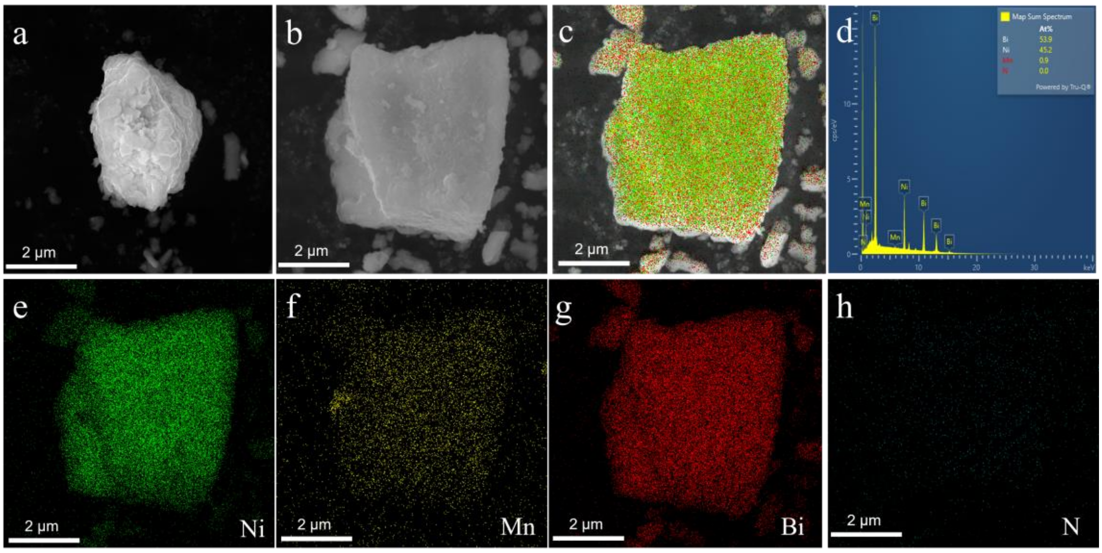

Figure S26. SEM characterization of Ni_3Mn-Bi catalysts. a-b) Morphology and particle structure of Ni_3Mn-Bi catalysts. c) EDS mapping of Ni_3Mn-Bi catalysts. d) Elemental analysis. e-h) EDS mapping of Ni, Mn, Bi, and N element.

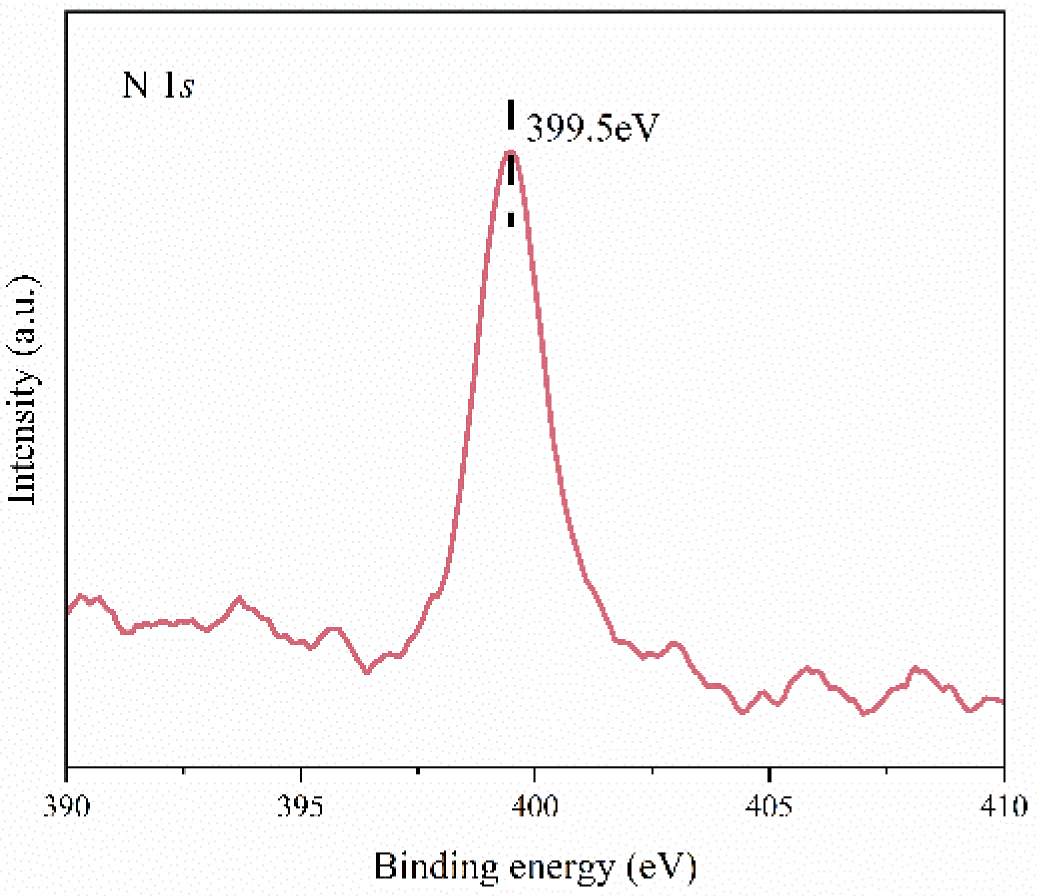

Figure S27. XPS result of N1*s* for the 2 h reacted Ni_3Mn-Bi alloy catalyst.

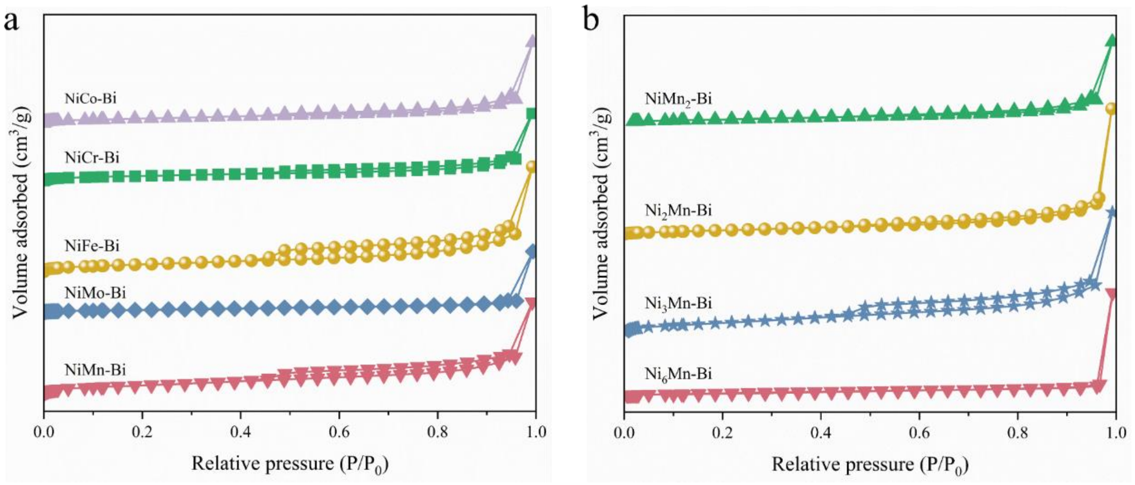

Figure S28. N_2 adsorption-desorption isothermal curves of catalysts after methane decomposition reaction. a) NiM-Bi (M =Mn, Mo, Fe, Cr, Co) catalysts with different doping elements. b) NiMn-Bi catalysts with different Ni-Mn molar ratios.

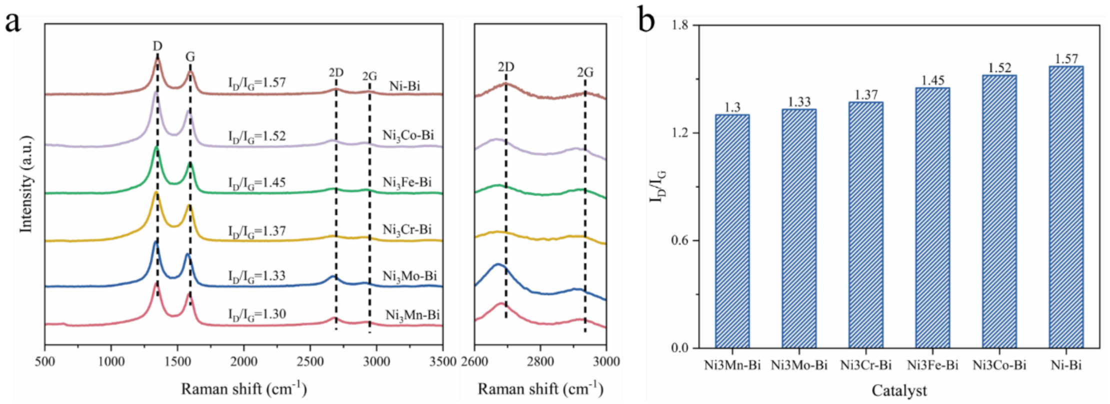

Figure S29. Raman spectra of a) Solid carbon of Ni-Bi and NiM-Bi (M = Mn, Mo, Cr, Fe, Co) catalysts with different doping elements. b) I_D/I_G values of different doping elements.

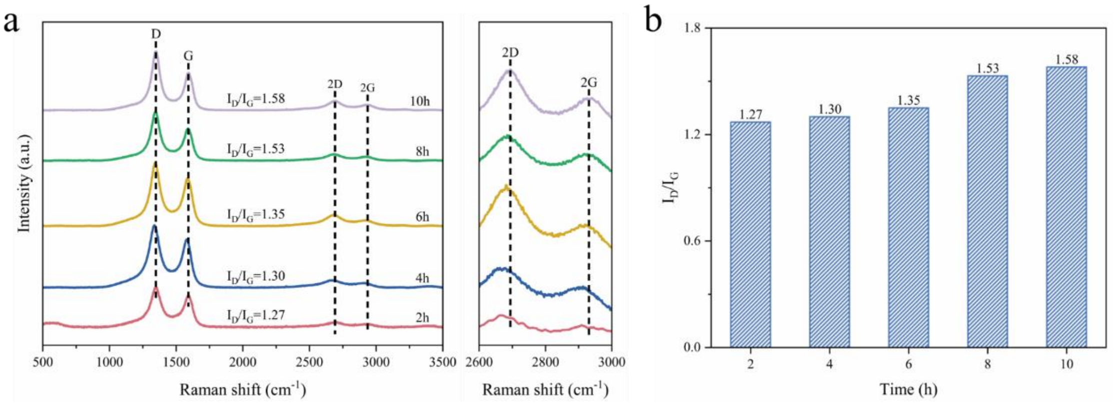

Figure S30. Raman spectra of a) Different reaction times of Ni_3Mn-Bi catalyst. b) I_D/I_G values of different reaction times.

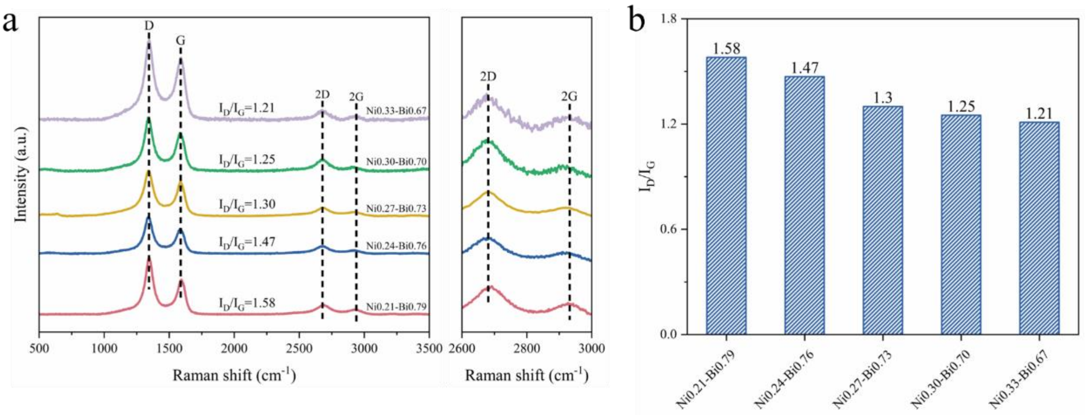

Figure S31. Raman spectra of catalysts with a) Different Ni-Bi molar ratios. b) I_D/I_G values of catalysts with different Ni-Bi molar ratios.

Raman spectroscopy confirmed the impact of catalyst types, Ni:Mn ratios, Ni:Bi ratios, CMD temperatures and duration on the graphitization degree of the carbon generated (Figure S29-S31 and Figure S32a-d). The characteristic peaks obtained mainly include D, G, 2D, and 2G bands, corresponding to i) the disordered structures such as boundary defects, dislocations, and lattice defects in the sp^3-hybridized carbon structure; ii) planar vibrational modes of sp^2 carbon-carbon bonds in graphitic materials, representing graphitized carbon structures; iii) the layered structure of the carbon material and its crystal thickness; iv) the lattice vibration properties of sp^2 bonded carbon atoms in carbon microtubes were determined[1-3]. Specifically, of the tested ternary alloy catalysts, the I_D/I_G values follow the order of Ni_3Mn-Bi < Ni_3Mo-Bi < Ni_3Cr-Bi < Ni_3Fe-Bi < Ni_3Co-Bi, implying the formation of highly graphitized carbon catalyzed by Ni_3Mn-Bi. In addition, doping elements (Mn, Mo, Cr, Fe, Co) may introduce structural defects or form amorphous carbon regions by affecting the nucleation and growth mode of carbon atoms, leading to increased disorder[4]. The graphitization degree of carbon shows a positive correlation with the Ni:Mn mole ratios, i.e., increasing Mn:Ni ratio tends to deposit disordered amorphous carbon. High temperature and prolonged reaction time lead to the accumulation of defects and the increase of disordered structures in carbon materials, thus inhibiting further graphitization of carbon[5, 6]. The intensities of the 2D and 2G peaks of all carbon materials are generally low, suggesting that the carbon materials are poorly ordered and

lack the typical monolayer graphene structure, which is characterized by a multilayer structure[7].

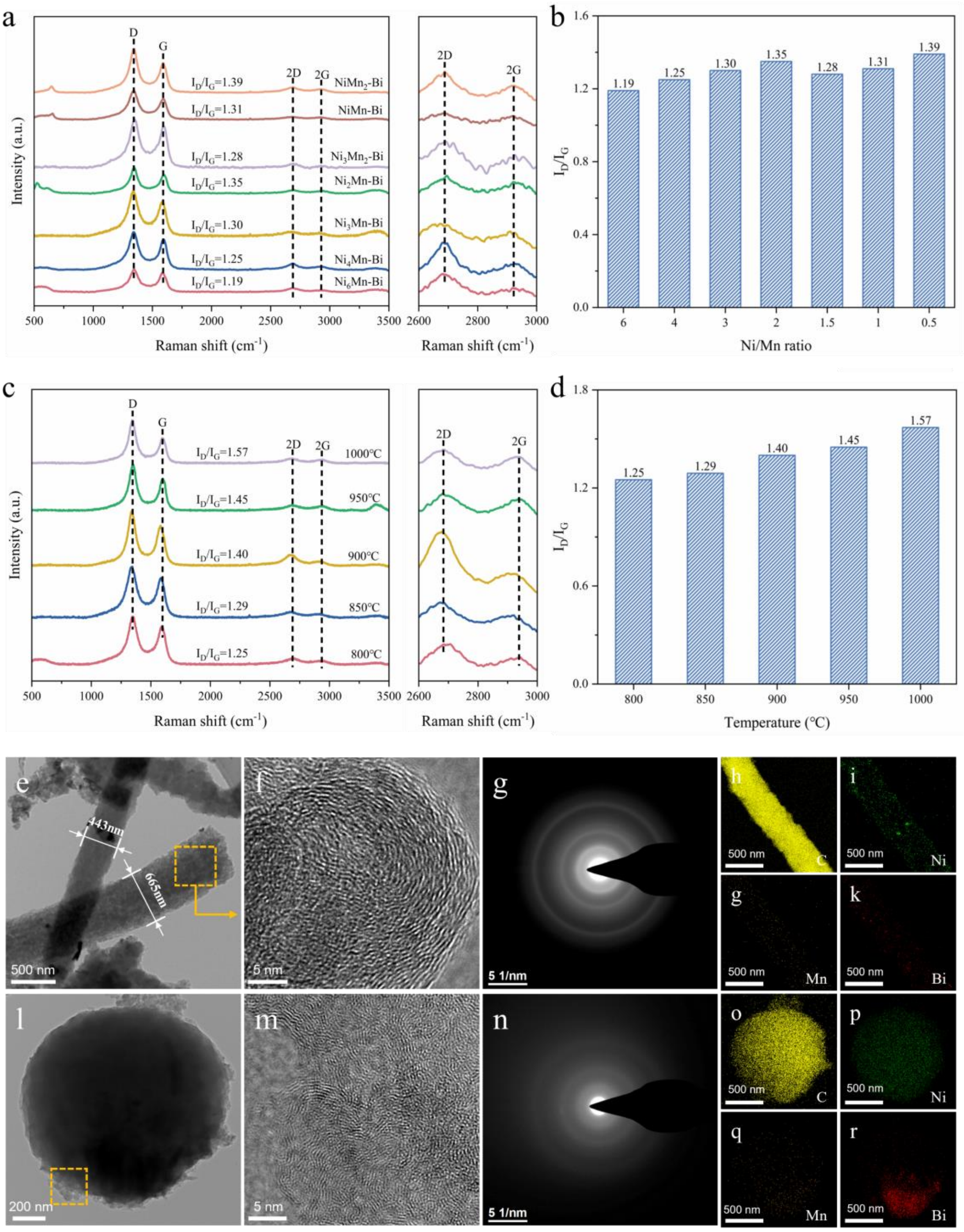

Figure S32. Raman spectra and TEM characterization of solid carbon under different reaction conditions. a) Different Ni-Mn molar ratios (850 °C). b) I_D/I_G values of different Ni-Mn molar ratios. c) Different reaction temperatures (Ni_3Mn-Bi). d) I_D/I_G values of different reaction temperatures. e) TEM images. f) HR-TEM images. g) SAED pattern. h-k) C, Ni, Mn, Bi elemental distribution. l) TEM images. m) HR-TEM images. n) SAED maps. o-r) C, Ni, Mn, Bi elemental distribution maps.

The morphology of CMD derived carbon materials were characterized by TEM, and both carbon microtube and spherical structures were observed (Figure S32e-r).

Specifically, the size of carbon microtubes ranged within 443-665 nm, corresponding to a nanotube with 1303-1956 layers. HR-TEM images reveal the generation of layered and graphitized carbon as well as the disordered and amorphous carbon, as shown in Figure S32f and Figure S32m, respectively. SAED results from Figure S32g demonstrate clear diffraction rings, confirming the high crystallinity and graphitized structure of the carbon material, conversely, in Figure S32n the diffraction rings are diffuse in accordance with the different graphitization degrees analyzed above. EDS mapping results from Figure S32h-k and S32o-r indicate that both carbon types show highly concentrated distribution, while that of Ni, Mn, and Bi elements is relatively sparse, indicating a very small amount of metal residue.

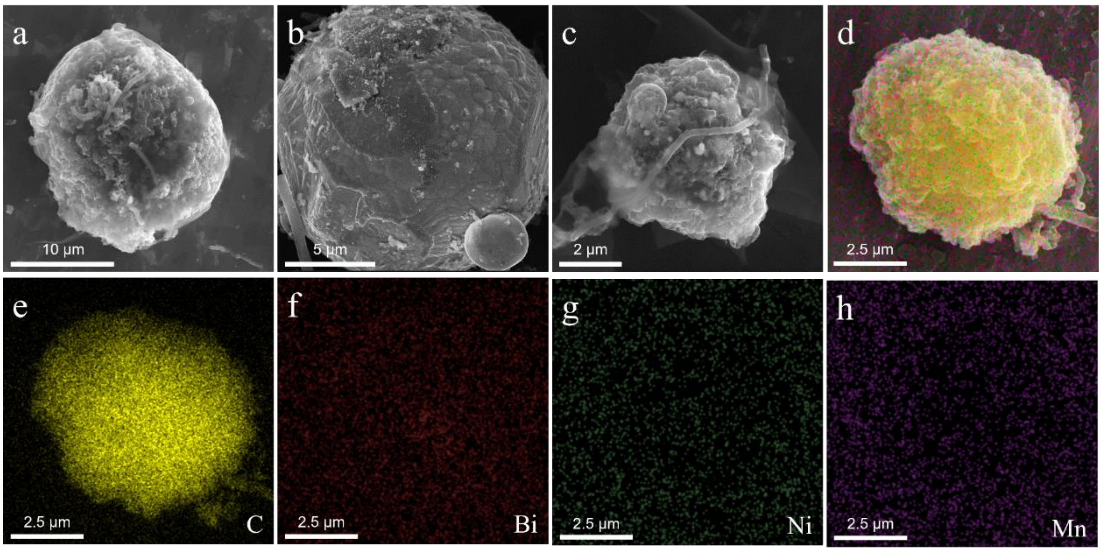

Figure S33. SEM results a-d) SEM images of solid carbon derived from CMD at 850 °C for 2 h and catalyzed by Ni_3Mn-Bi. e) EDS mapping results of (e) C; (f) Bi; (g) Ni; and (h) Mn elements.

SEM results confirm the co-generation of carbon microtubes and spherical morphologies under various Ni-Mn-Bi ternary alloy catalysts (Figure S33-S43). More interestingly, we found that introduction of Ni greatly affects the proportions of spherical carbon and carbon microtubes. With the rise of Ni-to-Bi ratio from 0.21 to 0.33, the carbon material transitions from amorphous carbon to carbon microtubes. Specifically, the carbon microtubes fraction reached 10%, 40%, and almost 100% under Ni-to-Bi ratios of 0.21, 0.27, and 0.33, respectively.

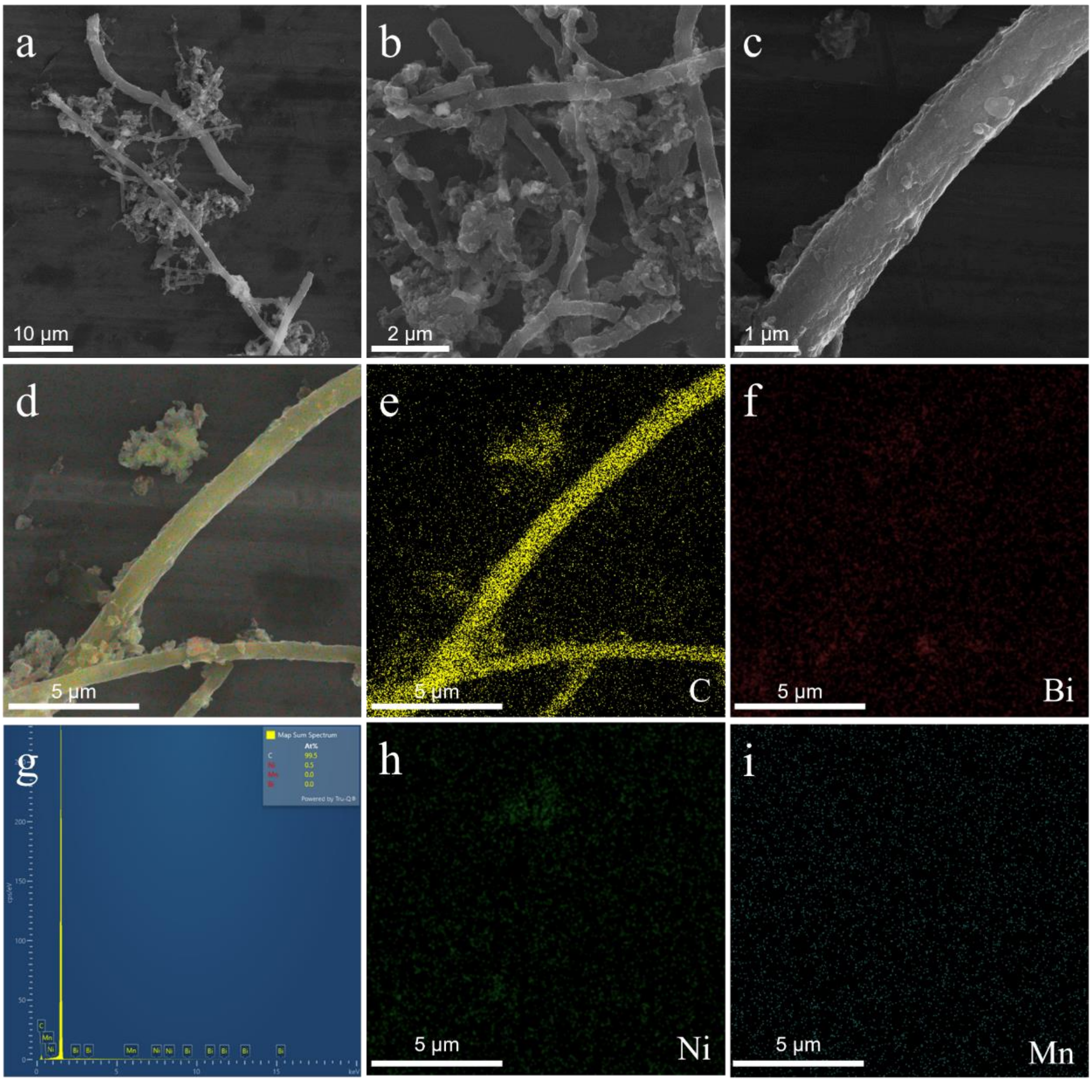

Figure S34. SEM results a-c) SEM images of carbon microtubes derived from CMD at 850 °C for 2 h and catalyzed by Ni_3Mn-Bi. d) EDS mapping results of (e) C; (f) Bi; (h) Ni; and (i) Mn elements. g) Energy dispersive X-ray spectroscopy (EDS).

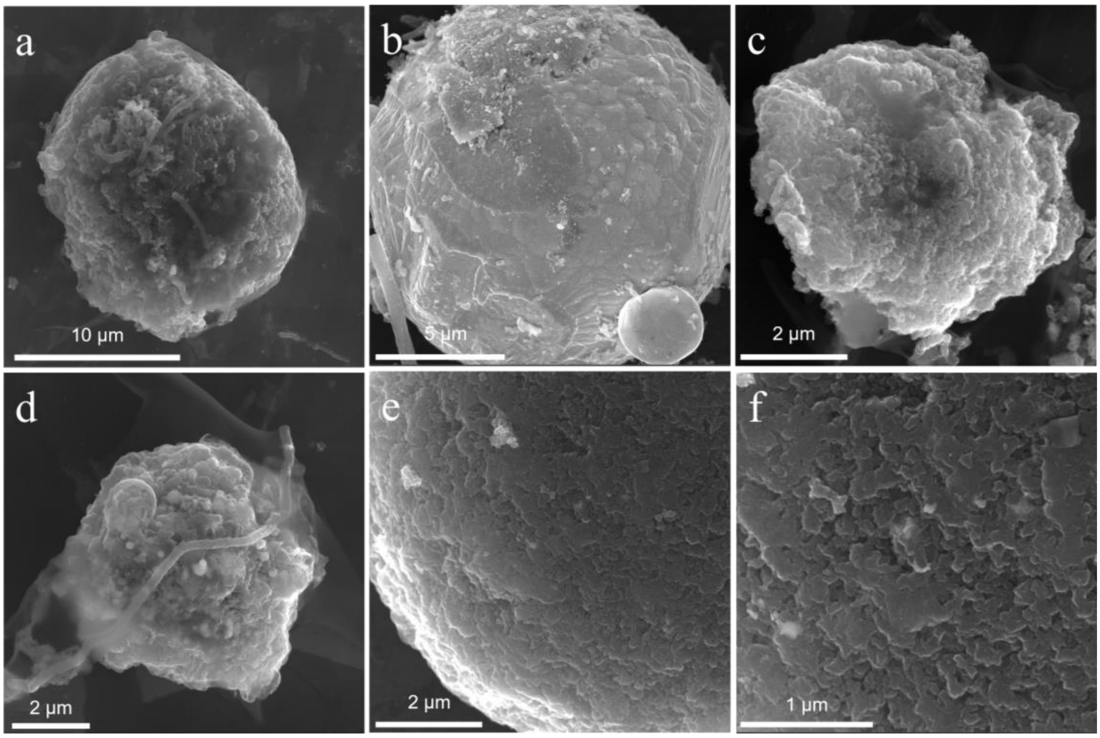

Figure S35. SEM images of solid carbon derived from CMD at 850 °C for 2 h and catalyzed by Ni_3Mn-Bi.

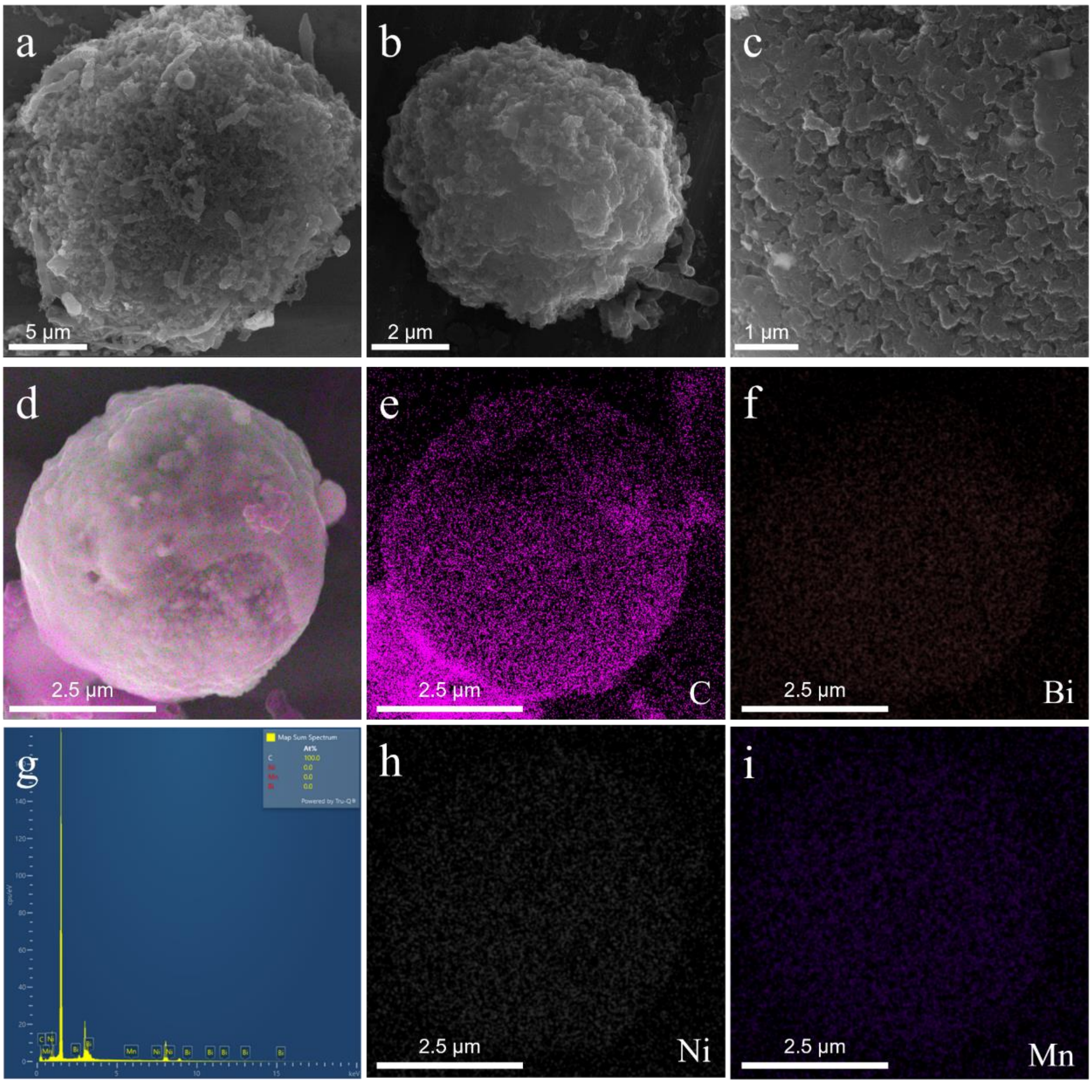

Figure S36. SEM results a-c) SEM images of solid carbon derived from CMD at 850 °C for 2 h and catalyzed by Ni_3Mn-Bi. d) EDS mapping results of (e) C; (f) Bi; (h) Ni; and (i) Mn elements. g) Energy dispersive X-ray spectroscopy (EDS).

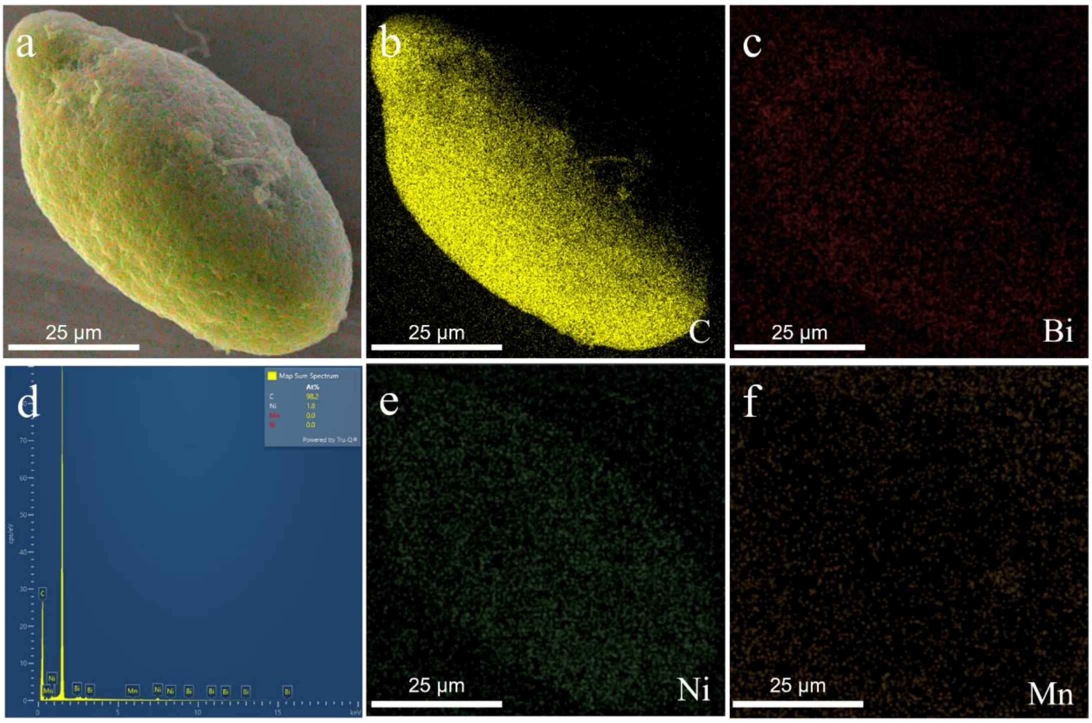

Figure S37. SEM images of solid carbon derived from CMD at 850 °C for 2 h and catalyzed by Ni_3Mn-Bi. a) EDS mapping results of (b) C; (c) Bi; (e) Ni; and (f) Mn elements. d) Energy dispersive X-ray spectroscopy (EDS).

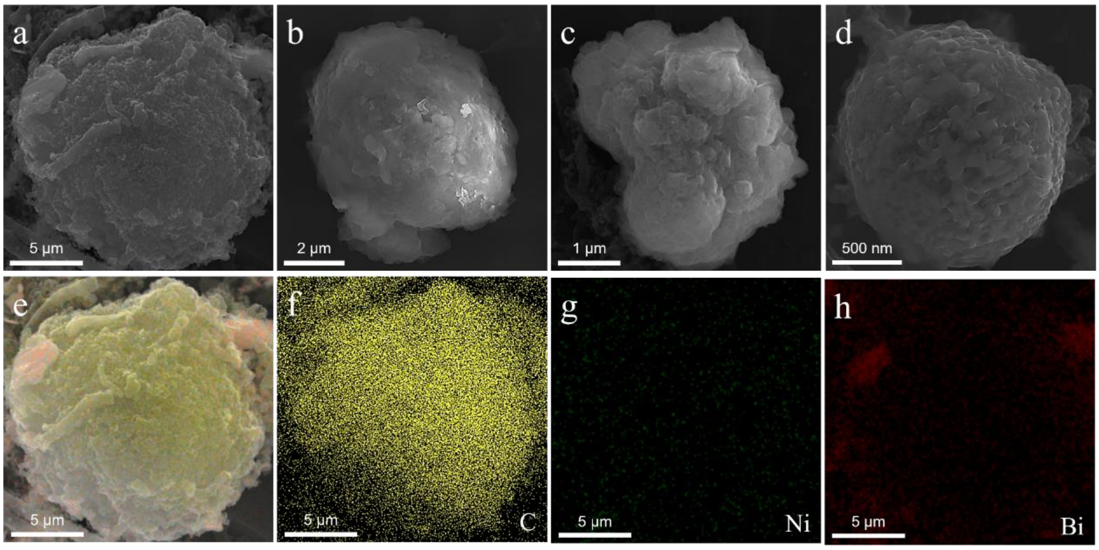

Figure S38. SEM results a-d) SEM images of solid carbon derived from CMD at 850 °C for 2 h and catalyzed by Ni-Bi. e) EDS mapping results of (f) C; (g) Ni; and (h) Bi elements.

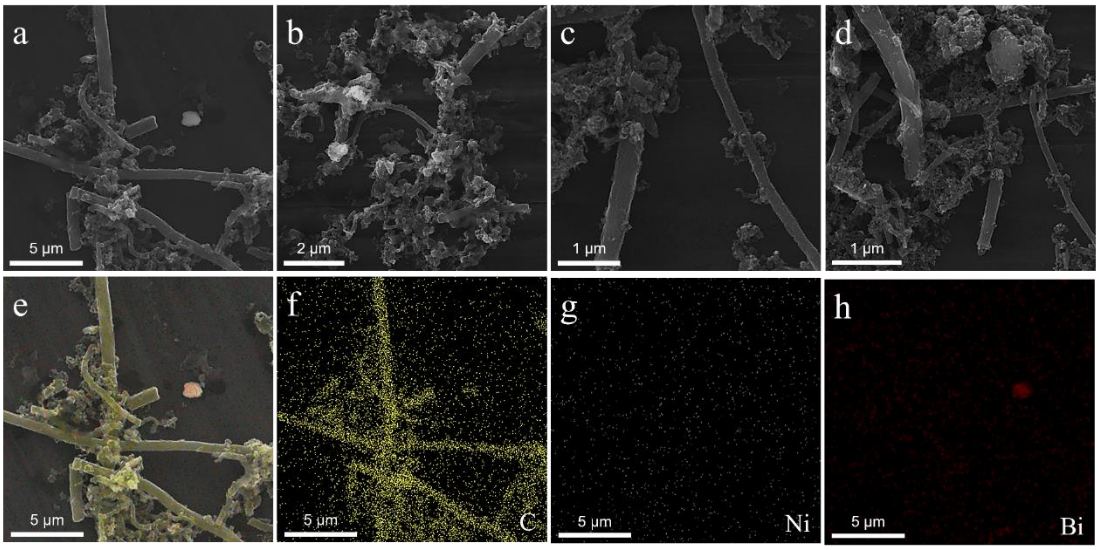

Figure S39. SEM results a-d) SEM images of carbon microtubes derived from CMD at 850 °C for 2 h and catalyzed by Ni-Bi. e) EDS mapping results of (f) C; (g) Ni; and (h) Bi elements.

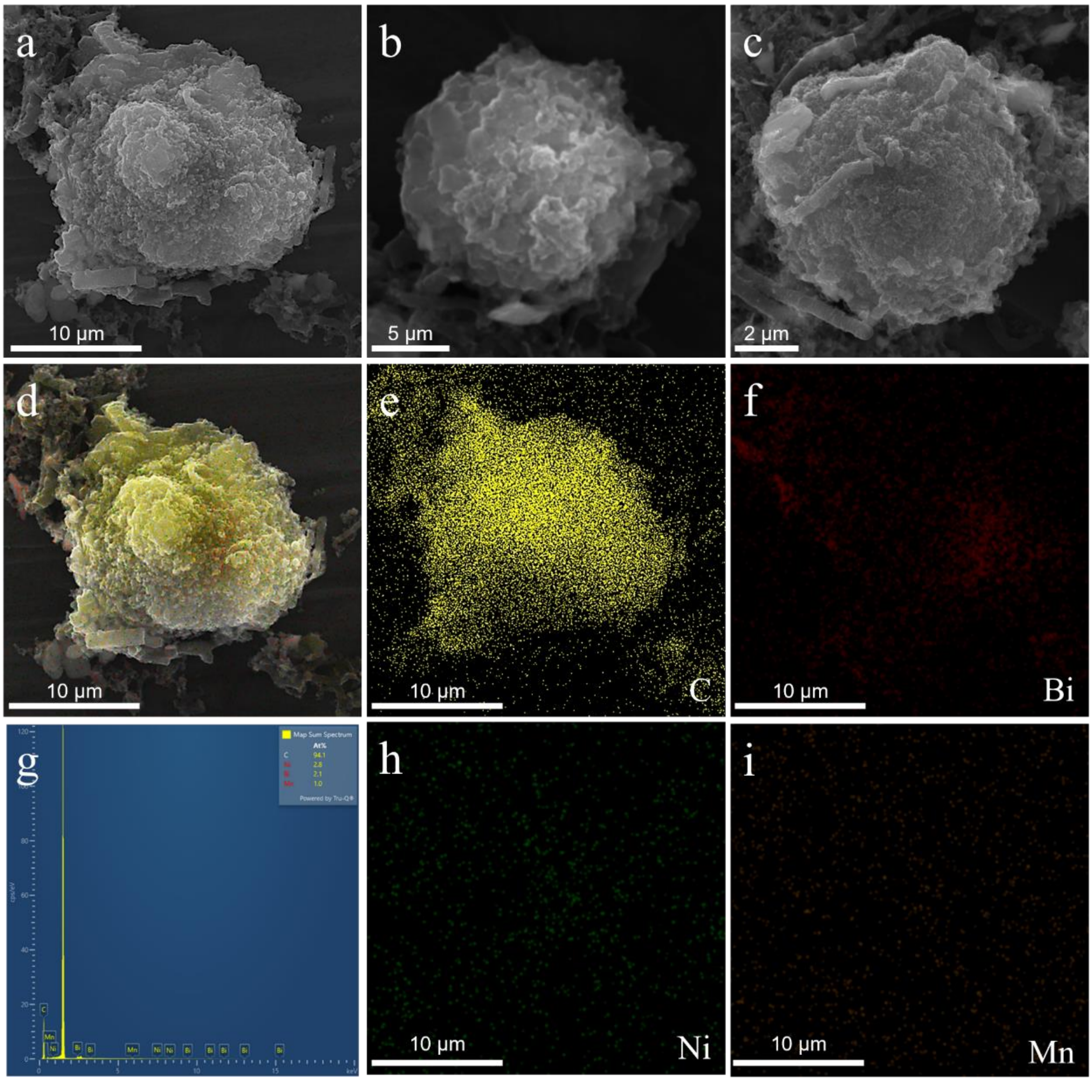

Figure S40. SEM results a-c) SEM images of solid carbon derived from CMD at 850 °C for 2 h and catalyzed by $Ni_{0.21}Mn_{0.07}$-$Bi_{0.79}$. d) EDS mapping results of (e) C; (f) Bi; (h) Ni; and (i) Mn elements. g) Energy dispersive X-ray spectroscopy (EDS).

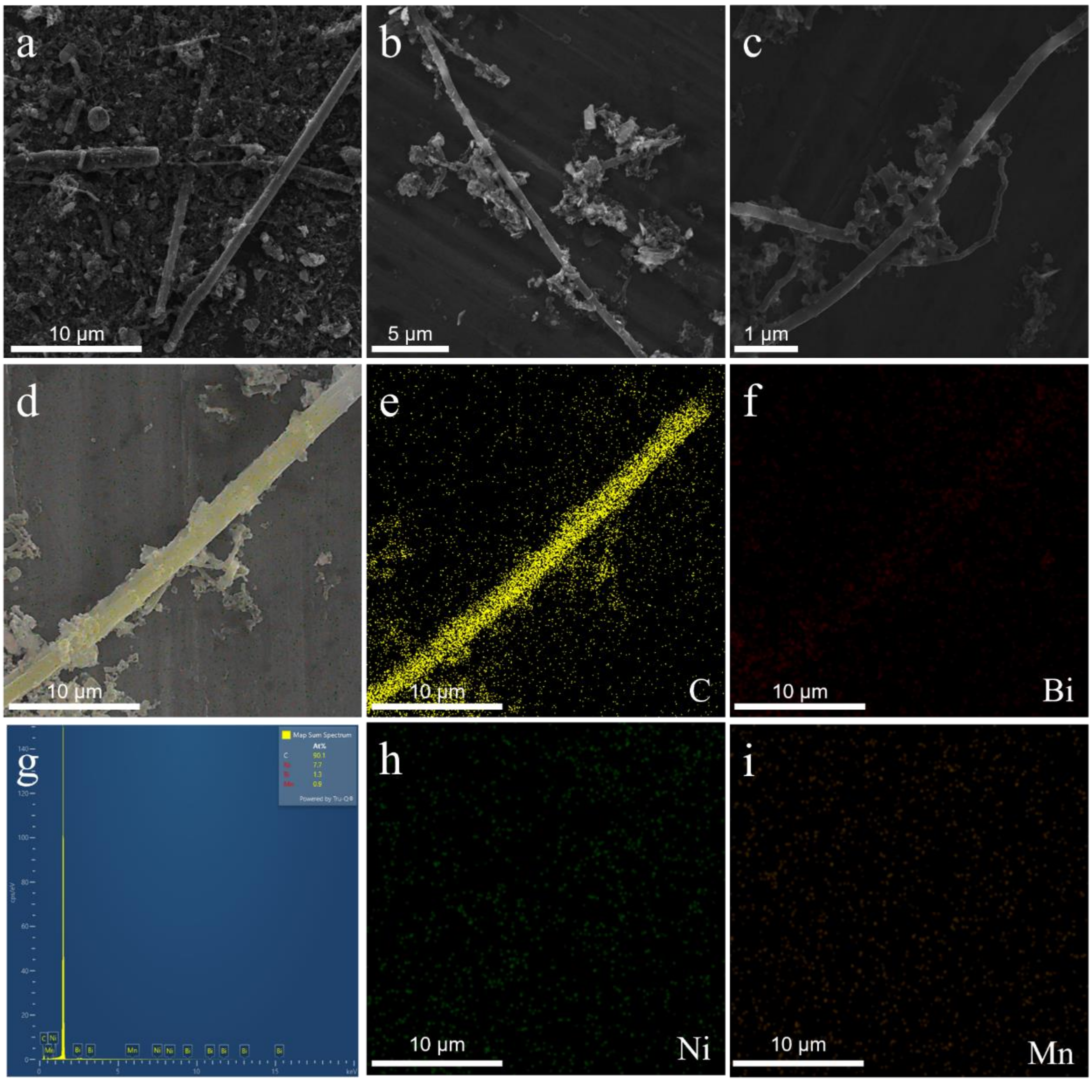

Figure S41. SEM results a-c) SEM images of carbon microtubes derived from CMD at 850 °C for 2 h and catalyzed by $Ni_{0.21}Mn_{0.07}$-$Bi_{0.79}$. d) EDS mapping results of (e) C; (f) Bi; (h) Ni; and (i) Mn elements. g) Energy dispersive X-ray spectroscopy (EDS).

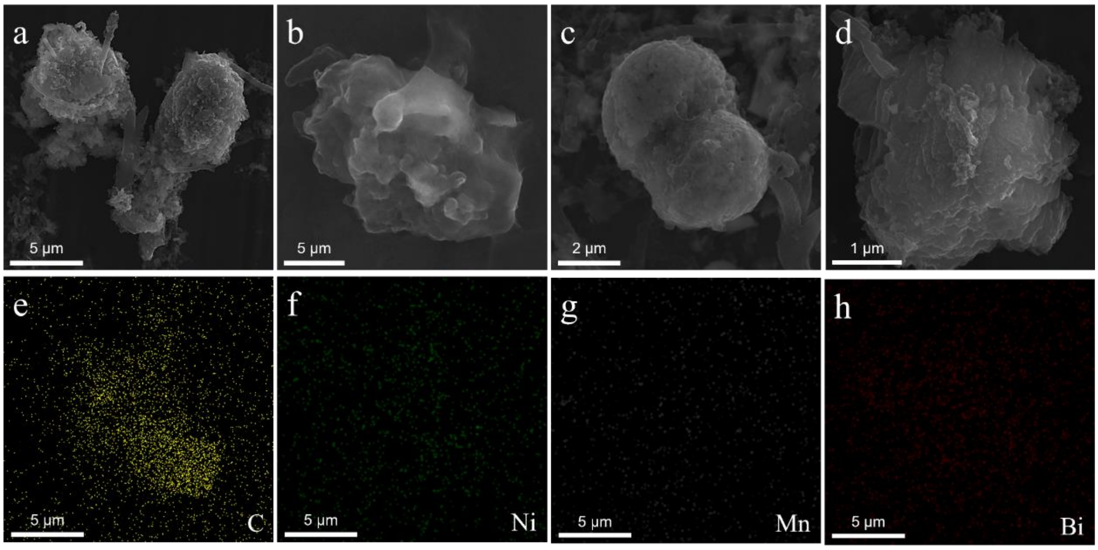

Figure S42. SEM results a-d) SEM images of solid carbon derived from CMD at 850 °C for 2 h and catalyzed by $Ni_{0.33}Mn_{0.11}$-$Bi_{0.67}$. EDS mapping results of e) C; (f) Ni; (g) Mn and (h) Bi elements.

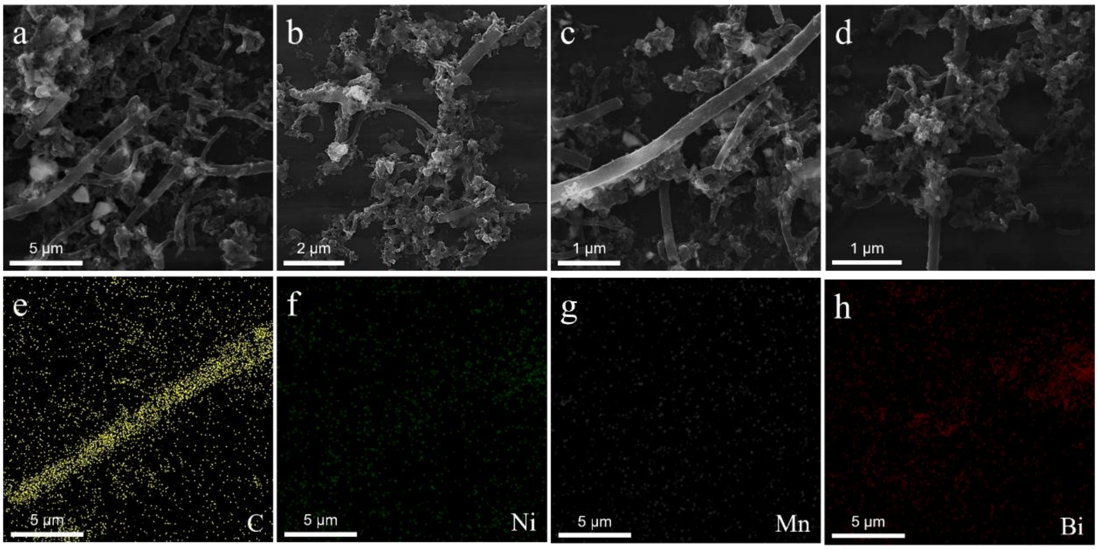

Figure S43. SEM results a-d) SEM images of carbon microtubes derived from CMD at 850 °C for 2 h and catalyzed by $Ni_{0.33}Mn_{0.11}$-$Bi_{0.67}$. EDS mapping results of e) C; (f) Ni; (g) Mn and (h) Bi elements.

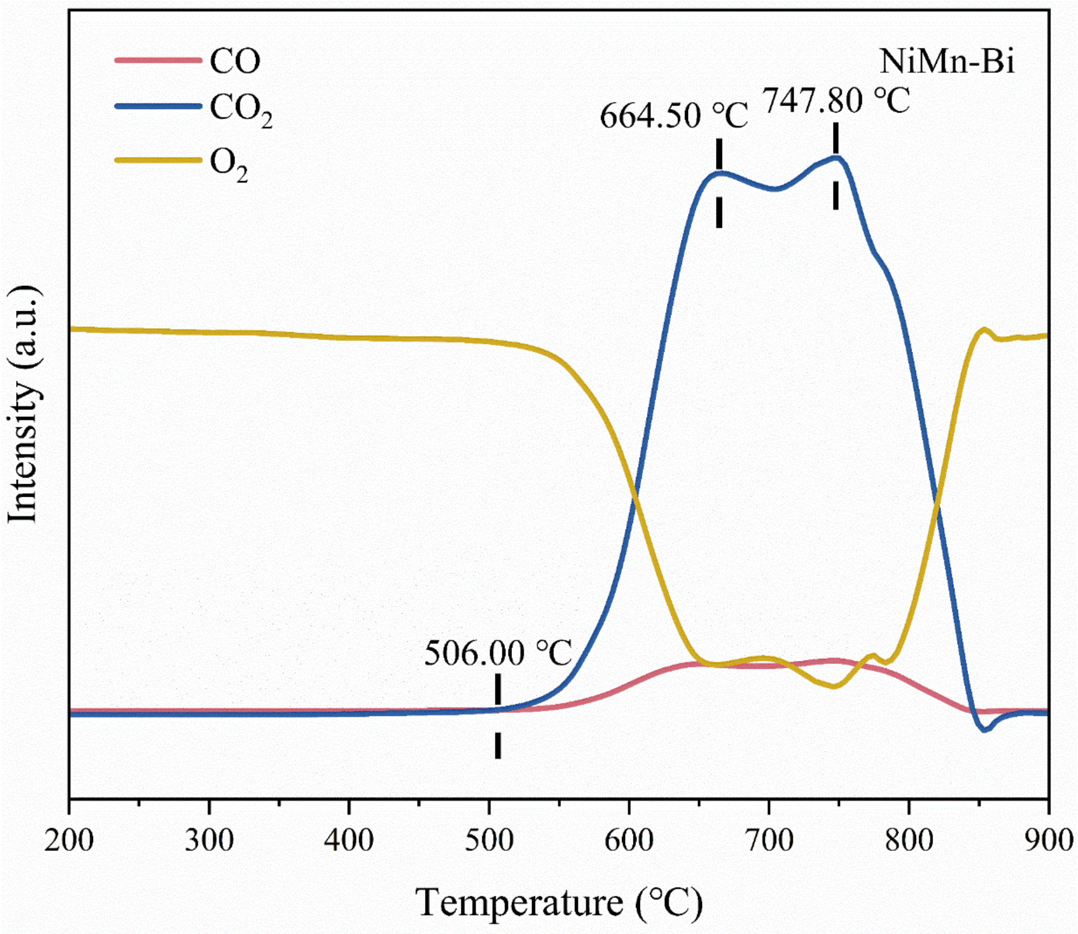

Figure S44. O_2-TPO curves of Ni_3Mn-Bi catalysts.

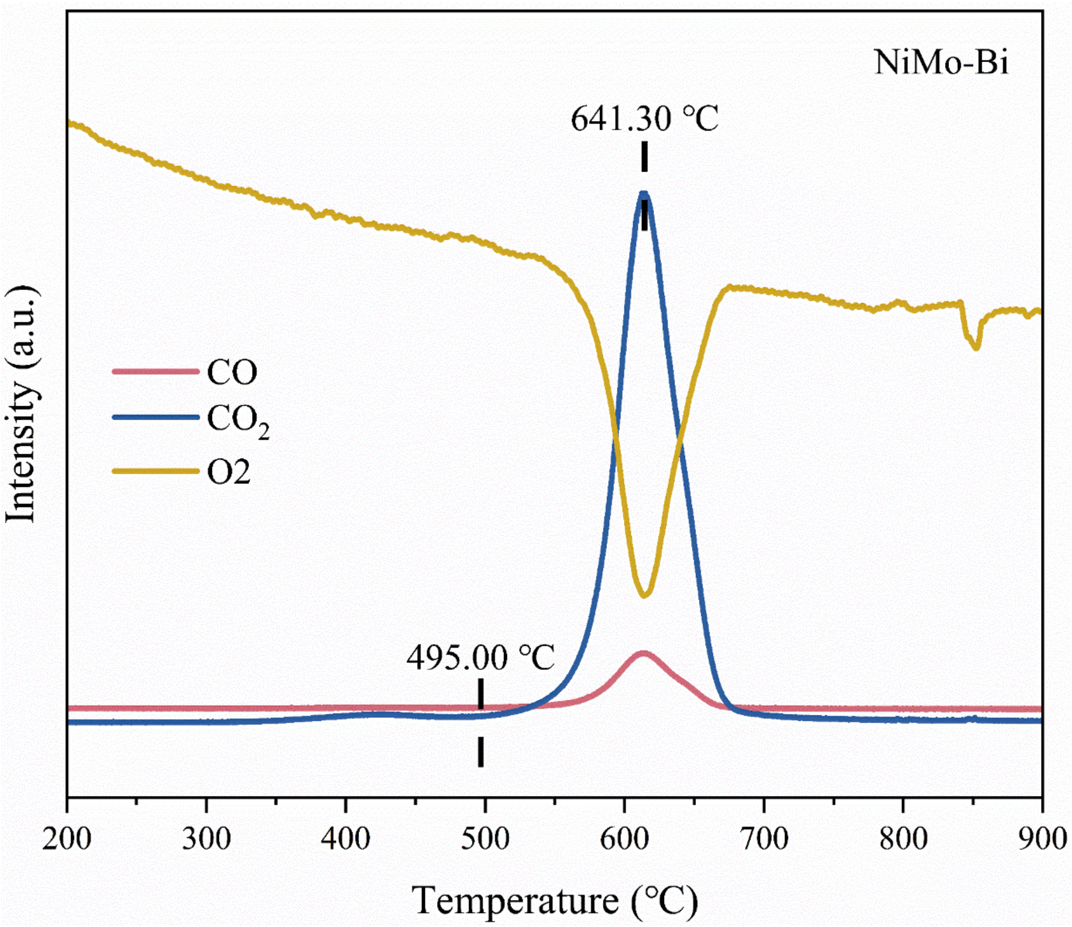

Figure S45. O_2-TPO curves of Ni_3Mo-Bi catalysts.

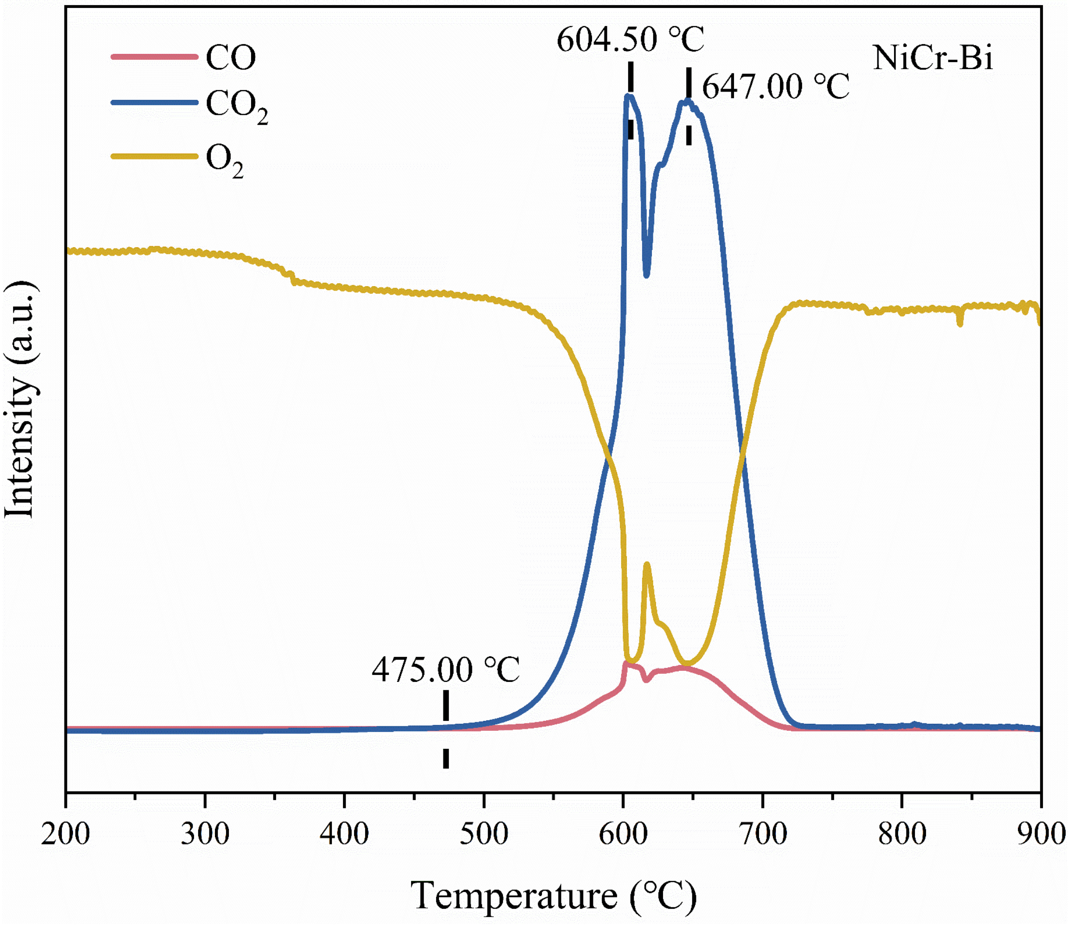

Figure S46. O_2-TPO curves of Ni_3Cr-Bi catalysts.

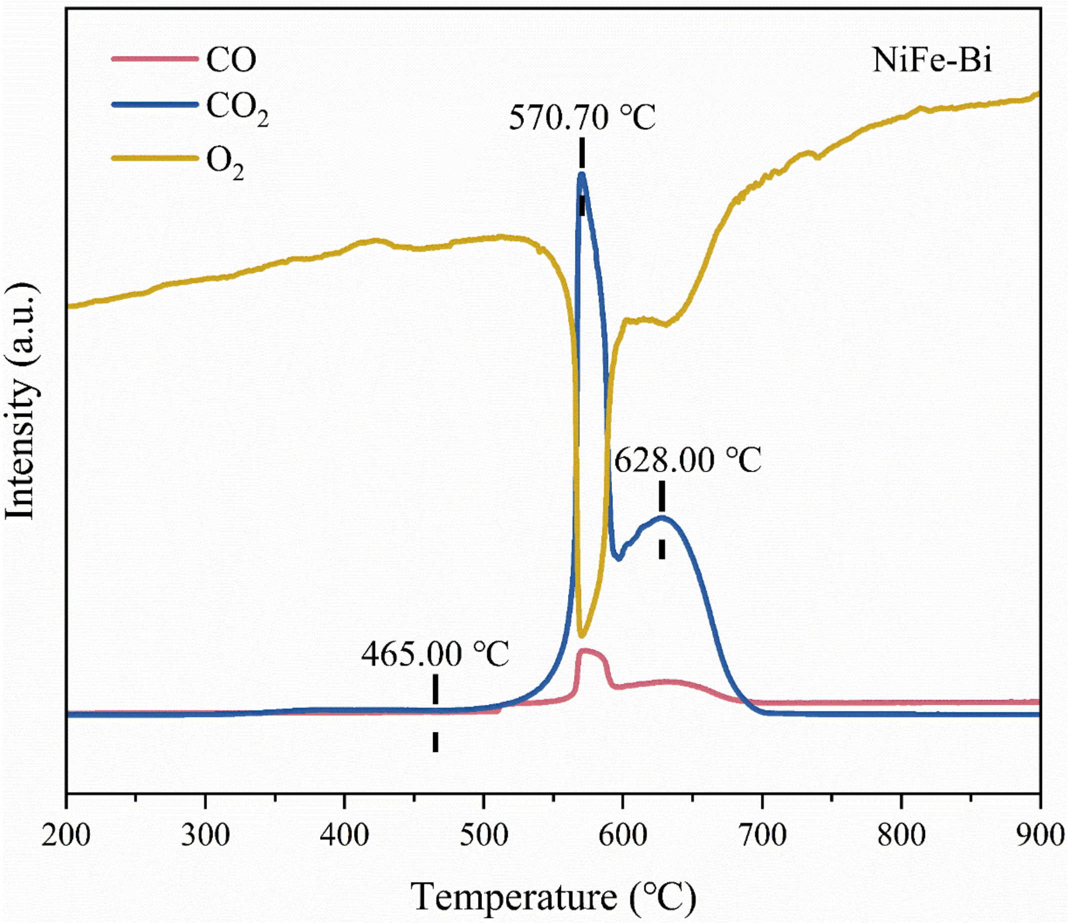

Figure S47. O_2-TPO curves of Ni_3Fe-Bi catalysts.

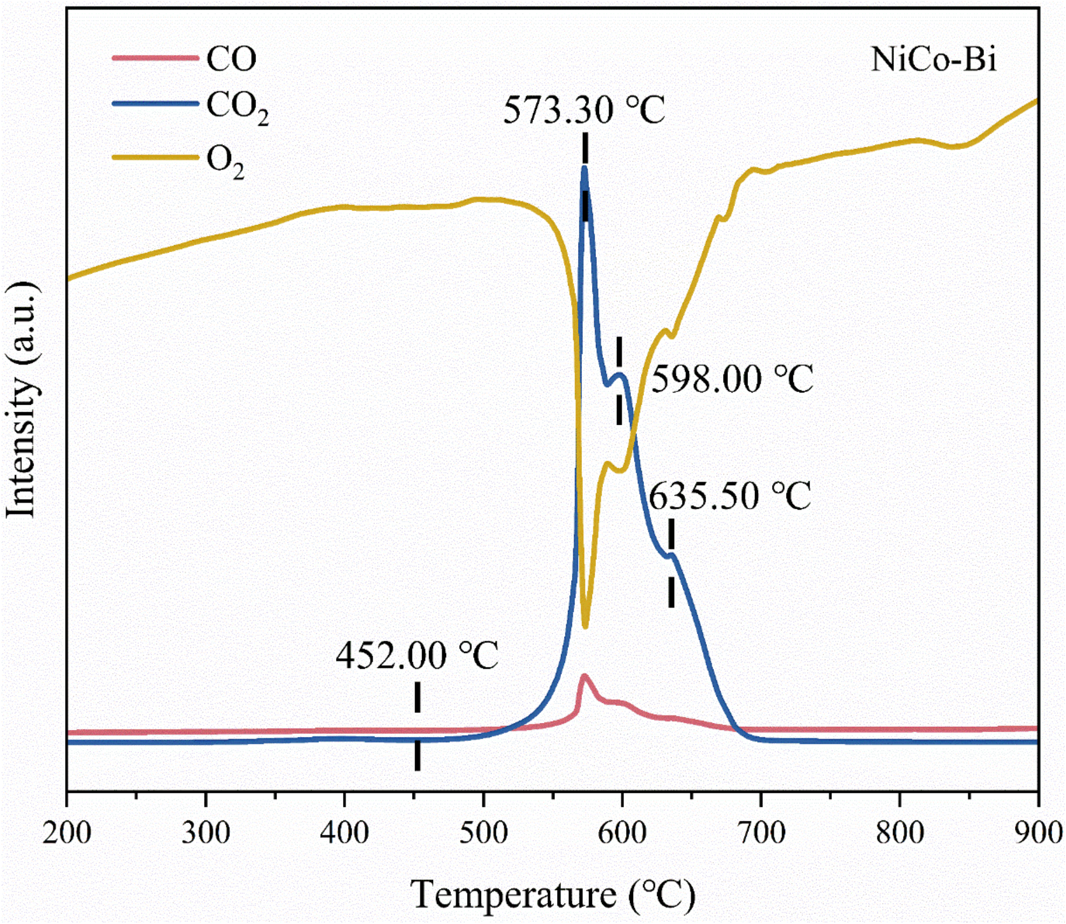

Figure S48. O_2-TPO curves of Ni_3Co-Bi catalysts.

O_2 temperature-programmed oxidation (O_2-TPO) measurements were further carried out to investigate the reactivity of different NiM-Bi derived carbon materials (Figure S44-S48). Results revealed that the carbon materials generated by Mn-doped catalysts showed both high onset and peak temperatures (506.0 and 664.4 °C), confirming the generation of highly graphitized carbon. On the contrary, the carbon catalyzed by Mo-, Cr-, Fe-, and Co-doped catalysts demonstrate much lowered oxidation temperatures.

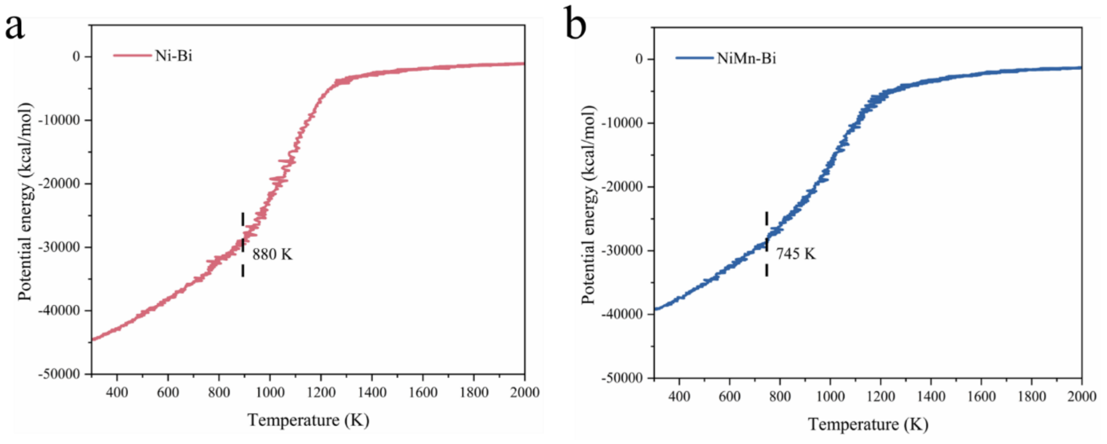

Figure S49. Temperature-potential energy curves modeling the melting point of catalysts. a) Ni-Bi catalyst. b) Ni_3Mn-Bi catalyst.

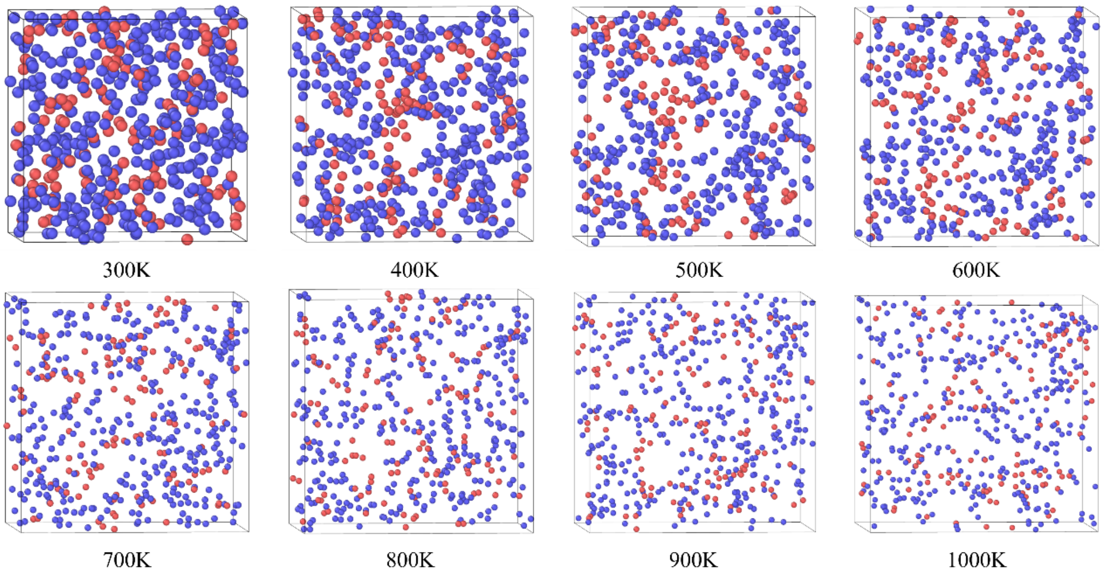

Figure S50. Molecular dynamics simulation study: melting process of Ni-Bi catalysts in the temperature range of 300-1000 K.

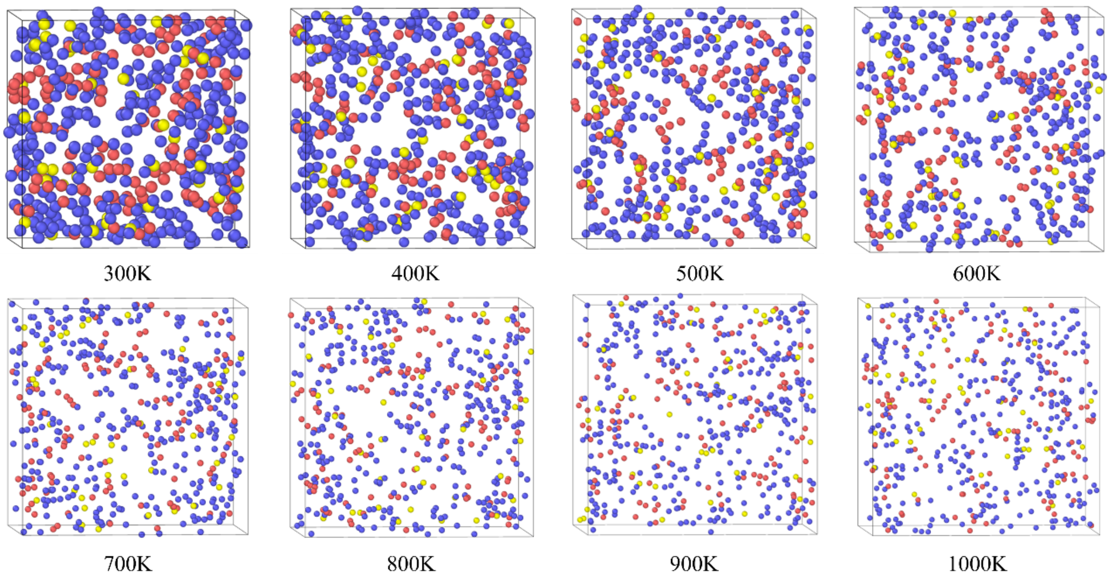

Figure S51. Molecular dynamics simulation study: melting process of Ni_3Mn-Bi catalysts in the temperature range of 300-1000 K.

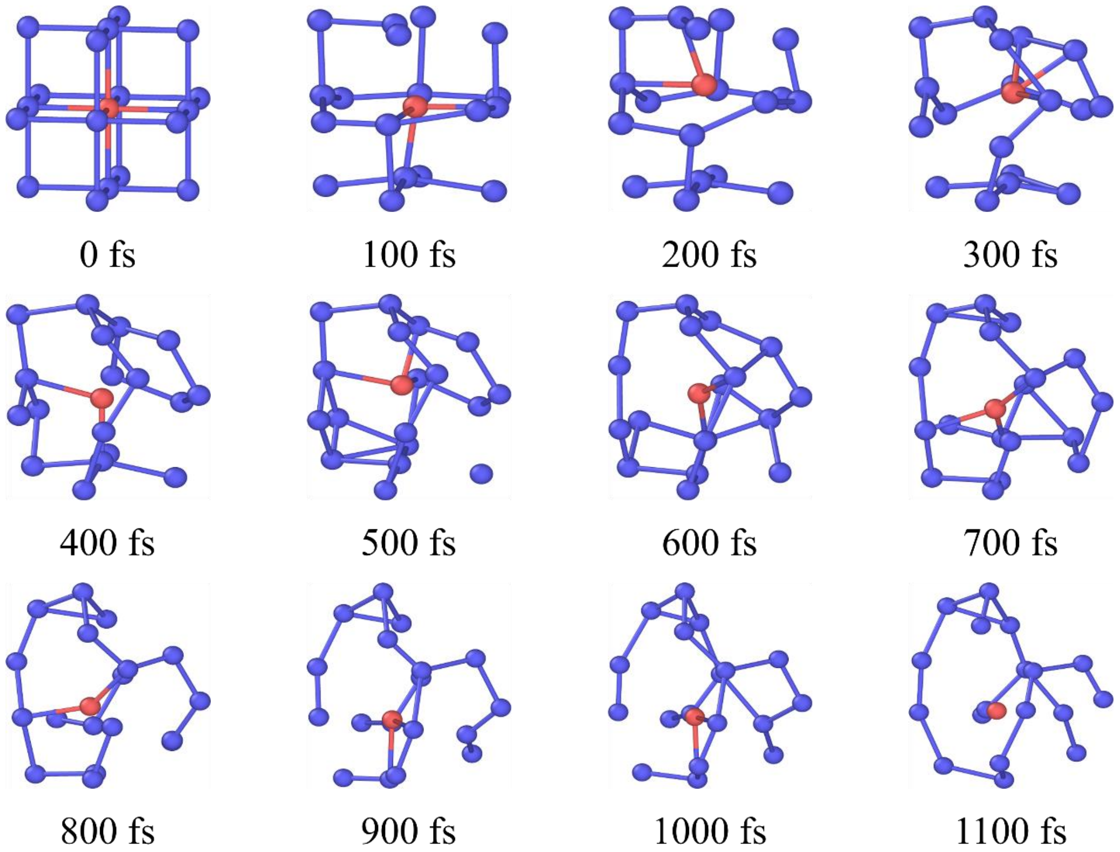

Figure S52. Simulation of bond breaking and bonding of Ni-Bi catalysts at different times at 1500 K.

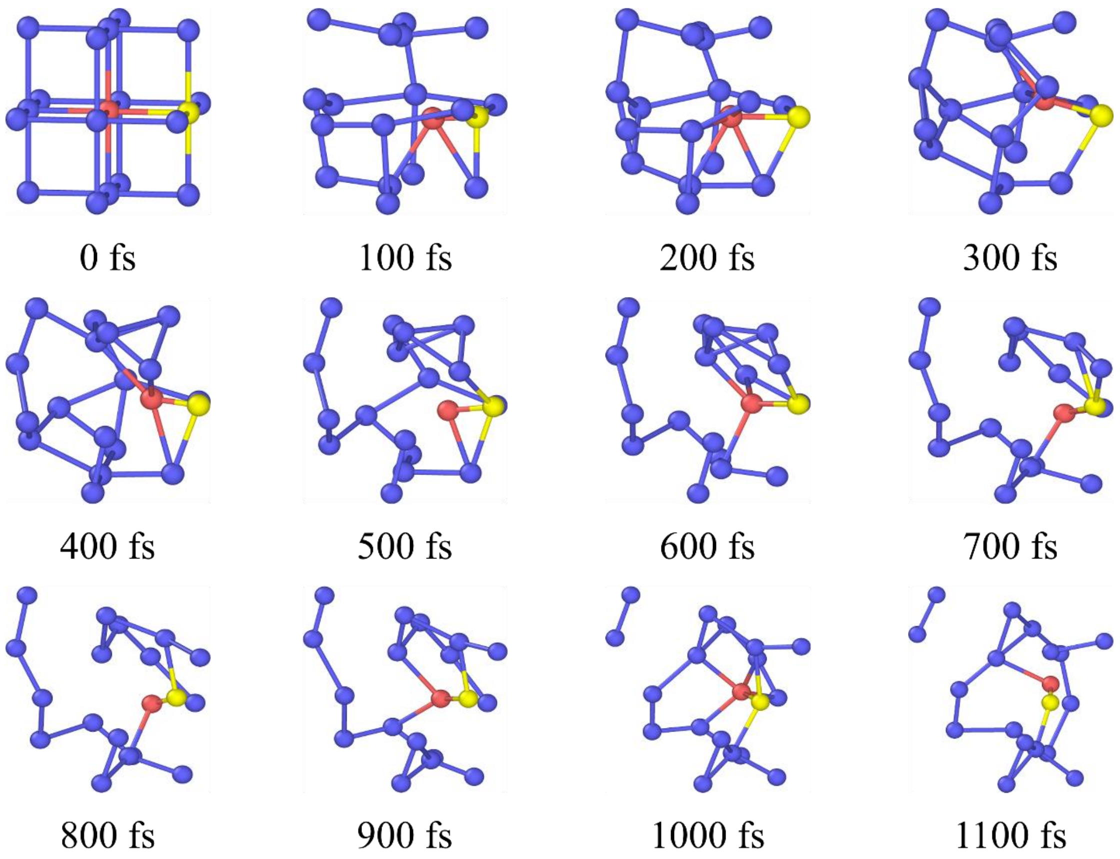

Figure S53. Simulation of bond breaking and bonding of Ni_3Mn-Bi catalysts at different times at 1500 K.

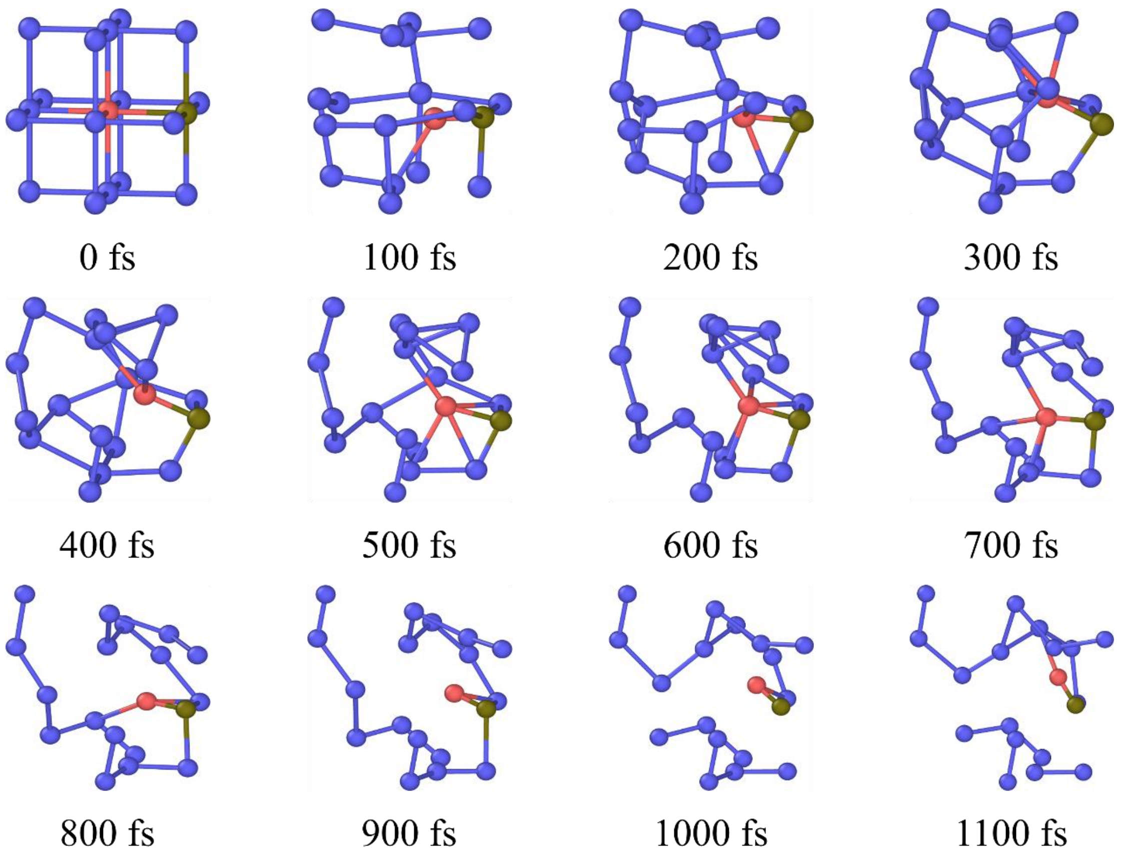

Figure S54. Simulation of bond breaking and bonding of Ni_3Co-Bi catalysts at different times at 1500 K.

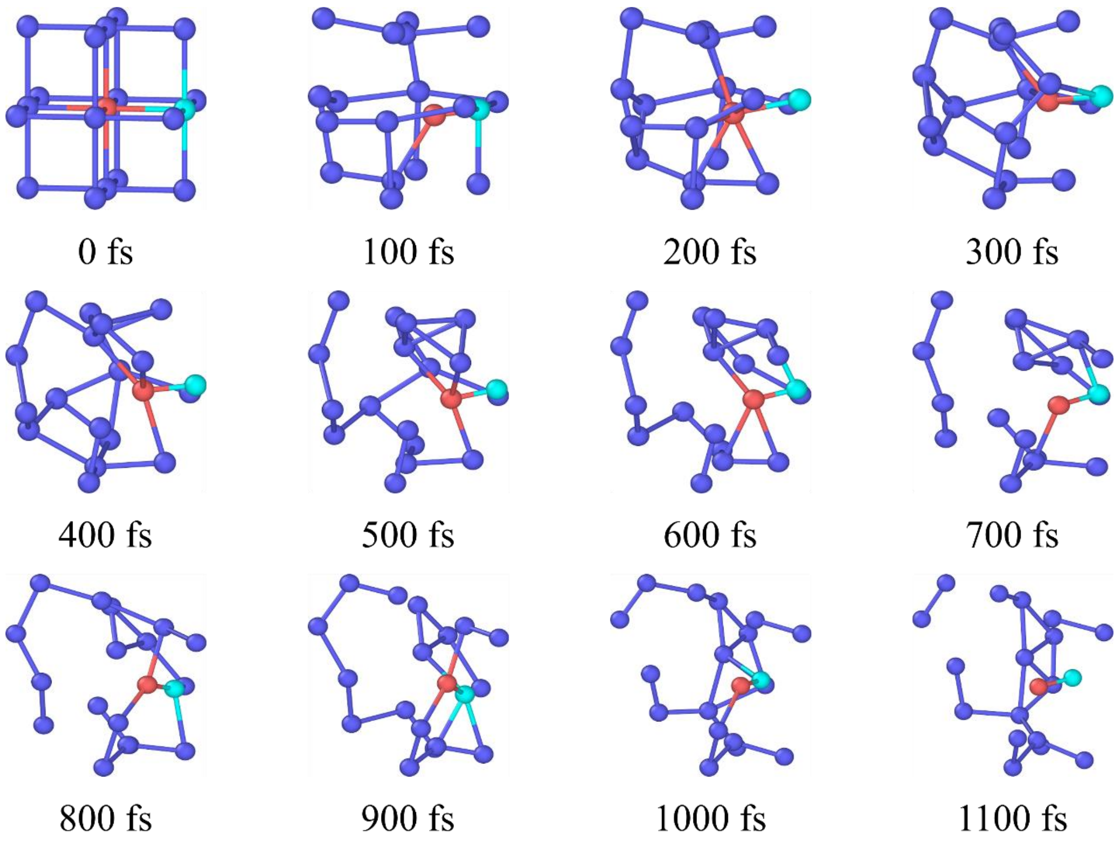

Figure S55. Simulation of bond breaking and bonding of Ni_3Fe-Bi catalysts at different times at 1500 K.

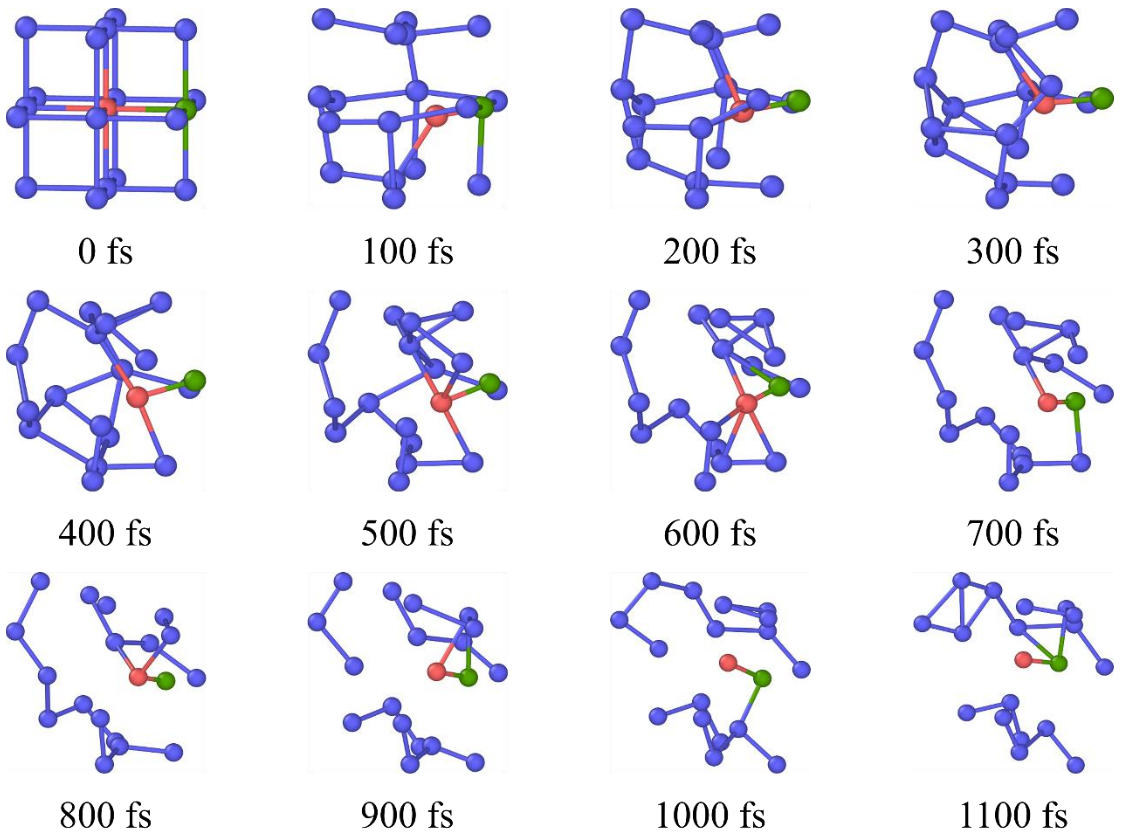

Figure S56. Simulation of bond breaking and bonding of Ni_3Mo-Bi catalysts at different times at 1500 K.

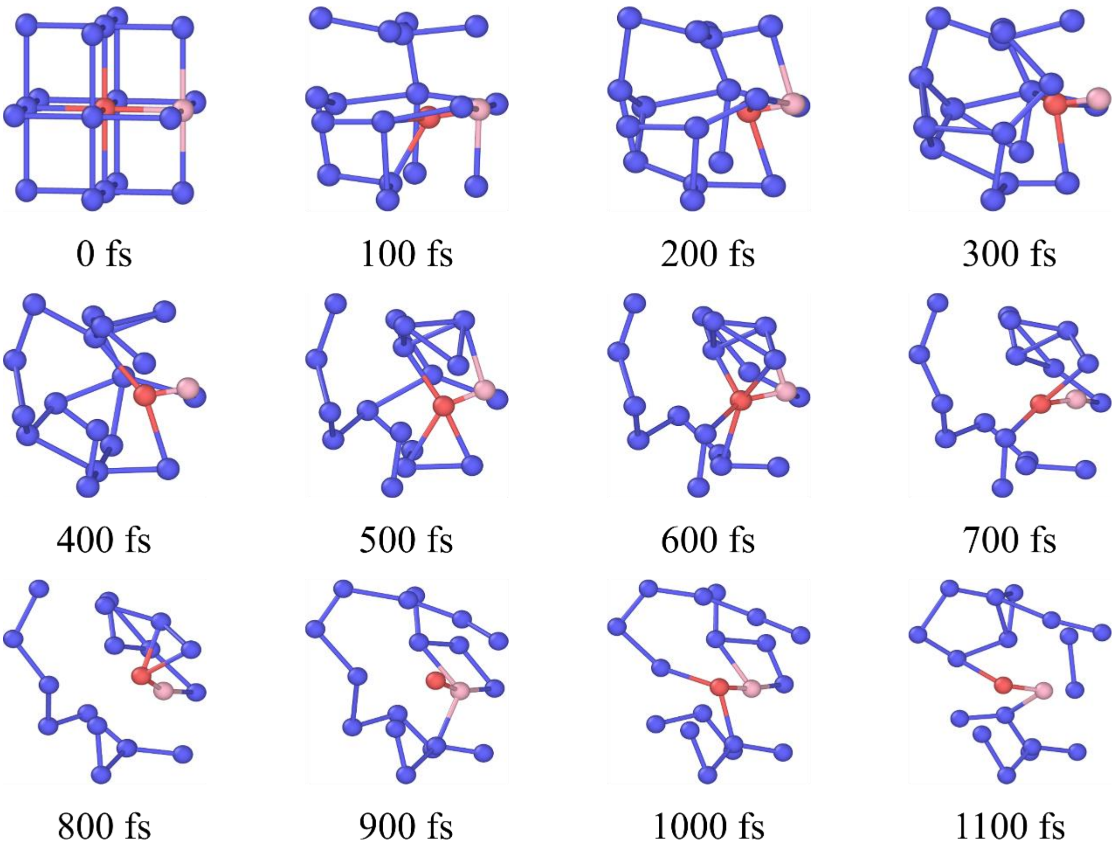

Figure S57. Simulation of bond breaking and bonding of Ni_3Cr-Bi catalysts at different times at 1500 K.

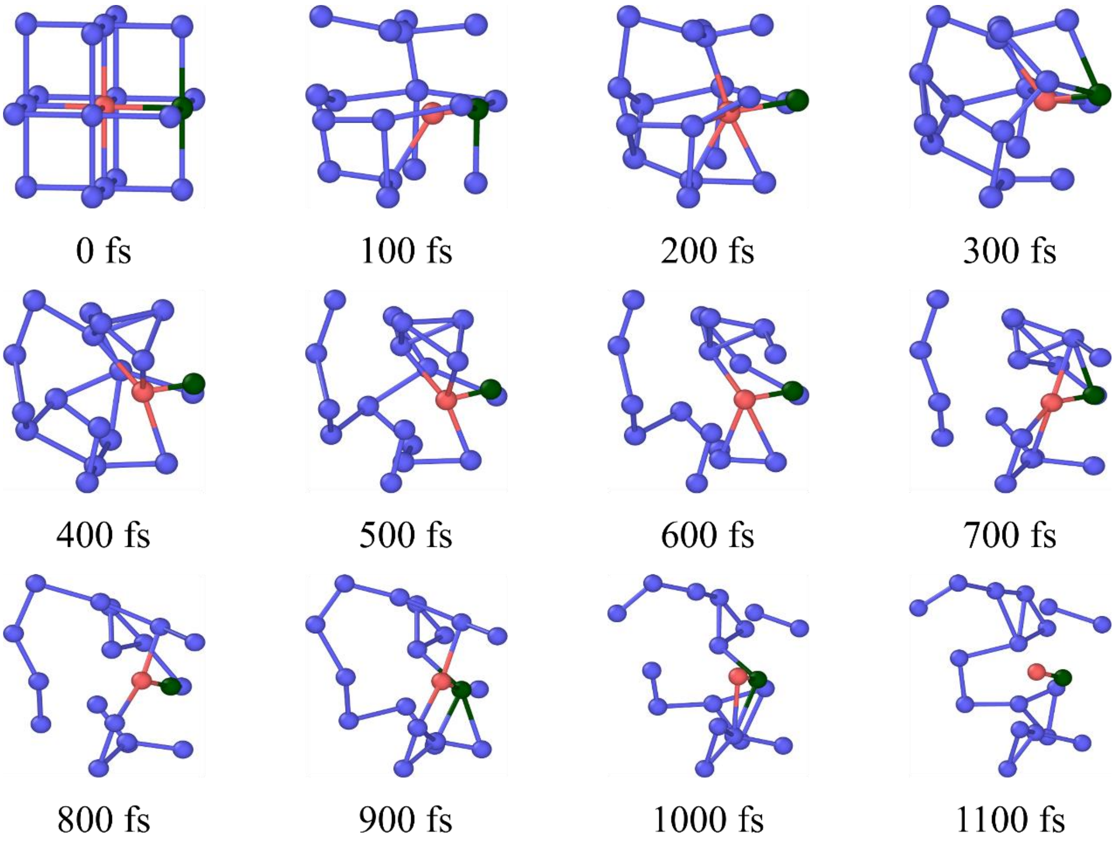

Figure S58. Simulation of bond breaking and bonding of Ni_3Cu-Bi catalysts at different times at 1500 K.

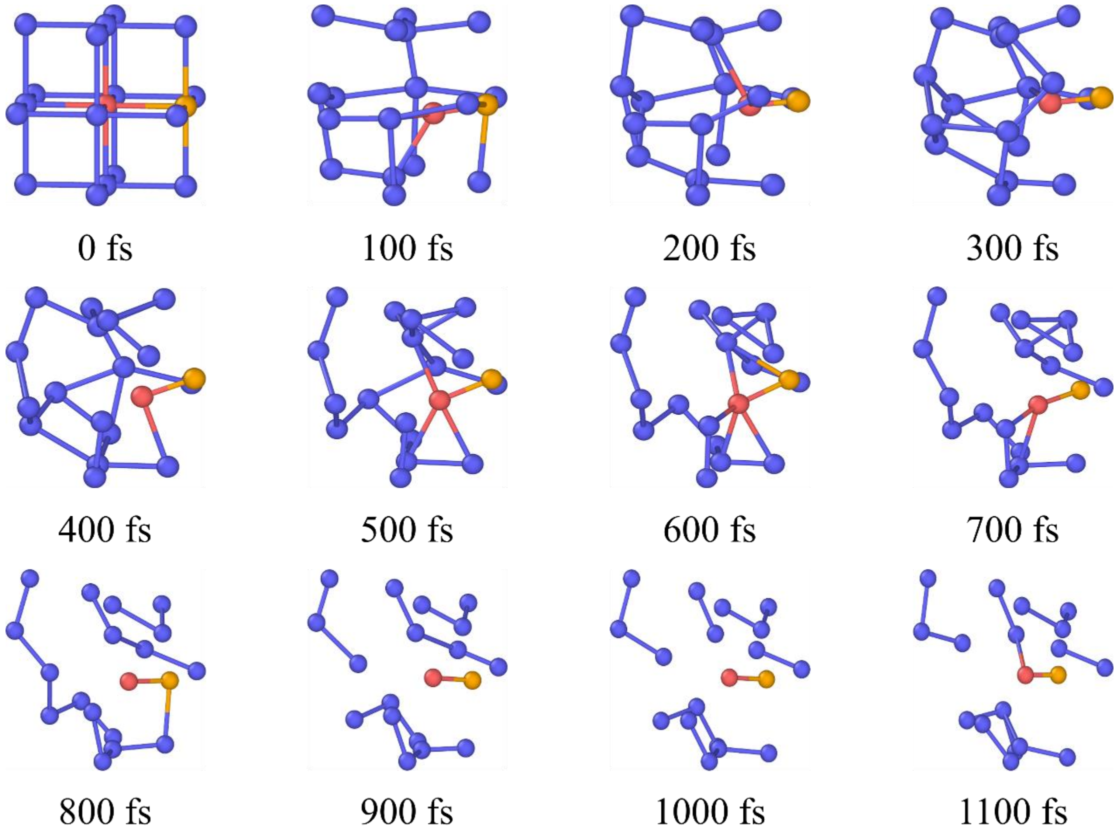

Figure S59. Simulation of bond breaking and bonding of Ni_3W-Bi catalysts at different times at 1500 K.

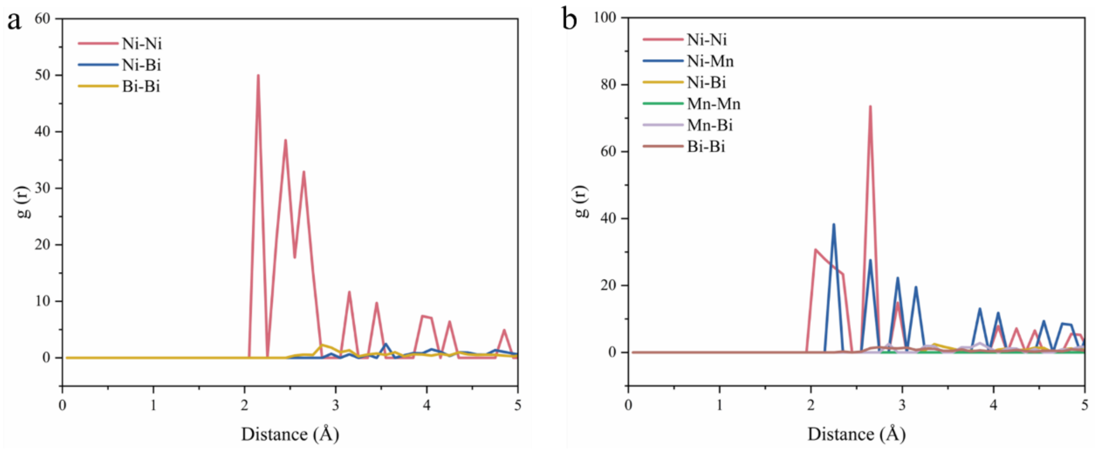

Figure S60. Molecular dynamics simulation of radial distribution function versus distance for catalysts. a) Ni-Bi catalysts. b) Ni_3Mn-Bi catalysts.

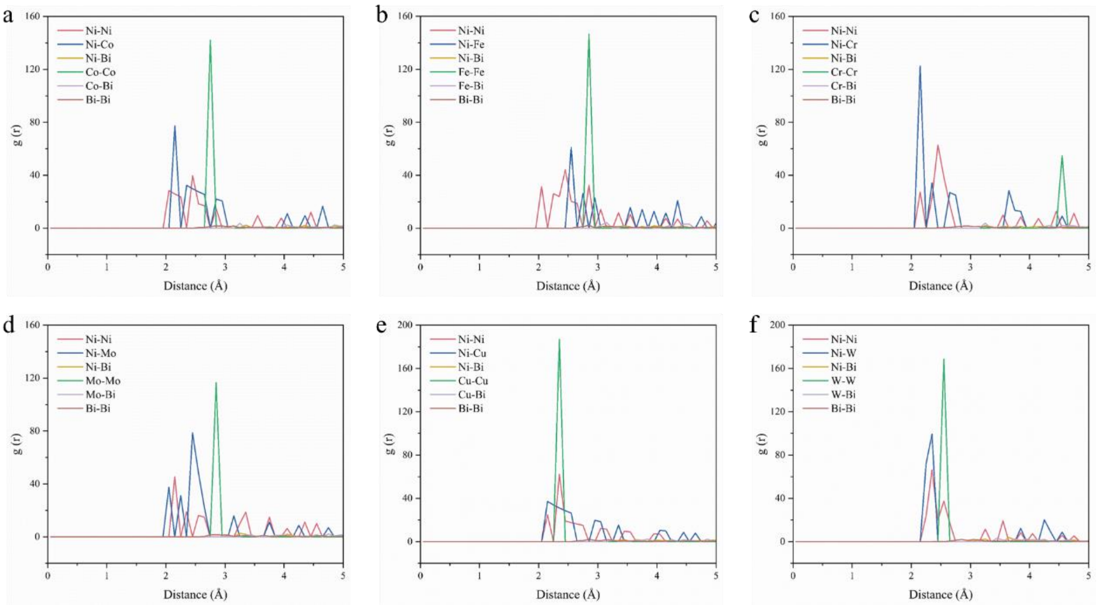

Figure S61. Molecular dynamics simulation of radial distribution function versus distance for different doping elements catalysts. a) Ni_3Co-Bi catalysts. b) Ni_3Fe-Bi catalysts. c) Ni_3Cr-Bi catalysts. d) Ni_3Mo-Bi catalysts. e) Ni_3Cu-Bi catalysts. f) Ni_3W-Bi catalysts.

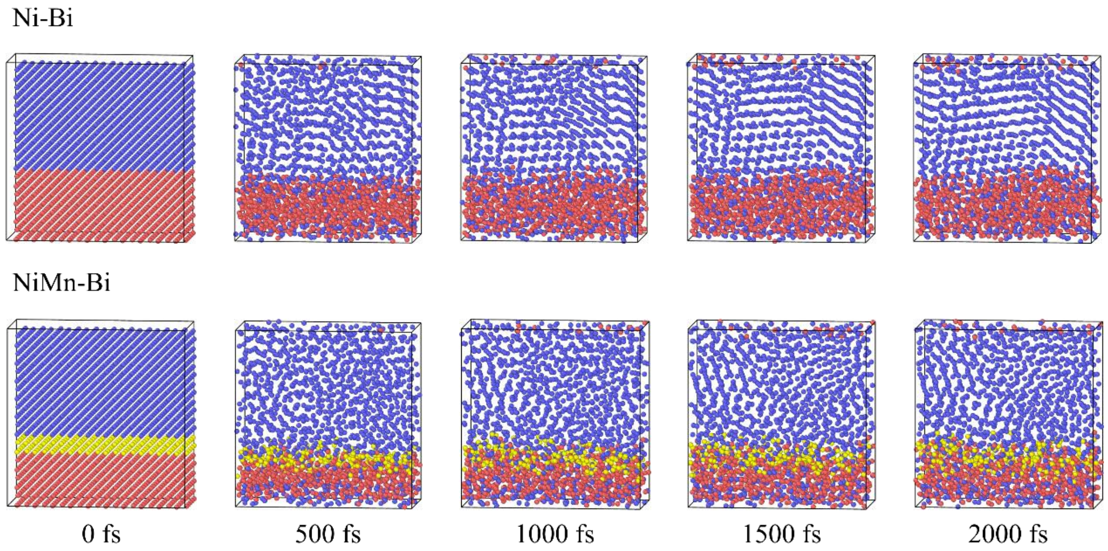

Figure S62. Molecular dynamics simulation of the evolution of Ni-Bi interactions in Ni-Bi and Ni_3Mn-Bi catalysts at 1500 K. Different colors represent different atomic species, red for Ni atoms, blue and yellow for Bi and doped Mn atoms, respectively.

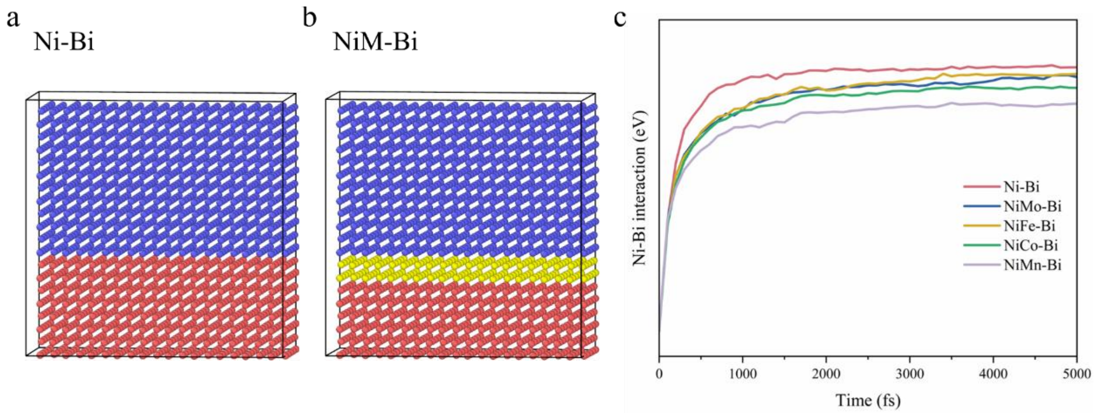

Figure S63. Initial modeling for molecular dynamics simulations of the interaction between Ni-Bi. a) Ni-Bi catalysts. b) Ni_3Mn-Bi catalysts. c) Evolution of the Ni-Bi interaction in Ni-Bi and NiM-Bi (M=Mo， Fe, Co, Mn) catalysts as a function of simulation duration.

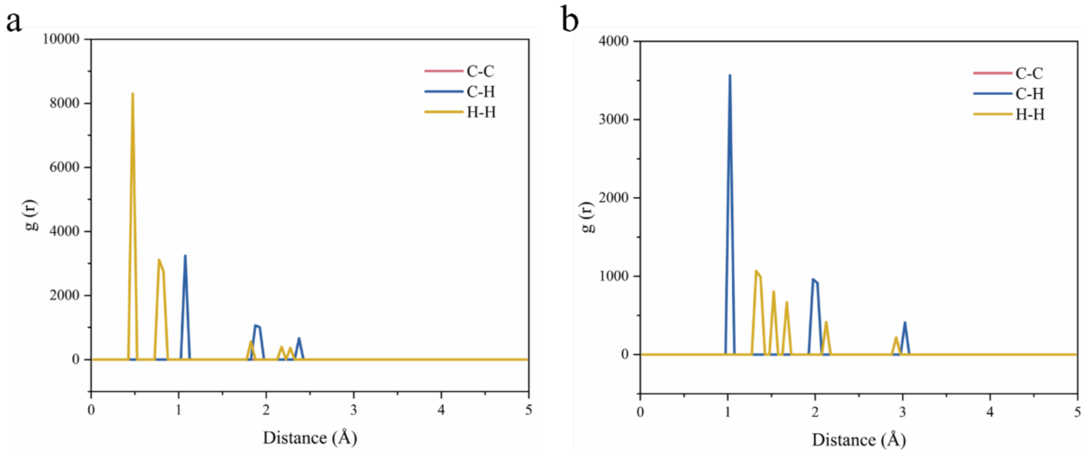

Figure S64. Simulation of RDF in methane decomposition reaction: a) CH_4 molecule in Ni-Bi catalyst. b) CH_4 molecule in NiMn-Bi catalyst.

Figure S64 shows that the CH_4 molecules in the Ni-Bi catalysts exhibit significant RDF peaks of C-H and H-H bonds, indicating that there are strong interactions of C-H bonds in CH_4 molecules in the short range of distances from 0-1.0 Å. The RDF peaks are more pronounced on the NiMn-Bi catalysts than on the NiMn-Bi catalysts. On NiMn-Bi catalysts, the RDF peaks of C-H bonds are more significant, while the H-H bonds are mainly distributed in a more distant distance range. It is shown that the introduction of Mn facilitates the breaking of C-H bonds and significantly enhances the adsorption and activation of C-H bonds by the catalyst.

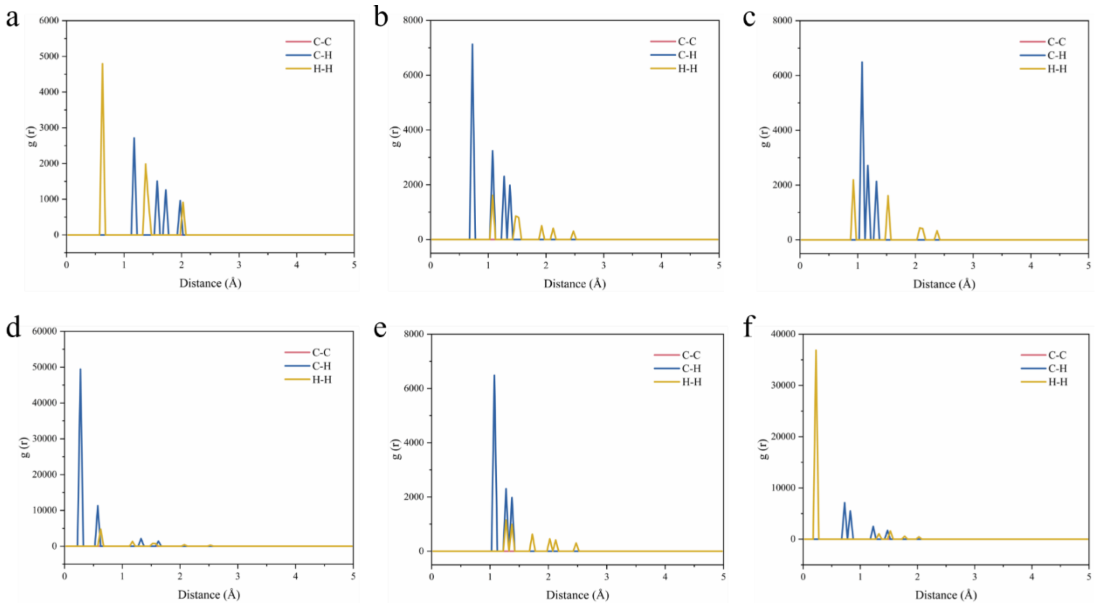

Figure S65. Simulation of RDF in methane decomposition reaction: CH_4 molecule in a) Ni_3Co-Bi catalysts. b) Ni_3Fe-Bi catalysts. c) Ni_3Cr-Bi catalysts. d) Ni_3Mo-Bi catalysts. e) Ni_3Cu-Bi catalysts. f) Ni_3W-Bi catalysts.

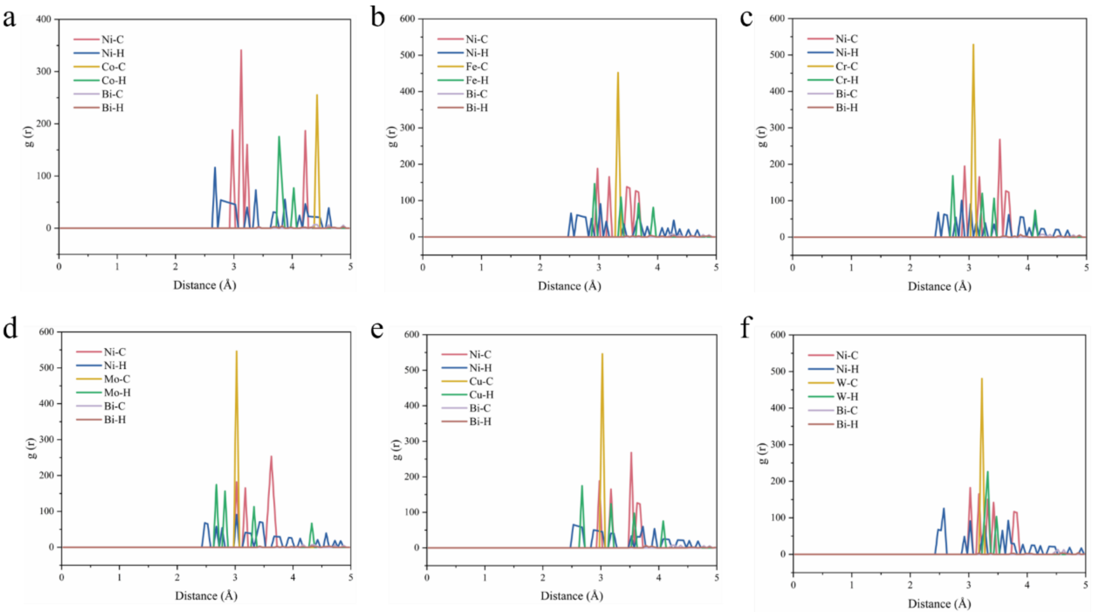

Figure S66. Simulation of RDF in methane decomposition reaction: a) Ni_3Co-Bi catalysts. b) Ni_3Fe-Bi catalysts. c) Ni_3Cr-Bi catalysts. d) Ni_3Mo-Bi catalysts. e) Ni_3Cu-Bi catalysts. f) Ni_3W-Bi catalysts.

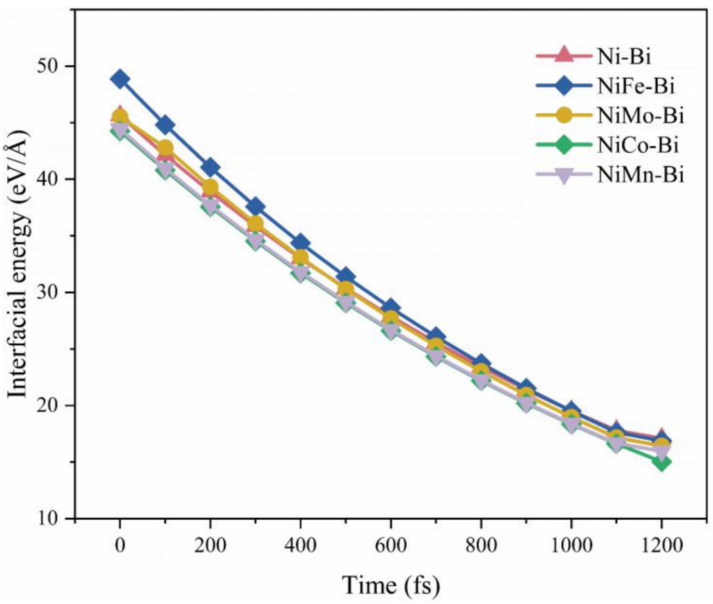

Figure S67. Interfacial energy of Ni-Bi and NiM-Bi (M=Fe, Mo, Co, Mn) catalysts.

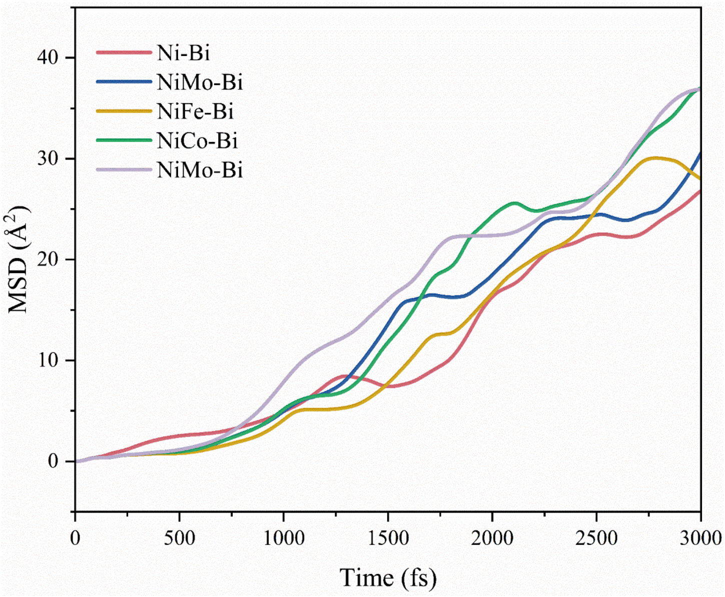

Figure S68. Mean square displacement (MSD) of CH_4 molecules in Ni-Bi and NiM-Bi (M=Fe, Mo, Co, Mn) liquid alloys.

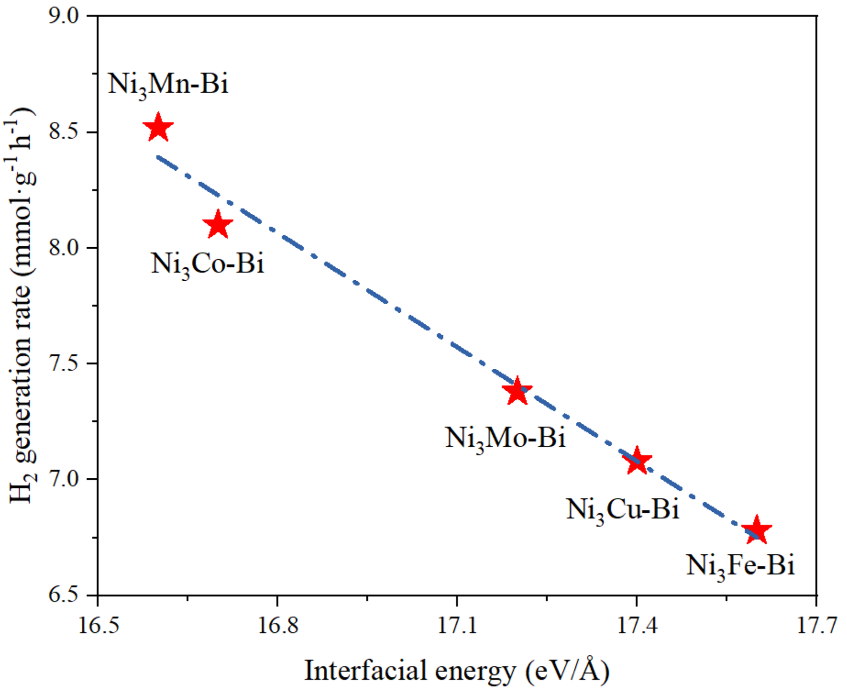

Figure S69. Verification of interfacial energy as a reliable descriptor for performance prediction.

Table S1. Reaction kinetics for CH_4 decomposition in NiMn-Bi.

Catalyst	Apparent activity energy (kJ/mol)	Pre-exponential factor (mmol/(g·h))	R^2
Ni-Bi	208.0	1.08×10^9	0.995
Ni_6Mn-Bi	117.3	1.19×10^8	0.990
Ni_4Mn-Bi	116.6	1.23×10^8	0.993
Ni_3Mn-Bi	112.9	9.75×10^7	0.996
Ni_2Mn-Bi	115.9	1.32×10^8	0.996
Ni_3Mn_2-Bi	116.6	1.34×10^8	0.994
NiMn-Bi	124.0	3.29×10^8	0.992
$NiMn_2$-Bi	115.8	1.09×10^8	0.991

Table S2. Methane conversion of catalyst under different reaction conditions.

Catalyst	Reaction temperature (°C)	Methane flow rate (mL/min)	Catalytic bed height (cm)	Methane conversion (%)
Te	900	7	7	8.0
Ni-Bi	1065	10	110	95.0
Co-Mn	1000	15	19	10.4
Ni-Bi+NaBr	985	6	9.6	18.6
NaBr-KBr	1000	15	5.9	18.0
$MnCl_2$-KCl	1050	10	12.5	43.0
NaCl-KCl	1000	10	12.5	2.8
$FeCl_3$-NaCl-KCl	1000	10	12.5	9.2
NiMo-Bi	800	4	1.0	9.9
NiMn-Bi	800	10	3.5	11.00
NiMn-Bi	850	10	3.5	15.4
NiMn-Bi	900	10	3.5	23.0
NiMn-Bi	950	10	3.5	28.8
NiMn-Bi	1000	10	3.5	42.3

Table S3. Structural properties of Ni_3M-Bi (M = Mn, Mo, Fe, Cr, Co) catalysts with different doping elements.

Catalyst	S_{BET} (m^2/g)	Pore diameter (nm)	Pore volume (cm^3/g)
Ni_3Mn-Bi	20.7	49.0	0.253
Ni_3Mo-Bi	5.9	35.7	0.052
Ni_3Fe-Bi	22.2	16.8	0.093
Ni_3Cr-Bi	11.8	20.00	0.059
Ni_3Co-Bi	11.8	23.8	0.070

Table S4. Structural properties of NiMn-Bi catalysts with different Ni-Mn molar ratios.

Catalyst	S_{BET} (m^2/g)	Pore size (nm)	Pore volume (cm^3/g)
Ni_6Mn-Bi	9.5	58.3	0.022
Ni_3Mn-Bi	20.7	49.0	0.253
Ni_2Mn-Bi	11.0	36.0	0.027
$NiMn_2$-Bi	8.1	30.3	0.009

■ REFERENCES

(1) Qingqing, P.; Yongwen, T.; Huajuan, L.; Zhenyuan, W.; Zhanglei, Z.; Rongli, J.; Yuemin, Z.; Yuelun, W.; Chen, J.; Xiaozhou, L., Tuning Hydrogen and Carbon Nanotube Production from Phenol Steam Reforming on Ni/Fe-Based Nanocatalysts. *ACS Publications* **2017,** *5* (3): 2098-2108.

(2) Rastegarpanah, A.; Rezaei, M.; Meshkani, F.; Zhang, K.; Zhao, X.; Pei, W.; Liu, Y.; Deng, J.; Arandiyan, H.; Dai, H., Influence of group VIB metals on activity of the Ni/MgO catalysts for methane decomposition. *Applied Catalysis B: Environmental* **2019,** *248*, 515-525.

(3) Saito, R.; Grueneis, A.; Cancado, L.; Pimenta, M.; Jorio, A.; Dresselhaus, G.; Dresselhaus, M.; Souza Filho, A. G.; Crystals, L., Double resonance raman spectrain disordered graphite and singlewall carbon nanotubes. *Molecular Crystals and Liquid Crystals* **2002,** *387* (1), 63-72.

(4) Pimenta, M.; Dresselhaus, G.; Dresselhaus, M. S.; Cancado, L.; Jorio, A.; Saito, R., Studying disorder in graphite-based systems by Raman spectroscopy. *Physical Chemistry Chemical Physics* **2007,** *9* (11), 1276-1290.

(5) Zhang, P.; Fan, J.; Wang, Y.; Dang, Y.; Heumann, S.; Ding, Y. J. C., Insights into the role of defects on the Raman spectroscopy of carbon nanotube and biomass-derived carbon. *Carbon* **2024,** *222*, 118998.

(6) Brubaker, Z. E.; Miskowiec, A.; Niedziela, J. L., Raman spectroscopy of thermally perturbed carbon fibers: Discriminating spectral responses of modulus classes and defect types. *Physical Review Materials* **2022,** *6* (7), 073603.

(7) Jorio, A.; Saito, R., Raman spectroscopy for carbon nanotube applications. *Journal of Applied Physics* **2021,** *129* (2).
